# Research on magnetotelluric signal noise separation based on improved SVD

**DOI:** 10.1371/journal.pone.0334468

**Published:** 2026-11-05

**Authors:** Xingtong Yue, Chang Liu, Aibing Jin, Xiaobo Liu, Liancheng Wang, Huaiyuan Wang, Fuming Qu, Rui Zhou

**Affiliations:** 1 School of Resource and Safety Engineering, University of Science and Technology Beijing, Beijing, China; 2 Institute of Minerals Research, University of Science and Technology Beijing, Beijing, China; 3 Ansteel Beijing Research Institute Co., Ltd., Beijing, China; 4 Ansteel Group Mining Co., Ltd., Anshan, Liaoning, China; 5 Institute of Smart Mining, University of Science and Technology Liaoning, Anshan, China; 6 College of Geoexploration Science and Technology, Jilin University, Changchun, China; Shanghai Jiao Tong University, CHINA

## Abstract

As the impact of cultural noise on magnetotelluric impedance estimation becomes increasingly severe, we propose a magnetotelluric signal-to-noise separation method based on improved Singular Value Decomposition (SVD). Compared to cultural noise, the amplitude of the useful electromagnetic signal is weaker. Based on the difference in amplitude between the noise and the useful signal, the SVD method can separate the noisy components from the original signal. However, traditional SVD struggles to completely extract and remove noise when separating different components, and residual noise often exists at the boundary of noise waveforms. Therefore, by combining the Complementary Ensemble Empirical Mode Decomposition (CEEMD) to improve the SVD method, the goal of effectively separating useful magnetotelluric signals and cultural noise is achieved. The proposed method is applied to synthetic cases and actual data processing. Compared with traditional SVD and CEEMD, the results showed that the proposed method improved the problem of incomplete noise suppression in the SVD method. It achieved good processing results for large-scale noise and impulse noise in low signal-to-noise ratio data, and can reduce the distortion of the apparent resistivity phase curve. Furthermore, compared with the processing results of traditional robust methods, the proposed method exhibits superior noise resistance and restores smoother response curves.

## 1. Introduction

As the global industrialization process continues to advance, the demand for energy resources—particularly minerals and hydrocarbons—has escalated across nations. Consequently, enhancing the reliability of mineral resource exploration has become imperative. Electromagnetic (EM) methods are among the cornerstone techniques in mineral resource detection, capable of achieving resistivity imaging from shallow to deep subsurface levels, which plays a critical role in delineating deep-seated mineral deposits [1]. The transient electromagnetic (TEM) method, characterized by pure secondary field observations and high operational efficiency, covers the necessary frequency bands with a single excitation event, making it widely applicable in metallic ore exploration. The magnetotelluric (MT) sounding method, another key technique for large-scale mineral exploration, infers subsurface resistivity structures by observing mutually orthogonal natural electric and magnetic fields at the surface. Compared to artificial-source EM methods, MT offers advantages such as simpler instrumentation and broader frequency-band coverage [2,3]. However, when measurement sites are located in high-interference environments (e.g., mineralized zones), noise sources such as mining equipment and communication devices near the surface or underground can severely degrade MT data quality. This leads to significant distortions in the Cagniard apparent resistivity-phase curves, which fail to accurately reflect subsurface resistivity characteristics, thereby resulting in erroneous inversion interpretations. Therefore, noise suppression techniques that preserve effective signals have garnered increasing attention among MT researchers globally. Given the intrinsic connection between MT denoising and digital signal processing [4], advancements in information technology have driven rapid progress in these methodologies.

Noise in MT data can be categorized into two types: incoherent noise and coherent noise. Incoherent noise primarily manifests as severe interference in a single channel, without simultaneously affecting both electric and magnetic channels. In contrast, coherent noise affects both electric and magnetic channels simultaneously. In the frequency domain, common methods for processing incoherent noise include least-squares estimation and robust estimation. When the signal and noise are uncorrelated, their cross-power spectrum is zero, allowing least-squares estimation to effectively remove incoherent noise [5,6]. Compared to least-squares estimation, robust estimation [5–8] reduces the impact of outliers by decreasing the weight factor of contaminated impedance tensors. However, robust methods require a large amount of high-quality, effective signals for calibration, imposing strict requirements on data quality and signal-to-noise ratio (SNR), which may not always be feasible in mining districts [9]. Egbert [10] proposed a robust multivariate errors-in-variables (RMEV) estimator framework that optimizes MT impedance estimation through automated incoherent-noise diagnosis and robust multichannel signal coherence extraction. For coherent noise, Gamble et al. [11] proposed the single-site remote reference method, which was further developed by Clarke et al. [12]. However, in areas with strong interference, the quality of data from the remote reference station is often poor, limiting the effectiveness of this method. Ritter et al. [13] introduced a dual-site remote reference method for signal-to-noise separation, significantly improving the performance of remote reference methods in high-noise environments. Despite these advancements, selecting appropriate locations for remote reference stations remains a challenge, and such methods can be economically costly. Furthermore, Egbert [14] utilized array data to reduce bias, improve signal-to-noise ratios, and provide better control over coherent noise in estimates of MT impedances.

Among time-domain processing methods, a reference-based method for denoising through data dependency relationships has been proposed [15]. However, this method faces challenges such as high computational load and difficulty in solving the reference formula. Time-domain MT denoising methods can be classified based on noise morphology: for suppressing large-scale noise, commonly used methods include morphological filtering [16] and subspace enhancement algorithms [17]; for removing sharp impulse noise, the matching pursuit algorithm with redundant dictionaries [18,19] and variational mode decomposition (VMD), which processes different frequency components of the data [20], have shown good performance. However, the types of noise present in actual measured data are complex and varied in morphology. Focusing solely on suppressing large-scale noise (or impulse noise) yields limited improvement. With the development of digital signal processing technology, geophysicists have successively applied wavelet analysis [21–23], the Hilbert-Huang transform (HHT) [24], dictionary learning [25,26], independent component analysis (ICA) [27,28], and neural networks [29] to the field of MT denoising. These methods decompose and analyze signals based on the differences between effective MT signals and noise. However, they face challenges such as the loss of effective signals, incomplete denoising, mode mixing, and difficulty in constructing training libraries. Despite powerful noise suppression capabilities, emerging deep learning frameworks (e.g., CS-ResNet [30]) demand massive, uncontaminated training datasets and high computational loads, severely limiting their field applicability in high-interference mining zones. Meanwhile, dictionary learning methods like K-SVD [31] rely heavily on pre-trained dictionaries, struggling to adapt efficiently to unpredictable, non-stationary impulse noise. Additionally, while advanced mode decomposition algorithms like ICEEMDAN [4,32] effectively mitigate mode mixing, they fail to independently resolve the high-energy boundary mutations characteristic of large-scale noise in severe interference environments.

In high-interference environments, the amplitude energy of noise dominates over effective MT signals, sometimes completely obscuring them. Singular value decomposition (SVD), which exploits amplitude discrepancies to separate distinct signal components, enables the isolation of noise and MT signals from different sources through singular value computation [33,34]. However, when addressing large-scale noise, SVD fails to fully extract noise waveforms due to boundary smoothing artifacts, leaving residual impulsive noise in the processed MT data. To overcome these limitations, this study proposes a data-driven, unsupervised hybrid method combining SVD with complementary ensemble empirical mode decomposition (CEEMD). The proposed method does not require a pre-trained dictionary or large-scale labeled training datasets, and therefore provides a lightweight and unsupervised alternative for MT denoising under limited prior-data conditions. Practically, it implements an efficient two-stage mechanism: SVD primarily isolates high-energy noise based on amplitude discrepancies, while CEEMD secondarily eliminates the residual impulsive noise. This joint strategy resolves both the boundary artifacts of traditional SVD and the edge suppression issues of standalone CEEMD, yielding better signal-to-noise separation in the tested cases. The procedure involves three sequential phases: decomposing noisy signals via SVD; suppressing identified noise and residual impulsive components via CEEMD; and reconstructing denoised signals from the purified components. Validation through synthetic experiments (MT data in the Gansu area) and field datasets (measured data in the Qilian area) confirms that this approach effectively mitigates noise interference, producing continuous and smooth Cagniard apparent resistivity and phase curves. The Gansu area is vast and sparsely populated, and the quality of the MT data is high. Thus, adding random noise to the MT data from the Gansu area can confirm the reliability of the proposed method. Certain measured stations in the Qilian area have been severely affected by heavy industrialization, resulting in poor data quality. Consequently, the effectiveness of the proposed method has been confirmed through processing these measured data. The locations of the survey areas are shown in Fig 1.

**Fig 1:**
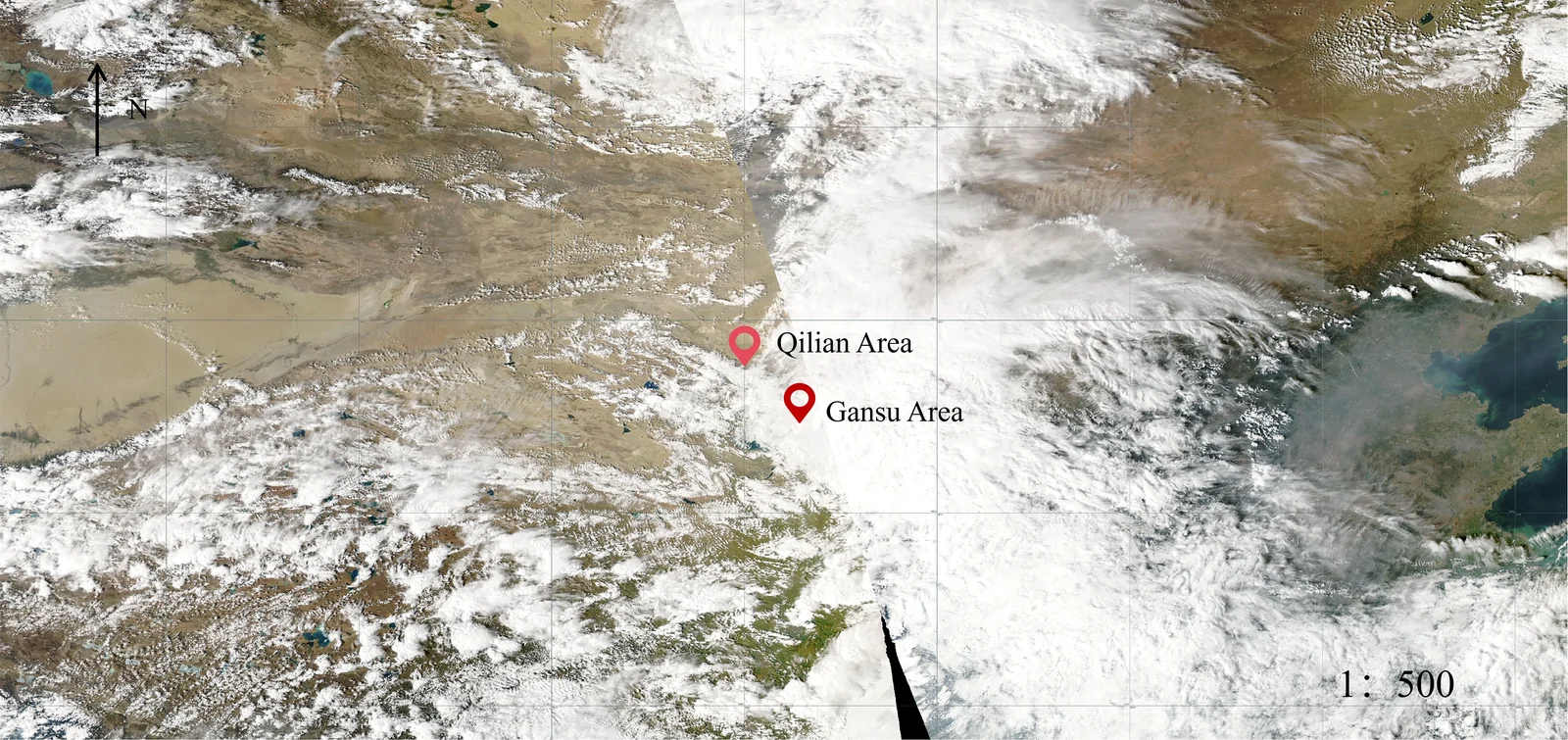
The location of survey areas. The base imagery was obtained from the Aqua/MODIS Corrected Reflectance (True Color) product through NASA Worldview (https://worldview.earthdata.nasa.gov/). Latitude–longitude lines were derived from Natural Earth public-domain data.

## 2. Algorithm principles

### 2.1. Singular value decomposition (SVD)

The time series vectors $x_{i}$ are used to construct a real matrix $X=[x_{\text{1}}^{\text{T}}x_{\text{2}}^{\text{T}}\ldots x_{n}^{\text{T}}]$, where ${X\in \text{R}}^{m\times n}$, SVD is then applied to the matrix *X* [35,36]*:*


$$\begin{array}{c} \begin{array}{c} X={USV}^{\text{T}} \end{array} \end{array}$$ (1)

where X represents the multidimensional input matrix constructed from the original noisy MT signal, and the main diagonal elements of ${S\in \text{R}}^{m\times n}$ are the singular values of the matrix X. The superscript T denotes the transpose operation, p=min(m,n), and $\sigma_{\text{1}}\geq \sigma_{\text{2}}\geq \ldots \geq \sigma_{p}\geq \text{0}\text{for}\sigma_{i}(i=\text{1},\text{2},\ldots ,p)$. *U* is an m×morthogonal matrix of left singular vectors, and *V* is an n×n orthogonal matrix of right singular vectors.

In SVD, the input data must be a multidimensional matrix. Therefore, it is necessary to transform the one-dimensional MT time series into a multidimensional matrix using Hankel matrices. Additionally, the components obtained from SVD can be divided into effective signals and noise:


$$\begin{array}{c} \begin{array}{c} X=X_{\text{s}}+N=[U_{\text{s}}\cdots U_{\text{n}}][\begin{array}{ccc} S_{\text{s}} & \cdots & \text{0}\\ \vdots & \ddots & \vdots \\ \text{0} & \cdots & S_{\text{n}} \end{array}][\begin{array}{c} V_{\text{s}}^{\text{T}}\\ \vdots \\ V_{\text{n}}^{\text{T}} \end{array}] \end{array} \end{array}$$ (2)

where$X_{\text{s}}$ represents the effective signal matrix, and N represents the noise matrix. The subscript s denotes the effective signal components, and the subscript n denotes the noise components in the decomposed singular vectors and values. After identifying and removing the noise components, the effective signal is reconstructed using the inverse SVD process:


$$\begin{array}{c} \begin{array}{c} \overset{―}{X_{\text{s}}}=U_{\text{s}}S_{\text{s}}V_{\text{s}}^{\text{T}} \end{array} \end{array}$$ (3)

where $\overset{―}{X_{\text{s}}}$ is the reconstructed matrix of the effective signal. Finally, to recover the one-dimensional denoised MT time series from the two-dimensional matrix $\overset{―}{X_{\text{s}}}$, diagonal averaging is applied.

### 2.2. Complementary ensemble empirical mode decomposition (CEEMD)

Based on Empirical Mode Decomposition (EMD) [37] and Ensemble Empirical Mode Decomposition (EEMD) [38], Complementary Ensemble Empirical Mode Decomposition (CEEMD) overcomes issues such as mode mixing and residual Gaussian white noise [32]. CEEMD adds two sets of Gaussian white noise with the same amplitude but opposite phases to the original signal (the number of additions is denoted as *i*) and decomposes the signal into intrinsic mode functions (IMFs) at different scales. The processing steps for MT signals are as follows:

First, a single-channel MT signal to be analyzed is denoted by *x*. A total of *I* Gaussian white-noise realizations are added to the signal, where *I* is set to 100 based on the parameter sensitivity analysis presented in S1 Appendix. The resulting noise-assisted sequences are constructed as follows:


$$\begin{array}{c} \begin{array}{c} x^{\left(i\right)}=x+\epsilon_{\text{1}}E_{\text{1}}\left(w^{\left(i\right)}\right) \end{array} \end{array}$$ (4)

where *w*^(*i*)^ represents the *i*-th set of Gaussian white noise (i=1,2,3,…,I) and *E*_1_() denotes the first-order component produced by the EMD method. *ε*_1_ represents the weight coefficient of the noise component *E*_1_(*w*^(*i*)^) (its value is defined as the ratio of the signal-to-noise ratio of the added noise to the standard deviation of this noise component). Based on Equation (4), the sequence *x*^(*i*)^ is obtained to derive the first-order IMF component.


$$\begin{array}{c} \begin{array}{c} {c_{\text{1}}}^{\left(i\right)}=x-\text{mean}\left(x^{\left(i\right)}\right) \end{array} \end{array}$$ (5)

$$\begin{array}{c} \begin{array}{c} C_{\text{1}}=\frac{\text{1}}{I}\sum_{i=\text{1}}^{I}c_{\text{1}}^{\left(i\right)} \end{array} \end{array}$$ (6)

where the operator mean(.) represents the local envelope mean calculation. *c*_1_^(*i*)^ denotes the first-order IMF component of the corresponding *x*^(*i*)^, and *C*_1_ represents the final first-order IMF component of the original signal *x*. Based on *C*_1_, the residual of the first-order decomposition can be obtained:


$$\begin{array}{c} \begin{array}{c} r_{\text{1}}=x-C_{\text{1}} \end{array} \end{array}$$ (7)

The residual of the decomposed components is taken as the new signal to be analyzed. White noise is added to this residual, and the second-order IMF residual *r*_2_ and the second-order IMF component *C*_2_ are obtained as follows:


$$\begin{array}{c} \begin{array}{c} r_{\text{2}}=\frac{\text{1}}{I}\sum_{i=\text{1}}^{I}\text{mean}\left(r_{\text{1}}+\epsilon_{\text{2}}E_{\text{2}}(w^{\left(i\right)})\right) \end{array} \end{array}$$ (8)

$$\begin{array}{c} \begin{array}{c} C_{\text{2}}=r_{\text{1}}-r_{\text{2}} \end{array} \end{array}$$ (9)

where $E_{\text{2}}$ denotes the second-order component produced by the EMD method, and $\epsilon_{\text{2}}$ is the weight coefficient of the added noise for the second order. With Equations (8) and (9), the *k*-th order decomposition residual *r*_*k*_ and the *k*-th order IMF component *C*_*k*_ can be derived, as follows:


$$\begin{array}{c} \begin{array}{c} r_{k}=\frac{\text{1}}{I}\sum_{i=\text{1}}^{I}\text{mean}\left(r_{k-\text{1}}+\epsilon_{k}E_{k}(w^{\left(i\right)})\right) \end{array} \end{array}$$ (10)

$$\begin{array}{c} \begin{array}{c} C_{k}=r_{k-\text{1}}-r_{k} \end{array} \end{array}$$ (11)

where *k* = 3,4,⋯,*K*, and *K* represents the total number of IMF components. $E_{k}$ denotes the *k*-th order component produced by the EMD method, and $\epsilon_{k}$ is the weight coefficient of the added noise for the *k*-th order. Repeat Equations (10) and (11) until the *k*-th order decomposition residual *r*_*k*_ becomes a monotonic signal, at which point the sifting process terminates. In summary, the steps of the proposed method are as follows (Fig 2):

**Fig 2:**
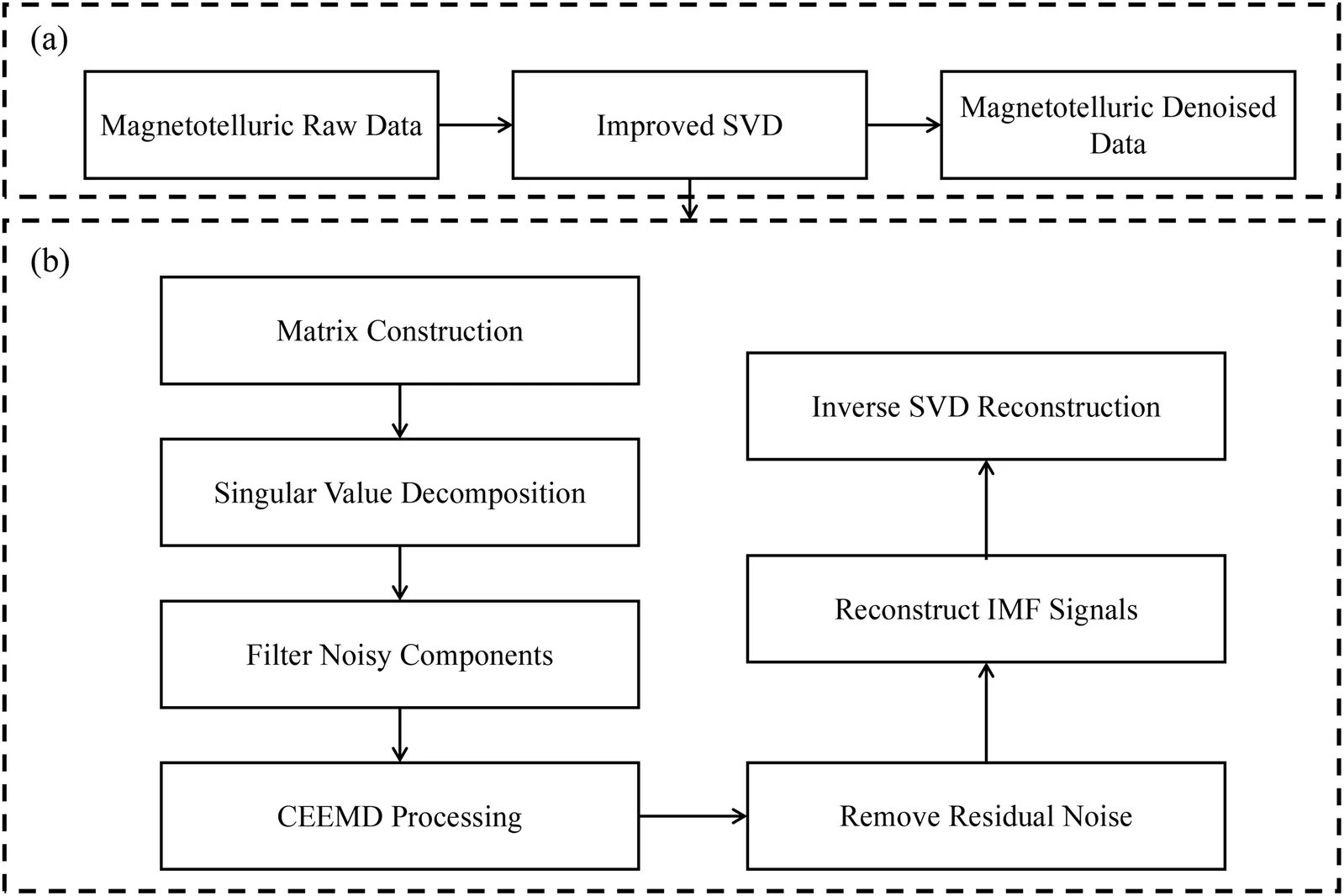
Flowchart of the proposed method.

1. Construct the data into a multidimensional matrix to satisfy the requirements for component decomposition;
2. Perform SVD on the aforementioned data matrix to obtain each signal component;
3. Analyze each component and filter out the noisy components, thereby removing them from the original signal;
4. Further decompose the data using CEEMD to suppress residual noise;
5. Finally, reconstruct the effective geomagnetic signal using the denoised components.

## 3. Simulation experiment analysis

Large-scale noise and complex interferences (such as square waves and impulse noise) are added to the low-noise MT signals. The results of different denoising methods are then compared to verify the effectiveness and robustness of the proposed approach. For the synthetic tests, since the clean reference signal is known prior to the addition of artificial noise, the denoising performance can be quantitatively evaluated by comparing the denoised signal against this reference. The signal-to-noise ratio (SNR), normalized cross-correlation (NCC), fitting error E, and mean squared error (MSE) are used to quantitatively analyze the improvement effects of the processing methods. These parameters are defined as follows [39,40]:


$$\begin{array}{c} \begin{array}{c} \text{SNR}=\text{20}\text{l}\text{g}\frac{{\left\|y(n)\right\|}_{\text{2}}}{{\left\|y(n)-x(n)\right\|}_{\text{2}}} \end{array} \end{array}$$ (12)

$$\begin{array}{c} \begin{array}{c} \text{NCC}=\frac{\sum_{n=\text{1}}^{N}y(n)\cdot x(n)}{\sqrt{\left(\sum_{n=\text{1}}^{N}y^{\text{2}}(n)\right)}\cdot \sqrt{\left(\sum_{n=\text{1}}^{N}x^{\text{2}}(n)\right)}} \end{array} \end{array}$$ (13)

$$\begin{array}{c} \begin{array}{c} \text{E}=\frac{{\left\|y(n)-x(n)\right\|}_{\text{2}}}{{\left\|y(n)\right\|}_{\text{2}}} \end{array} \end{array}$$ (14)

$$\begin{array}{c} \begin{array}{c} \text{MSE}=\frac{{\sum_{n=\text{1}}^{N}\left(y(n)-x(n)\right)}^{\text{2}}}{N} \end{array} \end{array}$$ (15)

where *x(n)* represents the reference clean MT signal (i.e., the baseline low-noise signal prior to adding synthetic noise), *y(n)* denotes the denoised signal output by the processing method, and n is the discrete sample index, *N* is the total number of samples, and ${\left\|\cdot \right\|}_{\text{2}}$ denotes the L2 norm of the vector. The artificial noisy signal is used solely as the input for the denoising procedures and is not used as a reference for metric calculation. Therefore, a better denoising effect is characterized by higher SNR and NCC values, corresponding to lower E and MSE values.

Fig 3 illustrates the process of generating synthetic noisy signals. Clean MT measured data (collected in the Gansu area, with sampling rate at 15 Hz) serves as the basis for the synthesis process, and different noises are added to generate noisy signals. The sparse population in the Gansu area results in minimal anthropogenic noise, ensuring high-quality MT data acquisition. As observed in Fig 3a, the original signal is stable without any noise waveform. Fig 3d to 3f depict the noisy signals with different types of noise added, such as square-wave noise, impulse noise and mixed noise. It can be seen from Fig 3d to 3f that the morphology of the original MT signal is almost completely obscured, making the time series no longer continuous and smooth. Meanwhile, the amplitude of the original signal was changed (the effective signal energy is significantly lower than the noise energy), making it difficult to obtain accurate impedance results.

**Fig 3:**
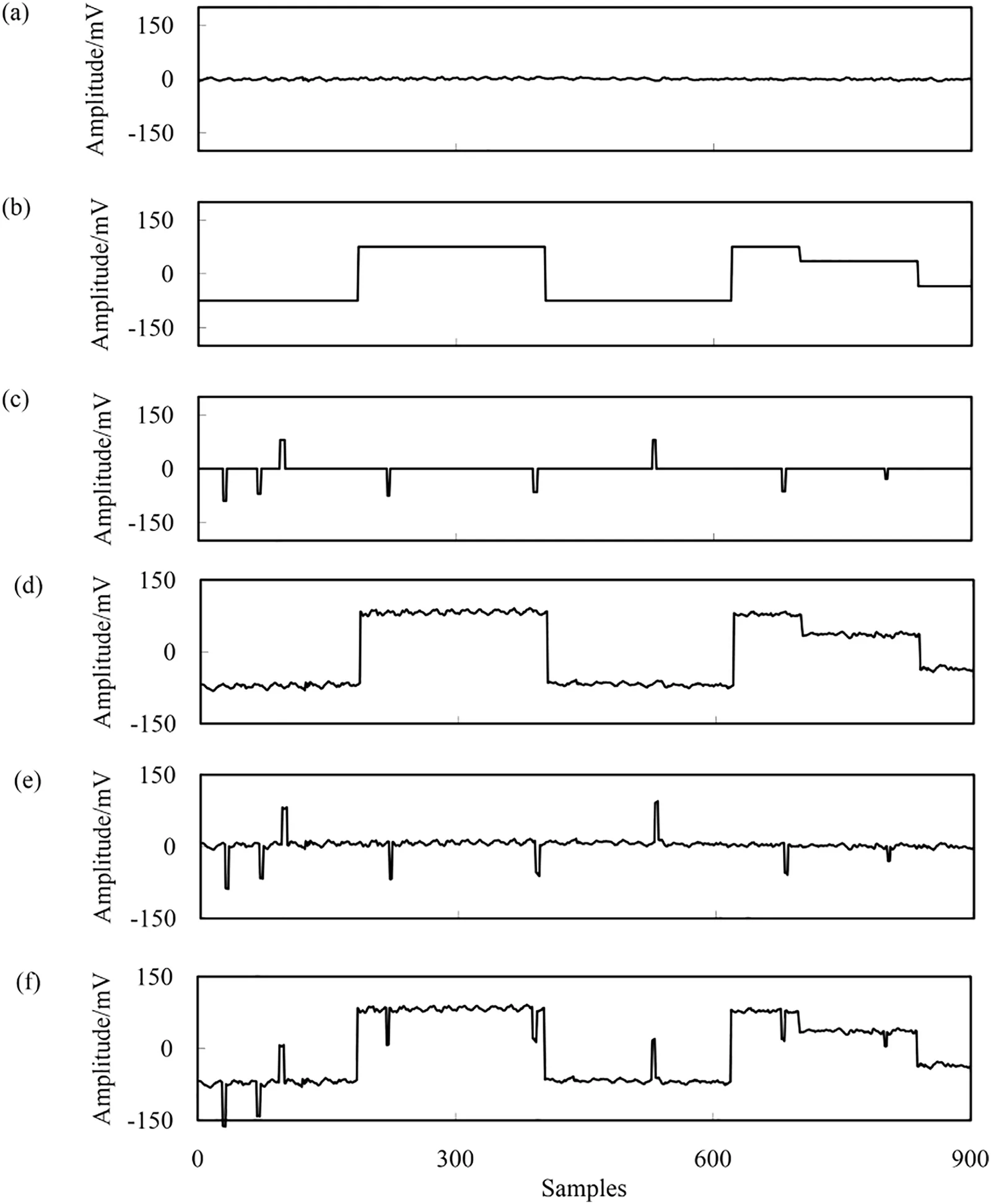
Comparison of effective and noisy magnetotelluric signals. (a) Original MT time series, (b) large-scale noise, (c) impulse noise, (d) noisy signal containing large-scale noise, (e) noisy signal containing impulse noise, (f) noisy signal containing various noise. The original MT signals were collected in the Gansu region with a sampling frequency of 15 Hz, and all noise was randomly generated.

Fig 4 shows the corresponding spectra of the original signal and noisy signals in Fig 3. By comparing Fig 4a with Fig 4b and 4d, it is evident that the addition of large-scale noise not only increases the amplitude of the main frequency peak of the effective signal but also introduces multiple side peaks and bandwidths around the main peak, with the amplitude of these side peaks decreasing progressively. This leads to a bias in impedance estimation. From Fig 4a and 4c, it can be seen that impulse noise does not cause a sudden increase in the spectral amplitude but continuously interferes with the entire spectrum.

**Fig 4:**
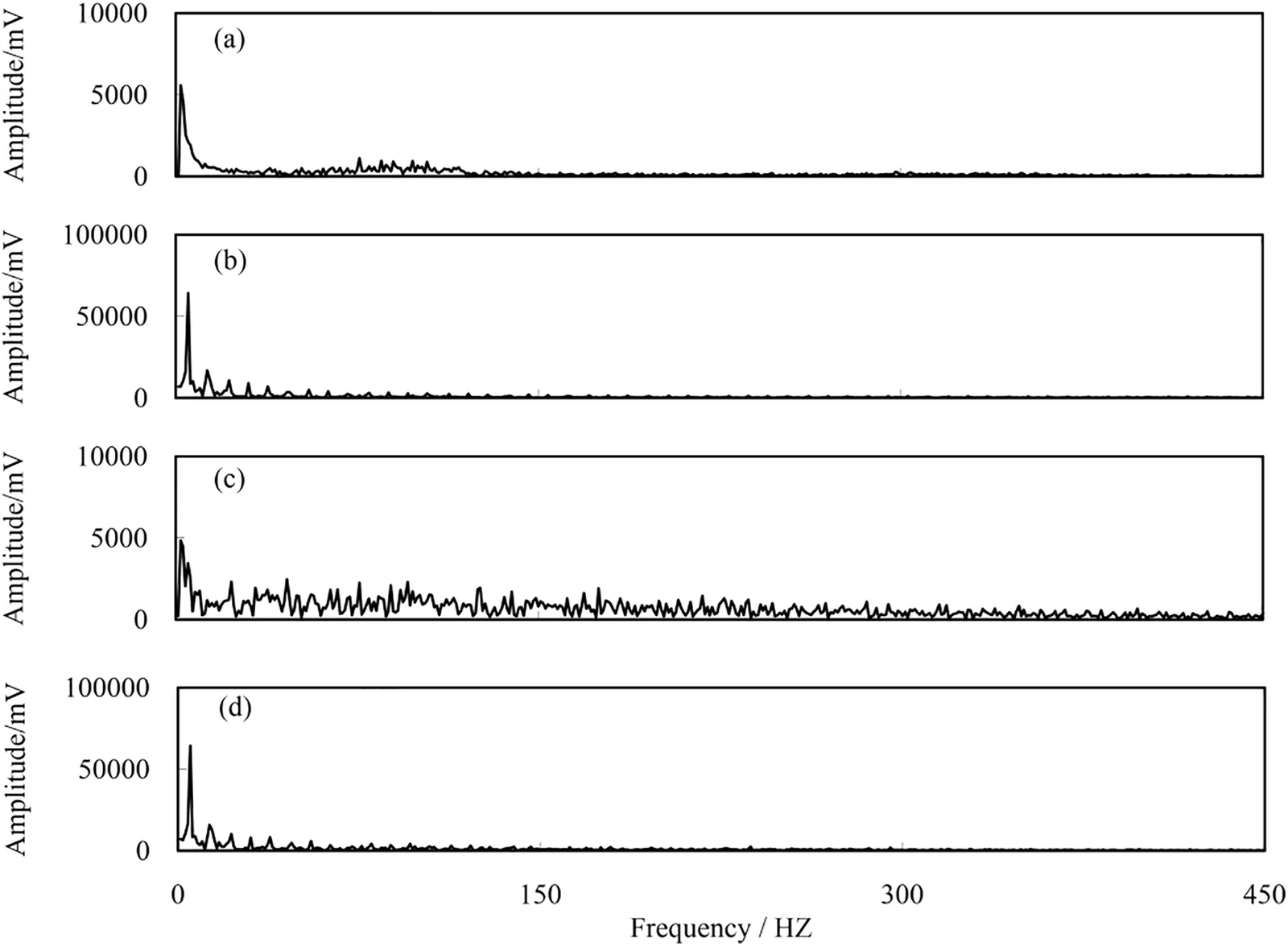
Spectral information of natural magnetotelluric signals and noisy signals. (a) Original MT frequency spectrum, (b) noisy signal containing large-scale noise, (c) noisy signal containing impulse noise, (d) noisy signal containing various noise types.

The denoising results of different noisy signals using traditional SVD, CEEMD, and the proposed method are shown in Fig 5. From Fig 5a to 5c, it can be observed that traditional SVD can reduce the scale of large-scale noise, transforming wide square waves into narrower ones, but it fails to completely suppress the morphology of large-scale noise. Additionally, while SVD reduces the amplitude of abrupt noise in the data segment 0–300 in Fig 5b, its improvement effect on the segment 300–600 is relatively weak, indicating poor robustness of SVD in removing abrupt noise, especially impulse noise. Fig 5d to 5f show the processing results using the CEEMD method. It is evident that this method can suppress noise morphology, but the fluctuations in the effective signal differ significantly from those in the original signal, leading to a loss of the effective signal. The denoised signals processed by the proposed method are illustrated in Fig 5g to 5i. In comparison, the proposed method not only suppresses noise waveforms but also demonstrates improvement in handling abrupt noise. However, due to the small amplitude difference between the residual scale noise and the effective signal in Fig 5d and 5f, further signal-to-noise separation is not performed to avoid excessive loss of the effective signal. As a result, the processed signal morphology cannot be made completely identical to that of the effective signal.

**Fig 5:**
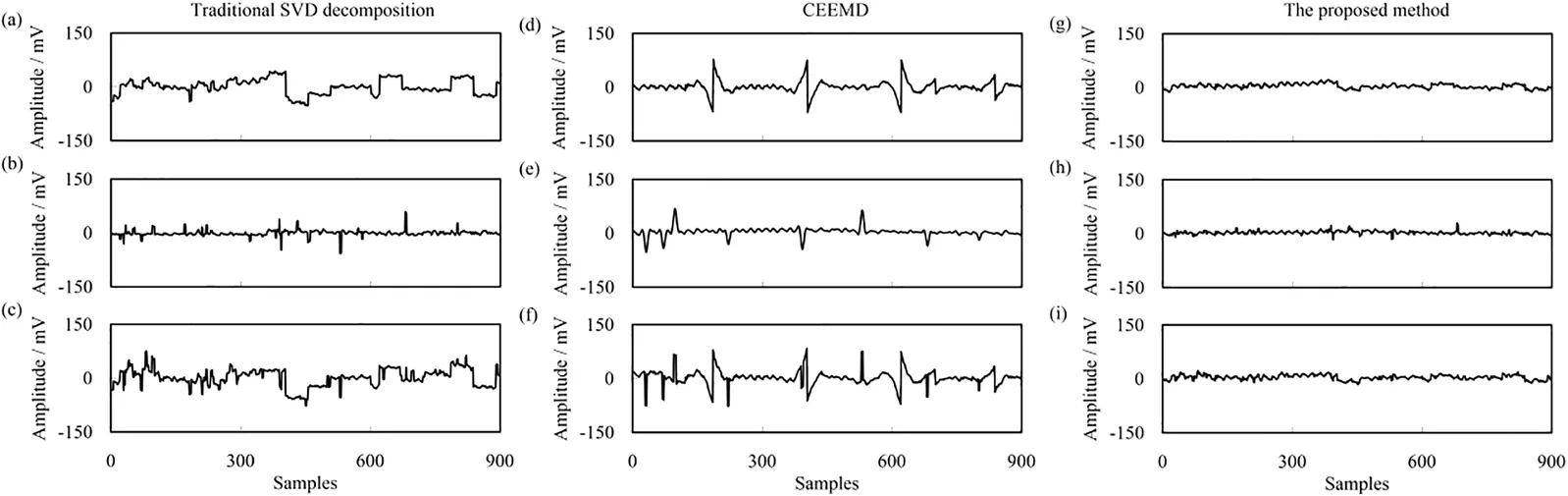
Comparison of time series after denoising by different methods. (a)–(c) Time series comparisons after removing square-wave noise, impulse noise, and mixed noise using traditional SVD; (d)–(f) time series comparisons using the CEEMD method; and (g)–(i) time series comparisons using the proposed method.

Fig 6 shows the spectral plots of the signals after denoising using different methods. Fig 6a–6c display the spectra after traditional SVD, Fig 6d–6f show the spectra after CEEMD denoising, and Fig 6g–6i present the spectral information after noise suppression using the proposed method. From Fig 6a–6c, it can be seen that while traditional SVD reduces the amplitude of the noisy signal spectrum to some extent, there is still a nearly fourfold difference between the main frequency peak values compared to the original spectrum. Additionally, multiple secondary frequency peaks in the tail region remain, which can still affect subsequent transfer function calculations. In contrast, after processing with CEEMD, although large-scale noise and impulse noise are suppressed, there is significant attenuation of the main frequency signal, resulting in a loss of much of the effective signal. Compared to these methods, the proposed method yields spectral results most similar to the original signal. However, in the case of the processed signal containing abrupt noise (Fig 6e), the main frequency peak is slightly lower than that of the original signal, but the difference is minimal.

**Fig 6:**
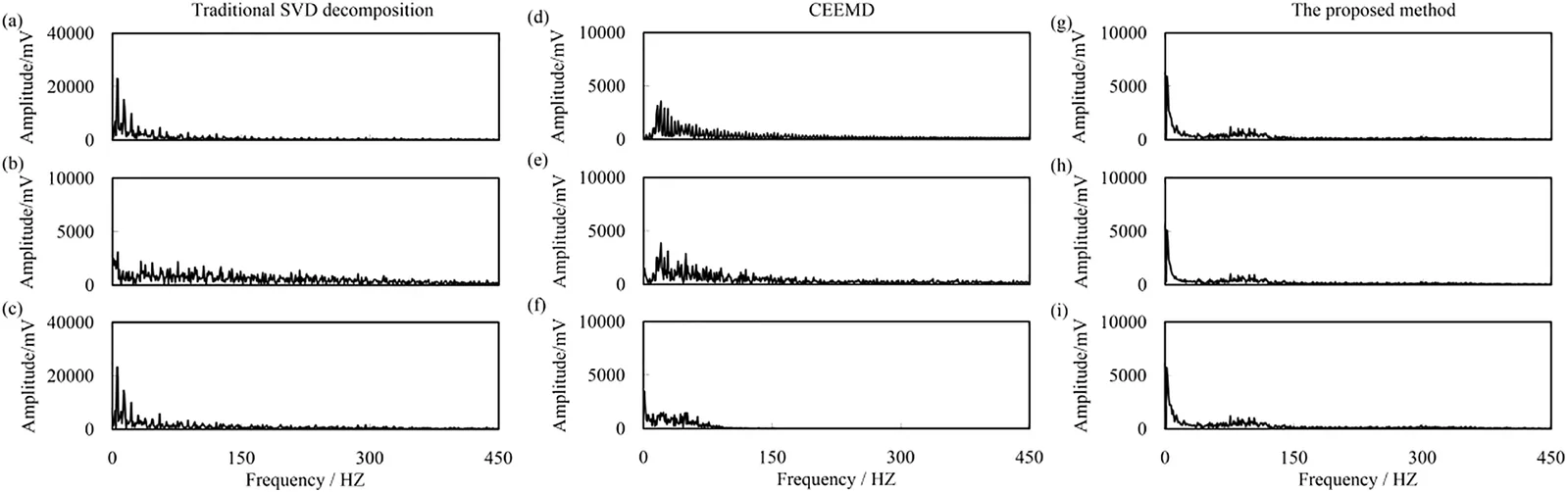
Comparison of frequency spectrum after suppressing different noises using different methods. (a), (d) and (g) represent the spectral comparisons of traditional SVD, the CEEMD method, and the proposed method after removing square-wave noise, respectively. (b), (e), and (h) represent the spectral comparisons of different methods after removing impulse noise. (c), (f) and (i) represent the spectral comparisons of different methods after removing mixed noise.

The evaluation of the time series properties of the denoised signals using different methods is shown in Fig 7, where the black solid line represents the traditional SVD method, the white dashed line represents the CEEMD method, and the red solid line represents the proposed method. Fig 7a shows the signal-to-noise ratio (SNR) of the data, Fig 7b shows the data error (E), Fig 7c shows the normalized cross-correlation (NCC), and Fig 7d shows the mean squared error (MSE) of the data. Across different noise conditions, the four evaluation parameters of the proposed method are all superior to those of the traditional SVD and the CEEMD method. Fig 8 presents the evaluation of the spectral properties of the denoised signals using different methods, corresponding to Fig 7. To quantitatively evaluate the denoising performance, the numerical metrics of the different methods under various noise conditions have been annotated in Figs 7 and 8. These specific values provide a direct and objective comparison of the improvements achieved by each method. Comparing Figs 7d and 8d reveals that smaller error values in the time series result in differences of up to two orders of magnitude in the frequency spectrum. Additionally, combining Figs 7 and 8, it is evident that the three methods perform better in suppressing abrupt noise compared to handling large-scale noise and mixed noise types.

**Fig 7:**
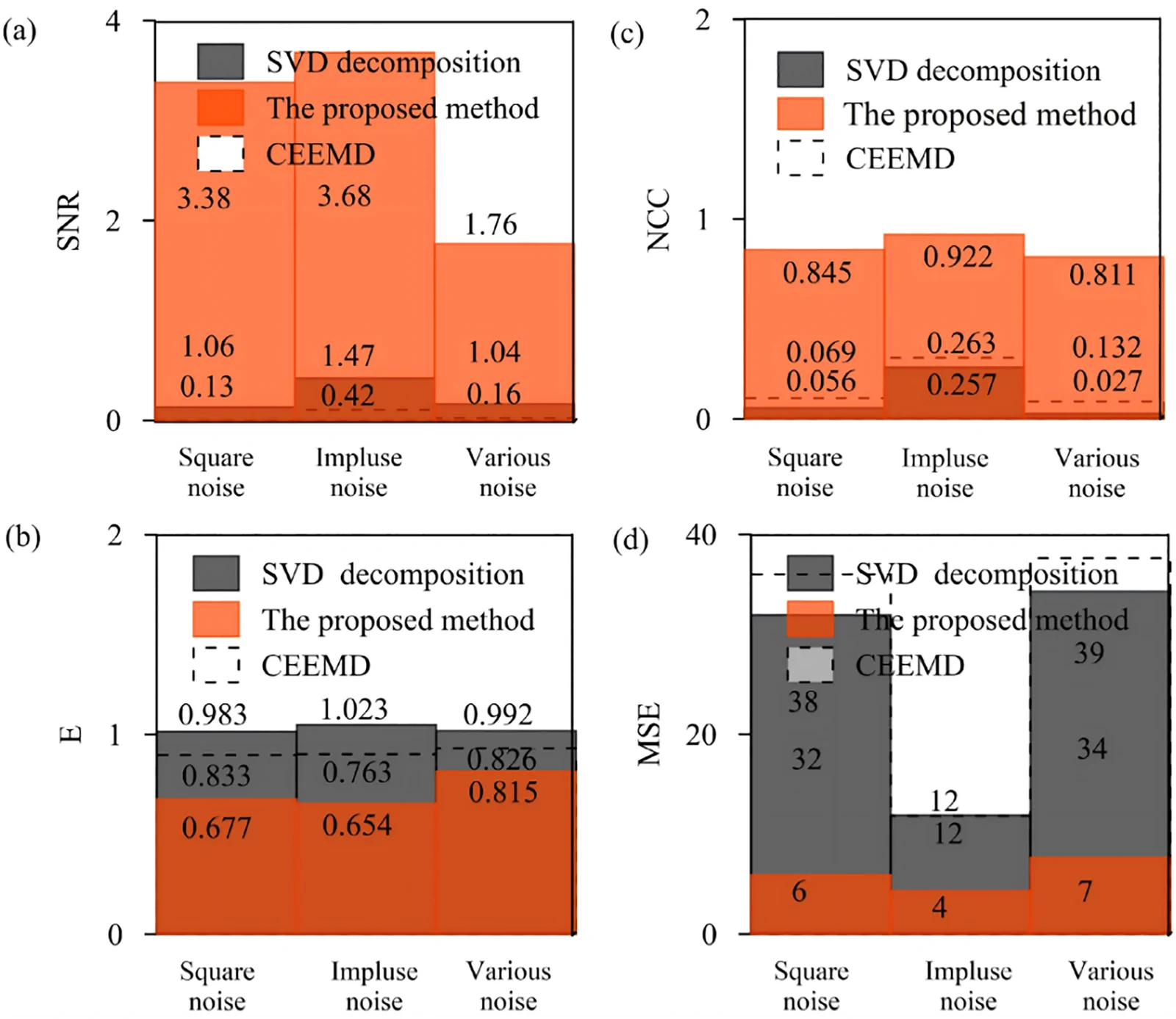
Evaluation of denoised signals after suppressing different noises. (a), (b), (c), and (d) represent the signal-to-noise ratio (SNR), data error (E), normalized cross-correlation (NCC), and mean squared error (MSE) of the data processed by different methods, respectively. The black solid line represents the traditional SVD method, the white dashed line represents the CEEMD method, and the red solid line represents the proposed method. The numerical values above the lines indicate the specific quantitative metrics obtained by each method.

**Fig 8:**
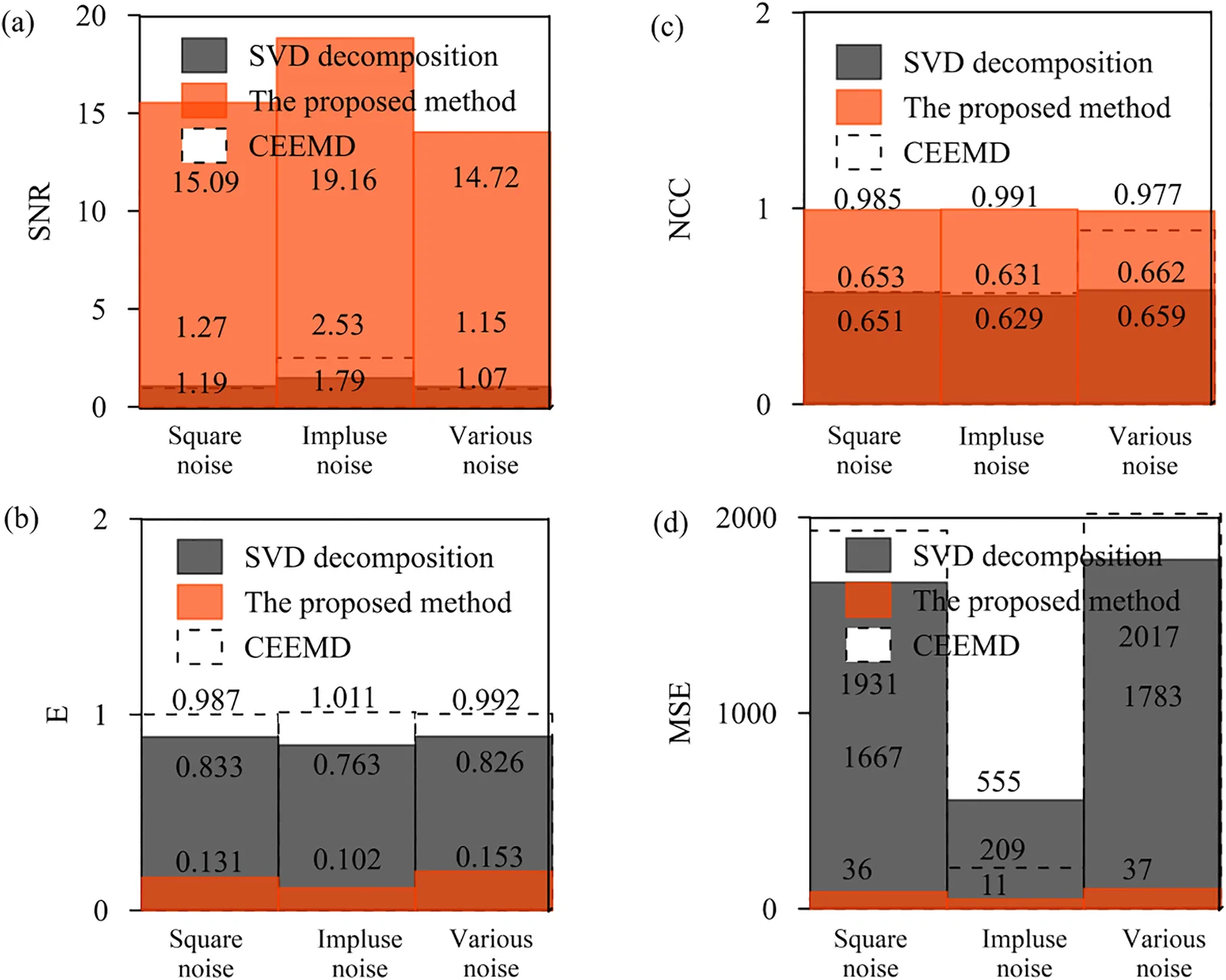
Evaluation of spectral properties before and after denoising. (a), (b), (c), and (d) correspond to the evaluation metrics shown in Fig 7, respectively. The black solid line represents the traditional SVD method, the white dashed line represents the CEEMD method, and the red solid line represents the proposed method. The numerical values above the lines indicate the specific quantitative metrics obtained by each method.

In field measurements, multiple types of noise typically degrade the quality of MT data collectively. As an example of this scenario, Fig 9 shows the apparent resistivity and phase curves calculated from the original signal, the signal contaminated with mixed noise, and the signals denoised using different methods. Fig 9a displays the apparent resistivity and phase curves derived directly from the original signal. Fig 9b shows the curves after introducing mixed noise, and Fig 9c, 9d, and 9e represent the curves after processing with traditional SVD, CEEMD, and the proposed method, respectively. The amplitude of the frequency points in the apparent resistivity curve in Fig 9b increases by approximately one order of magnitude, and the phase curve *p*_*yx*_ distorts in the 0–1 Hz frequency band. Regarding the improvement results shown in Fig 9c, traditional SVD increases the amplitude of the low-frequency tail of the apparent resistivity curve while smoothing out the jump points; conversely, the CEEMD method (Fig 9d) aligns the trend of the *yx*–direction apparent resistivity and phase curves with the original signal but introduces more _*xy*_ jump points. In contrast, the results in Fig 9e are smoother, with only a few frequency points remaining inconsistent with the original curve in the 1–0.1 Hz frequency band.

**Fig 9:**
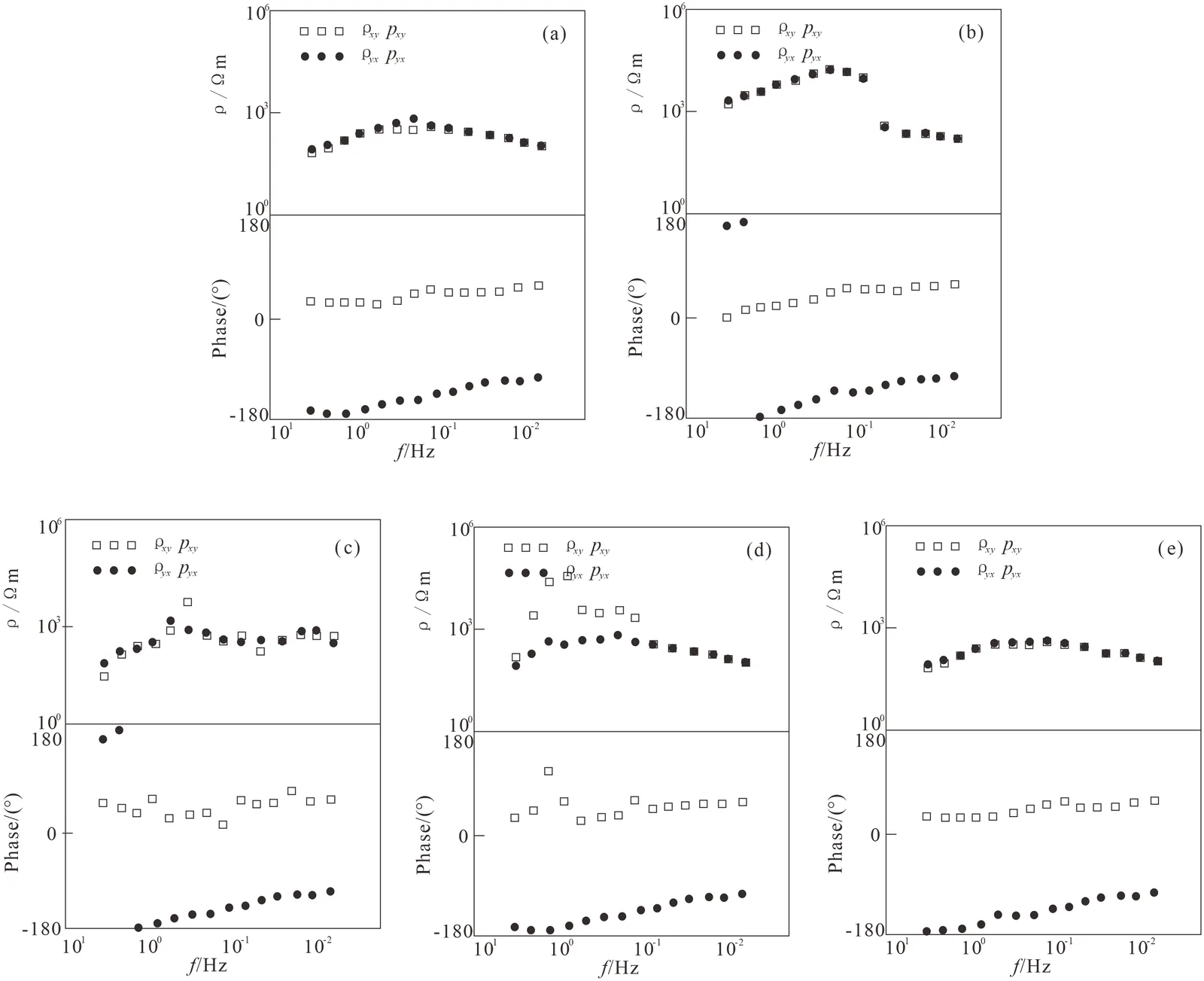
The apparent resistivity and phase curves calculated from the original and denoised signals. (a) The apparent resistivity and phase curves of the original data; (b) the apparent resistivity and phase curves of the noisy signal; (c) the apparent resistivity and phase curves of the denoised signal after traditional SVD; (d) the apparent resistivity and phase curves of the denoised signal obtained by CEEMD; (e) the apparent resistivity and phase curves of the denoised signal processed by the proposed method.

## 4. Analysis of real data processing

To verify the practicality of the proposed method, the denoising effectiveness is evaluated by processing real MT data collected in the Qilian region. The sampling frequency is 15 Hz, and the distance between adjacent stations is 10 km, with observations lasting over ten hours. Due to the low level of urbanization in the survey area, most stations are minimally affected by anthropogenic noise, resulting in high-quality data. However, stations near industrial areas still exhibit abnormal waveforms in the time series, such as square waves and impulses, leading to anomalies in the apparent resistivity and phase calculations. The locations of MT measured stations are shown in Fig 10.

**Fig 10:**
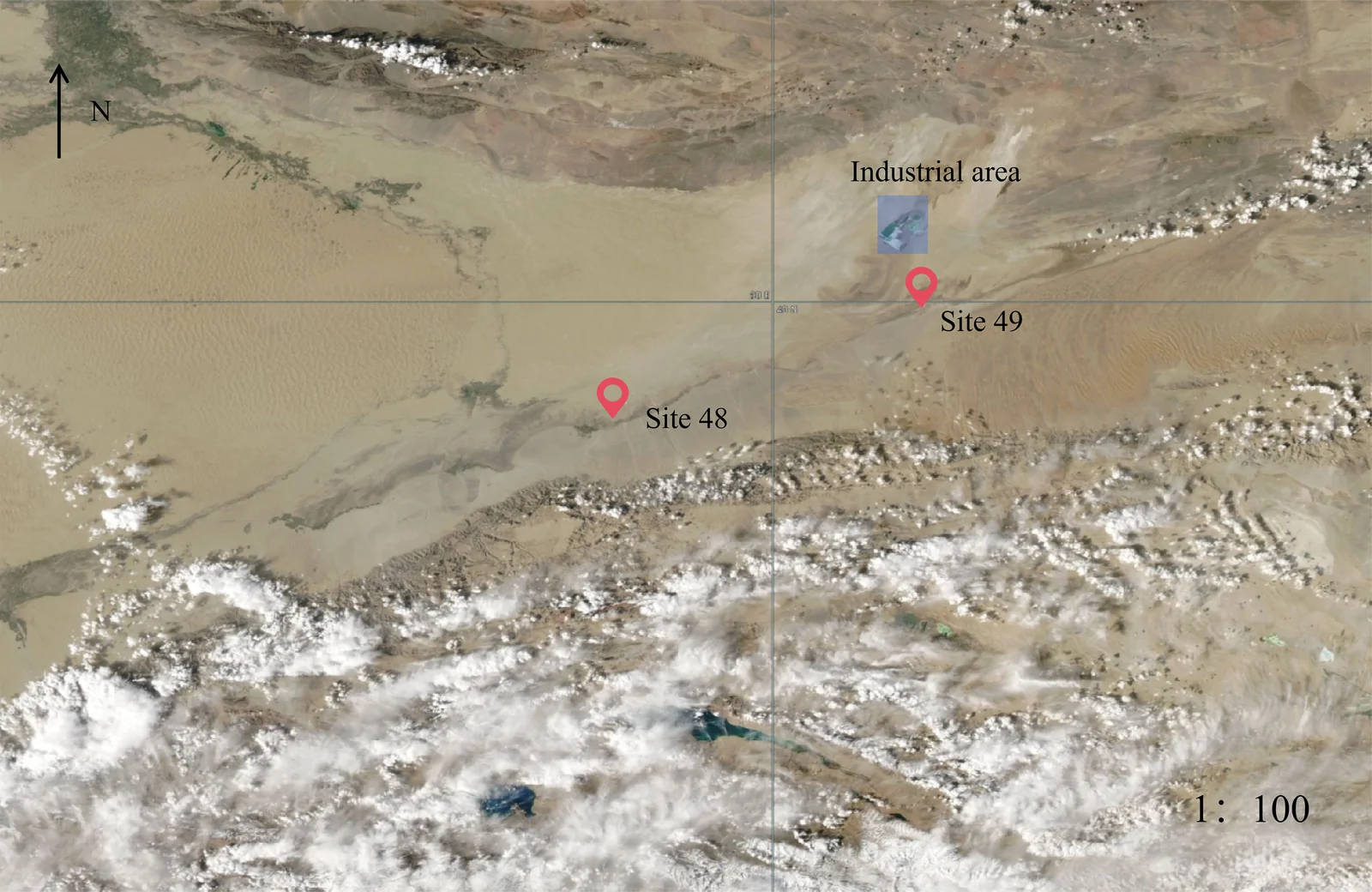
The locations of MT measurement stations. The base imagery was obtained from the Terra/MODIS Corrected Reflectance (True Color) product via NASA Worldview (https://worldview.earthdata.nasa.gov/), and the latitude–longitude lines were obtained from Natural Earth.

Two stations exhibiting representative noise characteristics, Sites 48 and 49, were selected for detailed analysis. Fig 11 shows the original time series and frequency spectra of the MT data from Site 48 and Site 49, where abnormal waveforms are evident in both time series. At Site 49, the sudden addition of square-wave noise significantly increases the amplitude of the effective signal, making it difficult to discern the effective signal from the noise waveform in the time series. Fig 11c presents the spectral information of the original signal at Site 49, revealing multiple secondary peaks in the tail of the spectrum, which decay in a quasi-periodic manner as frequency increases. This spectrum differs markedly from that of MT signals unaffected by noise.

**Fig 11:**
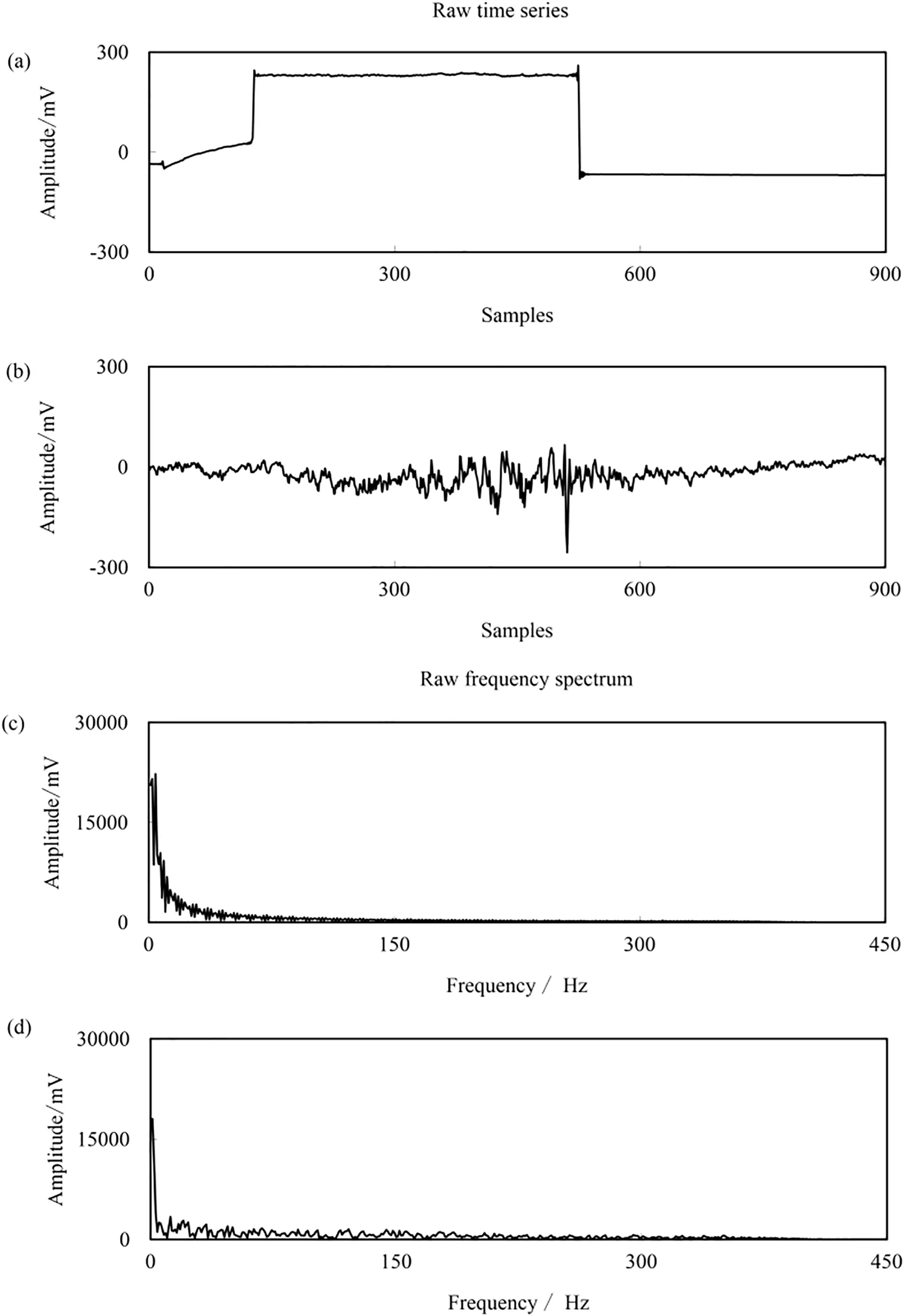
Original time series and frequency spectra of MT data from Site 48 and Site 49. (a) Site 49 raw time series, (b) Site 48 raw time series, (c) frequency spectrum of Site 49 raw data, (d) frequency spectrum of Site 48 raw data.

The original signal time series at Site 48, shown in Fig 11b, exhibits severe triangular-wave and impulse interference in the data segment 150–600. Since only a portion of this segment is impacted by abrupt noise, the spectral amplitude and signal morphology do not show obvious abnormalities, as seen in Fig 11d. However, when the amplitude of the impulse noise increases and persists for longer periods, the spectrum will inevitably be distorted.

The processing results of Site 49 data using traditional SVD, CEEMD, and the proposed method are shown in Fig 12. In each pair of panels, the raw signal and its spectrum are overlaid with the corresponding denoised results. Fig 12b, 12d, and 12f present the frequency-domain comparisons before and after denoising. From these results, it can be observed that the quasi-periodic frequency peaks have been reduced but not completely eliminated. Traditional SVD and CEEMD can suppress large-scale noise, but SVD only partially reduces the amplitude of large-scale noise, leading to the appearance of multiple small square waveforms. CEEMD demonstrates better suppression of large-scale noise, but residual noise remains at the abrupt transitions of large-scale noise. In contrast, the proposed method achieves superior denoising performance. Although fluctuations in large-scale waveforms are still visible in the time series (Fig 12e), the spectrum of the denoised signal in Fig 12f shows no quasi-periodic decaying peaks. The large-scale noise is substantially suppressed, demonstrating the effectiveness of the proposed method in removing such noise.

**Fig 12:**
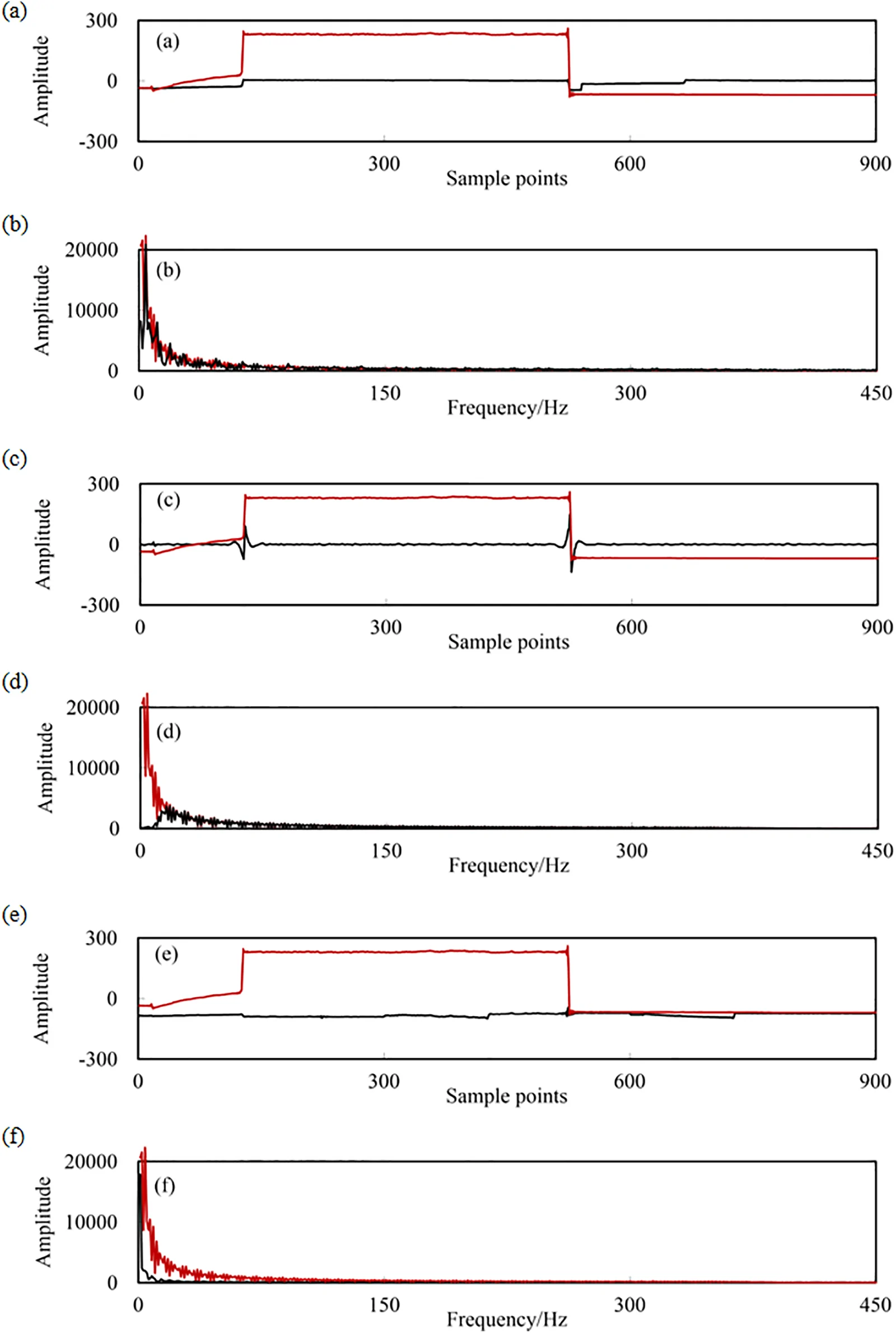
Direct comparison of the raw and denoised signals at Site 49 in the time and frequency domains. The raw signal and its spectrum are shown in red, whereas the denoised results are shown in black. Panels (a) and (b) present the results obtained using conventional SVD; panels (c) and (d) present the results obtained using CEEMD; and panels (e) and (f) present the results obtained using the proposed method. Panels (a), (c), and (e) show the time-domain signals, while panels (b), (d), and (f) show the corresponding amplitude spectra.

The processing results of Site 48 data using different methods are shown in Fig 13. The raw signal and its spectrum are overlaid with the corresponding denoised results in each pair of panels. Fig 13a reveals that traditional SVD can effectively suppress smaller-scale, high-amplitude noise, such as the narrow triangular waves in the data segment 150–400. After SVD processing, this segment appears relatively stable. However, SVD performs poorly in suppressing abrupt noise around data point 500. In contrast, CEEMD shows weaker improvement for the narrower triangular-wave noise (Fig 13c and 13d). Fig 13e and 13f illustrate the processing effects of the proposed method on Site 48 data. It is evident that the proposed method makes the time series of the signal more stable. Since the noise affecting Site 48 causes minimal damage to the data spectrum, the amplitude of the main frequency peak after processing with the proposed method remains largely consistent with the original signal, with only a slight reduction in amplitude in the tail region. In summary, the proposed method achieves better overall performance in stabilizing the time series and maintaining the spectral characteristics of the original signal.

**Fig 13:**
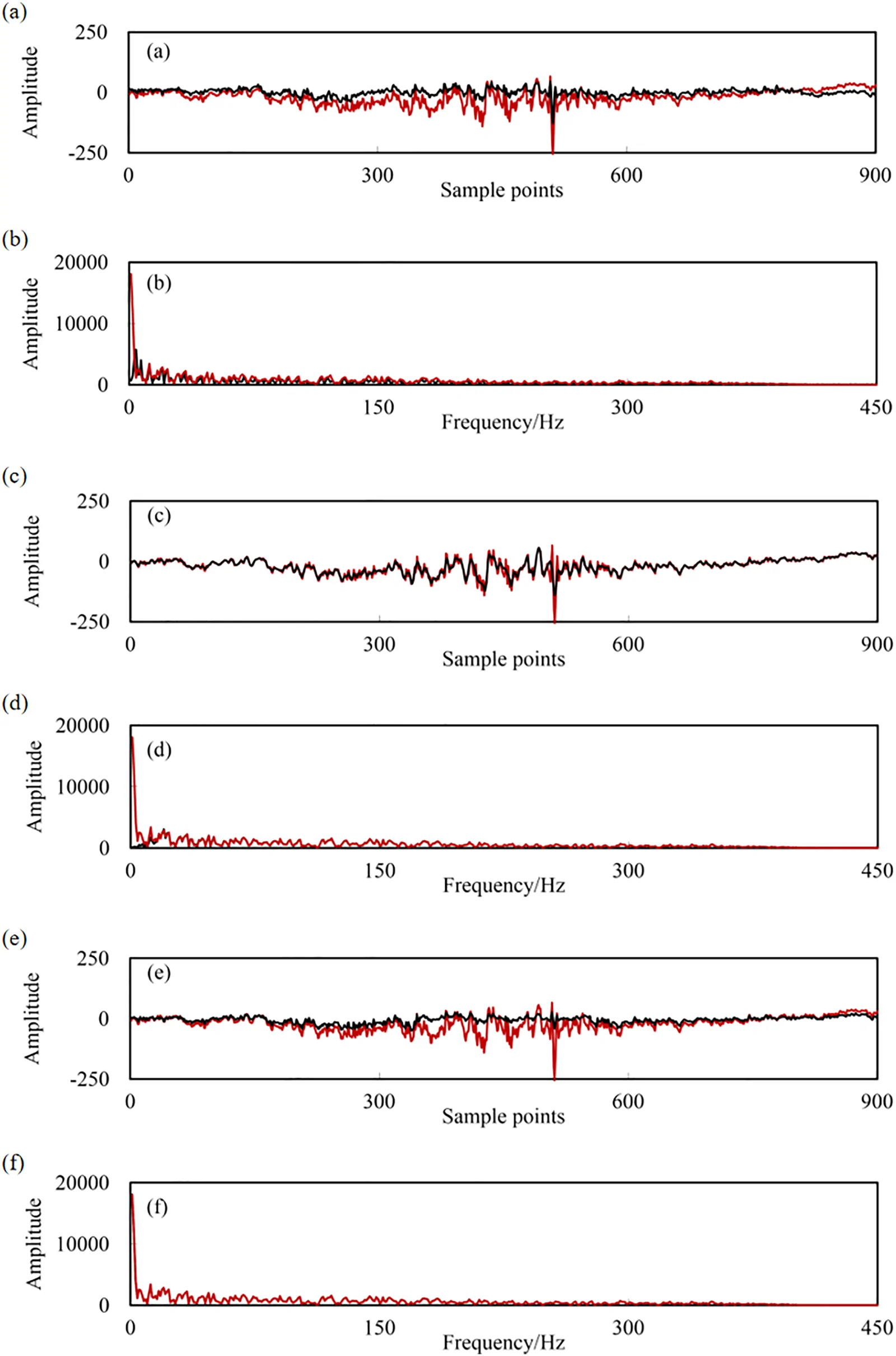
Direct comparison of the raw and denoised signals at Site 48 in the time and frequency domains. The raw signal and its spectrum are shown in red, whereas the denoised results are shown in black. Panels (a) and (b) present the results obtained using conventional SVD; panels (c) and (d) present the results obtained using CEEMD; and panels (e) and (f) present the results obtained using the proposed method. Panels (a), (c), and (e) show the time-domain signals, while panels (b), (d), and (f) show the corresponding amplitude spectra.

To further examine the applicability of the proposed method to different MT components, the Ex, Ey, Hx, and Hy channels at Site 48 were processed using the proposed method, as shown in Fig 14. The results demonstrate that the proposed processing workflow is applicable to both electric and magnetic field components, thereby extending the field-data evaluation from a single component to the four routinely recorded MT channels.

**Fig 14:**
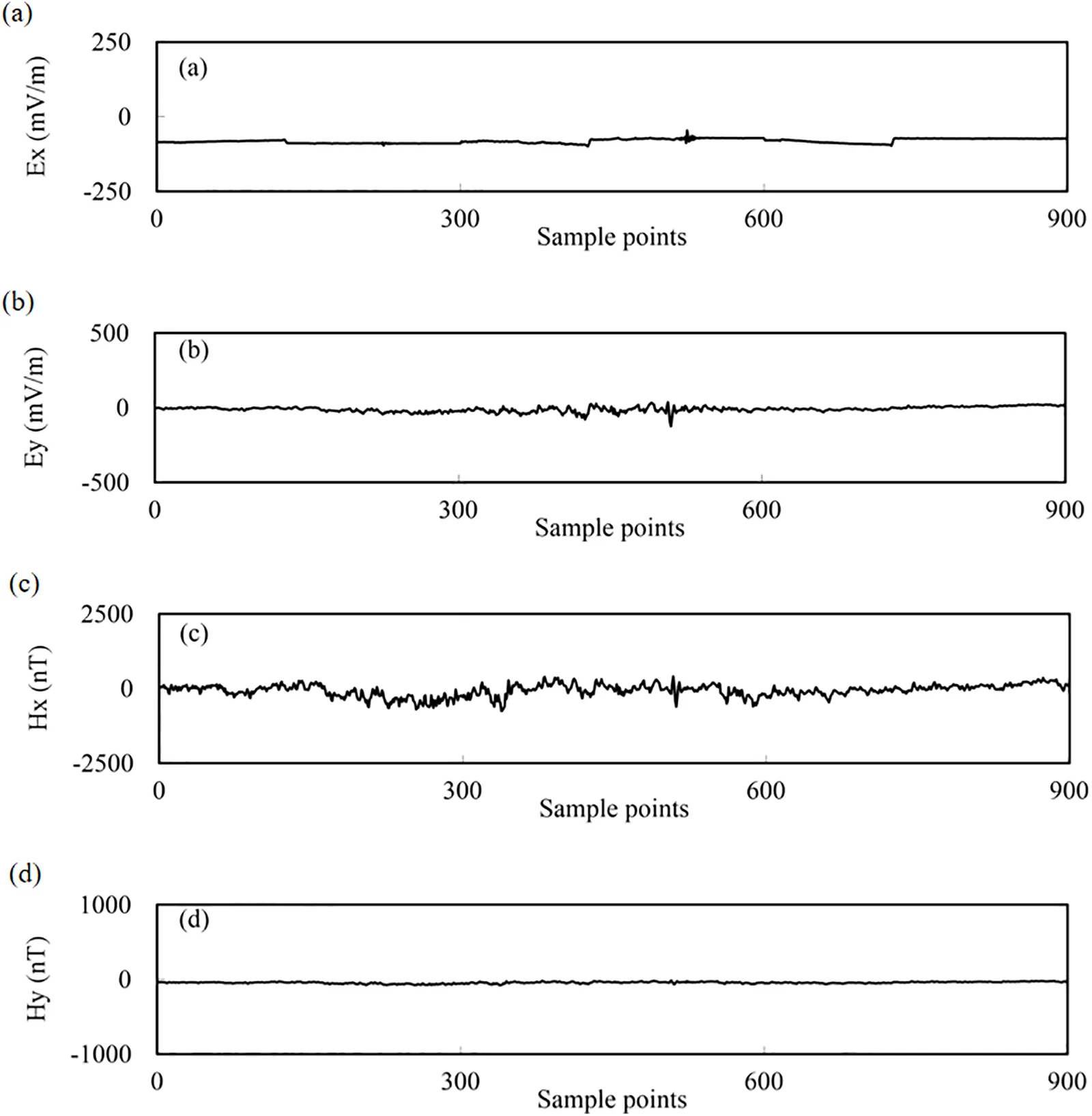
Multichannel MT time series after denoising using the proposed method at Site 48. (a) Ex, (b) Ey, (c) Hx, and (d) Hy. The four components are displayed using channel-specific vertical scales because of their different physical units and amplitude ranges.

Fig 15 shows the apparent resistivity and phase curves for Site 49 before and after processing with different methods. Due to the high noise level at this station, many data points are affected by noise, resulting in significant and scattered frequency point jumps in the apparent resistivity and phase curves within the 1–0.001 Hz frequency band before denoising. As shown in Fig 15b and 15c, traditional SVD and CEEMD provide limited improvements to the apparent resistivity curves; in fact, they even introduce more severe fluctuations in the phase curves. The Robust method (Fig 15d) only partially improves the fluctuations of some jump points, but the frequency points remain discrete and fail to form a continuous, smooth curve, indicating relatively weak improvement in phase curve jump points. In contrast, the proposed method (Fig 15e) provides a more robust interpretation of the apparent resistivity and phase curves, making them smoother. However, there are still a few jump points in the phase curve (*p*_*yx*_) between 0.01 and 0.001 Hz. Overall, the proposed method achieves better performance in smoothing the apparent resistivity and phase curves, enhancing the interpretability and robustness.

**Fig 15:**
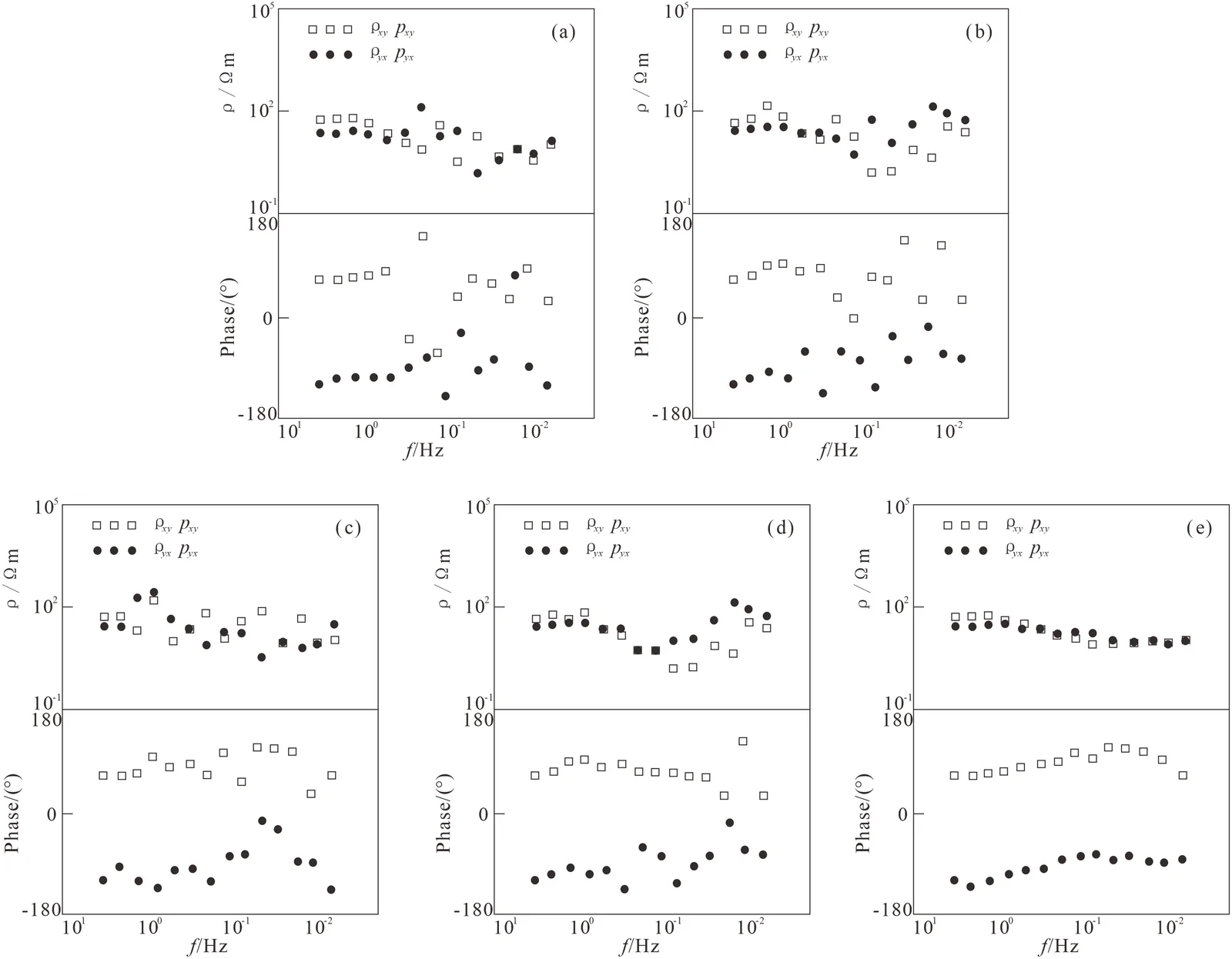
Comparison of apparent resistivity and phase curves for Site 49 before and after denoising using different methods. The apparent resistivity and phase curves of the raw data; (b) results processed by the traditional SVD method; (c) results processed by the CEEMD method; (d) results processed by the Robust method; and (e) results processed by the proposed method.

The apparent resistivity and phase curves for Site 48 before and after processing with the different methods are shown in Fig 16. Since the data quality at Site 48 is relatively high and less affected by noise, the analysis of Fig 16 reveals that the trends of the apparent resistivity and phase curves after processing with the Robust method and the proposed method are highly consistent. Regarding the baseline methods, traditional SVD yields marginal improvements to the jump points of the phase curves around 0.1 Hz, while CEEMD only corrects a few jump points in the apparent resistivity and phase curves. Comparing the *p*_*yx*_ curve processed by the Robust method with that of the proposed method, the latter contains fewer discrete points and exhibits reduced jump amplitudes. In summary, while the Robust and proposed methods yield similar overall trends for this high-quality dataset, the proposed method provides a smoother phase curve with fewer discrete points and lower amplitude jumps.

**Fig 16:**
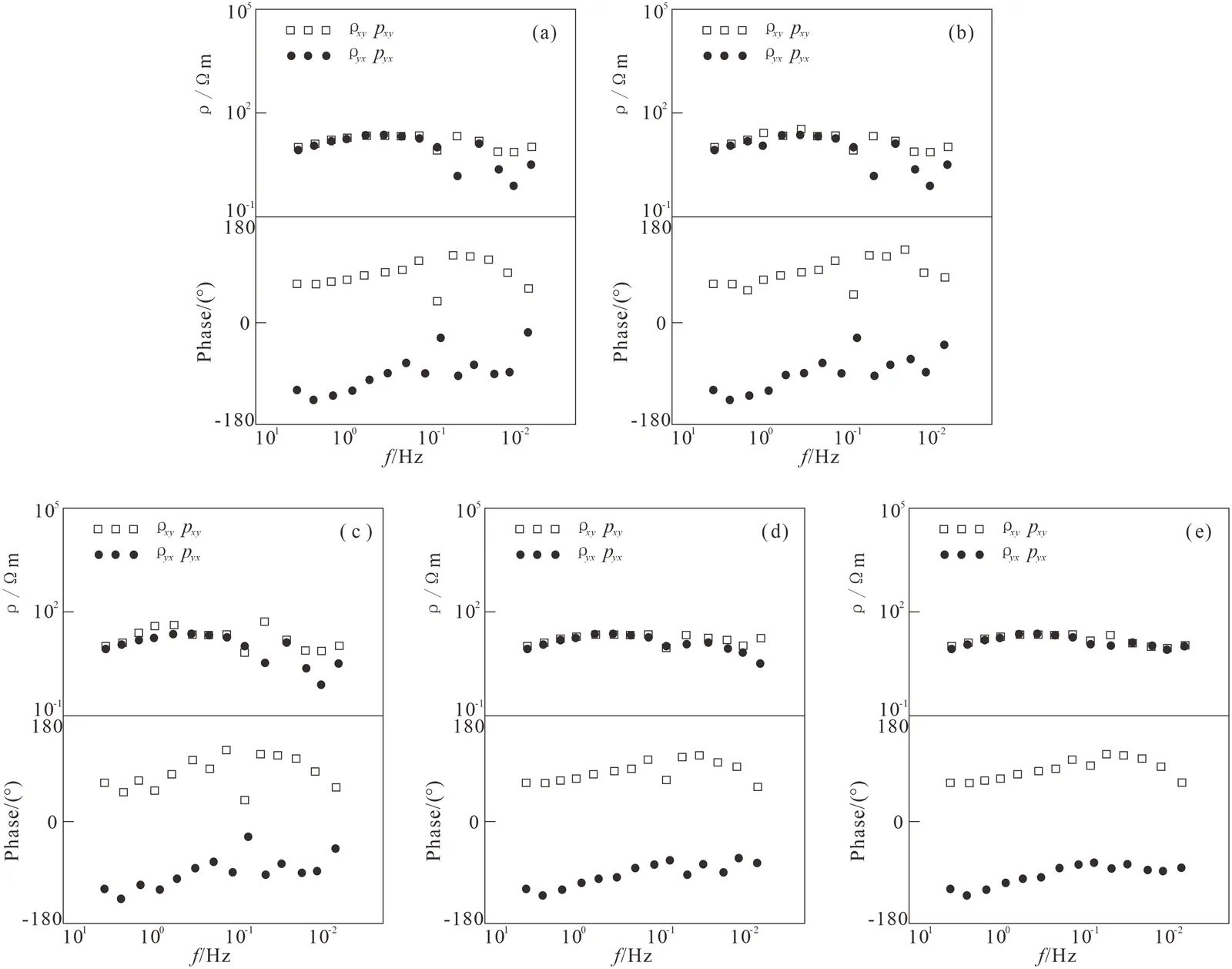
Comparison of apparent resistivity and phase curves for Site 48 before and after denoising using different methods. The apparent resistivity and phase curves of the raw data; (b) results processed by the traditional SVD method; (c) results processed by the CEEMD method; (d) results processed by the Robust method; and (e) results processed by the proposed method.

## 5. Conclusion

This study proposes an improved magnetotelluric (MT) signal-to-noise separation method that integrates Singular Value Decomposition (SVD) with Complementary Ensemble Empirical Mode Decomposition (CEEMD) to address the limitations of traditional SVD in isolating residual noise components. The performance of the proposed method was evaluated through both simulation experiments and MT field data processing, yielding the following conclusions:

In simulation experiments, the proposed method outperformed both the traditional SVD and CEEMD methods across key evaluation metrics, including signal-to-noise ratio (SNR), data error (E), normalized cross-correlation (NCC), and mean squared error (MSE). Notably, the reduction in time-series error values corresponded to frequency spectrum improvements of up to two orders of magnitude. Furthermore, the method achieved more thorough signal-to-noise separation for both large-scale and impulsive noise types, preserving the original signal's morphology and ensuring high spectral consistency with the clean signal.

In field data processing, when applied to heavily contaminated stations (e.g., in the Qilian region), the method demonstrated robust field applicability. It effectively suppressed high-energy noise that had artificially increased the apparent resistivity amplitudes by approximately one order of magnitude. Consequently, the method restored continuous and smooth Cagniard apparent resistivity-phase curves. It was highly effective in correcting severe phase curve distortions (e.g., discontinuities in the pyx curve) in the low-frequency bands, specifically between 1 and 0.001 Hz. Compared to traditional robust methods that left residual discrete outliers, the proposed approach yielded a significantly more reliable interpretation.

Regarding the limitations and future development of the proposed method, similar to other current denoising approaches, slight attenuation of the underlying signals may still occur after processing. In addition, large-scale noise and impulsive noise do not always co-occur in real MT data; therefore, applying the complete two-stage processing procedure to all data segments may occasionally lead to over-processing. Future work will focus on adaptive workflow control, including noise-type identification, parameter selection, and the choice between single-stage and two-stage processing. To provide auxiliary decision information prior to signal reconstruction, two complementary approaches will be investigated. Lightweight statistical or feature-based classification methods will be considered because of their computational efficiency, interpretability, and limited reliance on prior training data. Meanwhile, recent studies have demonstrated the potential of deep learning models, including CNN and CNN-LSTM architectures, for MT noise identification and suppression [41–43]. These approaches will be evaluated in terms of identification accuracy, signal-quality assessment, computational cost, interpretability, and training-data requirements to determine their suitability for adaptive processing-strategy selection within the proposed SVD-CEEMD framework.

Furthermore, a broader evaluation framework would help to better contextualize the performance and applicability of the proposed method. The present study focused on comparisons with traditional SVD, standalone CEEMD, and the Robust method, which are directly related to the motivation and workflow of the proposed approach. Future research will extend the benchmark evaluation to include other representative denoising methods, such as wavelet transform, Hilbert–Huang transform, and variational mode decomposition, under different noise types and field conditions. Longer continuous and synchronized multichannel MT records will also be analyzed to evaluate the preservation of electric–magnetic relationships after denoising. Accordingly, evaluation criteria such as SNR improvement, MSE, runtime, parameter sensitivity, practical applicability, and frequency-dependent inter-channel coherence will be considered where appropriate to provide a more systematic assessment of MT denoising strategies.

## Supporting information

S1 Appendix Sensitivity analysis of the CEEMD ensemble number. (DOCX)
