# Supplementary material for: Research on magnetotelluric signal noise separation based on improved SVD
Source: PLoS One. 2026 Nov 5;21(11):e0334468. doi: 10.1371/journal.pone.0334468 (PMC13739181; doi:10.1371/journal.pone.0334468)
Supplement: Supplementary file 1 [file pone.0334468.s001.docx]

**S1 Appendix. Sensitivity analysis of the CEEMD ensemble number**

To evaluate the sensitivity of the proposed method to the CEEMD ensemble number *I*, the same synthetic mixed-noise signal and signal-reconstruction criterion as those used in the simulation experiment were adopted. Four representative ensemble numbers (*I* = 20, 100, 200, and 500) were evaluated under input SNRs of 0 dB and −3 dB. During the sensitivity analysis, the standard deviation of the noise added by CEEMD was fixed at 0.2 times that of the noisy input signal, and all parameters other than *I* were held constant. Here, *I* denotes the number of Gaussian white-noise realizations used in the CEEMD ensemble. For each parameter combination, the CEEMD decomposition was repeated three times using independent realizations of the added noise while retaining the same input noisy signal. Output SNR, mean squared error (MSE), normalized cross-correlation (NCC), and computational runtime were used as evaluation metrics. The markers in Fig. A1 indicate the mean values of the three runs, and the error bars indicate ±1 standard deviation (SD).

As shown in Fig. A1, increasing *I* from 20 to 100 generally improves the denoising performance under both tested input SNR levels, as indicated by the increase in output SNR, decrease in MSE, and improvement in NCC. When *I* is further increased to 200 or 500, only relatively small variations are observed, and no consistent additional improvement is obtained across all metrics and noise levels. This indicates that the denoising performance tends to stabilize at approximately *I* = 100. In contrast, the computational runtime increases continuously with *I*, with a particularly substantial increase at *I*= 500.

Therefore, considering both denoising performance and computational efficiency, *I* = 100 was considered a reasonable performance–cost compromise among the tested ensemble numbers and was adopted in the proposed method.

Fig. A1. Sensitivity analysis of the CEEMD ensemble number under input SNRs of 0 dB and −3 dB. (a) Output SNR, (b) mean squared error (MSE), (c) normalized cross-correlation (NCC), and (d) computational runtime. Each parameter combination was evaluated using three independent CEEMD runs. Markers indicate the mean values, and error bars indicate ±1 standard deviation (SD).
