# Supplementary material for: Research on magnetotelluric signal noise separation based on improved SVD
Source: PLoS One. 2026 Nov 5;21(11):e0334468. doi: 10.1371/journal.pone.0334468 (PMC13739181; doi:10.1371/journal.pone.0334468)
Supplement: Supplementary file 2 [file pone.0334468.s003.docx]

**Manuscript ID: PONE-D-25-11846**

**Manuscript Title: Research on magnetotelluric signal noise separation based on improved SVD Decomposition**

Dear Dr Jason Morgan:

We are so appreciated for your letter on our manuscript (ID: PONE-D-25-11846) entitled “Research on magnetotelluric signal noise separation based on improved SVD Decomposition”. We are also extremely grateful to the reviewer’s comments on our manuscript and carefully considered every comment, and made cautious revision accordingly. For your and the reviewer’s better check of the revised manuscript, you can find every change in red in our revised manuscript. Based on their suggestions, we have answered the questions in detail one by one.

We sincerely hope our revisions rise to your expectation. If you have any other questions about this manuscript, I would quite appreciate it if you could let me know them in the earliest possible time.

Most sincerely,

Xingtong Yue

Aug 01, 2025

**Reviewer # 1**

The paper by Yue et al. addresses a critical challenge in magnetotelluric (MT) data processing effective suppression of human-induced electromagnetic noise, which increasingly hampers accurate impedance estimation. The authors propose an enhanced signal-to-noise separation method by integrating Singular Value Decomposition (SVD) with Complementary Ensemble Empirical Mode Decomposition (CEEMD). This hybrid approach leverages the amplitude contrast between weak natural MT signals and dominant anthropogenic noise, aiming to overcome the limitations of traditional SVD methods that often leave residual noise artifacts. By demonstrating improved performance on both synthetic and real datasets, the study contributes to a potentially valuable tool for enhancing the reliability of MT interpretations in noisy environments. I appreciate the great piece of work by the authors in this study. I am happy to accept this manuscript for publication after addressing some of my concern on this article.

**Dear Reviewer #1:**

Thank you very much for all of your insightful comments related to the paper. We are grateful to your recognition of our work. We found your feedback very constructive and tried to be responsive to your concerns. Please fine the revised parts through the manuscript in red highlight. We hope to obtain your approval for publication.

Most sincerely,

Xingtong YUE

The follows are my responses to your suggestions and comments.

**Comments:**

**[1] Line 11, SVD is first time appeared here, it is recommended to write full form of it and put short form in brackets.**

**Response:** Your suggestion is very valid. The full form of SVD should indeed be explained when it first appears in the manuscript to make it easier for readers to understand. The revised parts in the manuscript are marked in red, and the specific content is as follows:

“we propose a magnetotelluric signal-to-noise separation method based on improved Singular Value Decomposition (SVD).”

[2] **Line 16, same as previous comment for CEEMD.**

**Response:** Thank you for your valuable comments. We have added the full explanations of the method names in the manuscript. The revised parts in the manuscript are marked in red, and the specific content is as follows:

“Therefore, by combining the Complementary Ensemble Empirical Mode Decomposition (CEEMD) to improve SVD decomposition.”

[3] **This study is focused in Gansu region, Qilian region, but I did not see anything about this region in the introduction part. It is recommended to put at least a paragraph about the study region and show the study area in map view.**

**Response:** Thank you for your valuable comments. In the previous manuscript, the focus was on proposing a data processing method for magnetotelluric (MT) sounding rather than interpreting the subsurface structure of the study area. As a result, we only briefly mentioned the location of the study area without elaborating on it. In the revised manuscript, we have displayed the MT field measurement data on a map and added more information about the study area to enhance the logic of the manuscript. The revised parts in the revised manuscript are marked in red. The adjustment of figure numbers in the subsequent content due to the addition of this image has been marked in red. The specific content is as follows:

“Validation through synthetic experiments (MT data in Gansu area) and field datasets (measured data in Qilian area) confirms that this approach optimally mitigates noise morphology, producing continuous and smooth Cagniard apparent resistivity-phase curves. Gansu area is vast and sparsely populated, and the quality of MT data is high. Thus, adding random noise to MT data in Gansu area can confirm the reliability of the proposed method. Certain measured stations in the Qilian area have destroyed by serious industrialization, with poor data quality. Thus, the effectiveness of the proposed method has been confirmed through processing the above measured data. The location of survey areas is shown in Figure 1.”

Fig 1 The location of survey areas

**[4] Line 183-185, you mentioned the signal shown in Figure 2 is synthetic noisy signal and later in the sentence, you mentioned the signal is obtained from Gansu province. Please make it consistent.**

**Response:** I apologize for the confusion and misunderstanding caused in the previous manuscript. The data shown in Figure 2 are indeed synthetic data, but the basis for these synthetic data is derived from real magnetotelluric (MT) measurement data obtained in Gansu Province. Given that MT signals are natural and have a broadband characteristic, it is very difficult to simulate them accurately. Therefore, we used the “clean” MT measurement data from Gansu Province as the basis for our synthetic data. We have rephrased and unified these statements in the revised manuscript to avoid any further misunderstandings. The revised parts in the manuscript are marked in red. The specific content is as follows (due to the increase in the number of figures, Figure 2 in the original text has become Figure 3 in the revised manuscript):

“Figure 3 illustrates the process of generating synthetic noisy signals. Clean MT measured data (collected in Gansu Area, with sampling rate at 15 Hz) serves as the basis for the synthesis process, and different noises are added to generate noisy signals. The sparse population in Gansu Area results in minimal anthropogenic noise, ensuring high-quality MT data acquisition. As observed in Figure 3a, the original signal is stable without any noise waveform.”

**[5] Line 186-195, Most of the contents in this section is the same as caption provided in Figure. It is better to explain results rather than just repeating captions. Please follow this recommendation throughout the writeup**

**Response:** Thank you for your valuable comments. In the revised manuscript, we have added content that explains the results, instead of merely repeating the figure captions. The revised parts are marked in red, and the corresponding content is as follows:

“Figures 3d to 3f depict the noisy signals with different types of noise added, such as square niose、impluse noise and mixed noise. It can be seen from Figures 3d to 3f that the morphology of the original MT signal is almost completely obscured, making the time series no longer continuous and smooth. Meanwhile, the amplitude of the original signal was changed (the effective signal energy being much lower than the noise energy), making it difficult to obtain accurate impedance results.”

“Figure 4 shows the corresponding spectra of the original signal and noisy signals in Figure 3. By comparing Figure 4a with Figures 4b and 4d, it is evident that the addition of large-scale noise not only increases the amplitude of the main frequency peak of the effective signal but also introduces multiple side peaks and bandwidths around the main peak, with the amplitude of these side peaks decreasing progressively. This leads to a bias in impedance estimation. From Figures 4a and 4c, it can be seen that impulse noise does not cause a sudden increase in the spectral amplitude but continuously interferes with the entire spectrum.”

**[6] Line 206, 15Hz correct this as 15 Hz**

**Response:** We apologize for this error. We have checked the entire text for this mistake and made the necessary corrections. The revised parts are marked in red.

**[7] Figures 8b and 8c, why are some points out of the plot?**

**Response:** In the previous manuscript, in an effort to make the differences in the processing results of different methods clearer to the readers, we overlooked the fact that some points exceeded the coordinate range. In the revised manuscript, we have modified the relevant figures. The specific content is as follows (due to the increase in the number of figures, Fig. 8 in the original text has become Fig. 9 in the revised manuscript):

Fig.9 The apparent resistivity phase curves calculated by the original signal and denoised signal

(a) The apparent resistivity phase curves of the original data; (b) The apparent resistivity phase curves of the noisy signal; (c) The apparent resistivity phase curves of the denoised signal after traditional SVD decomposition; (d) The apparent resistivity phase curves of the denoised signal obtained by CEEMD; (e) The apparent resistivity phase curves of the denoised signal processed by the proposed method.

**[8] Line 303, it is highly recommended to put station map and show the details around the stations in map. This helps the reader to follow what you want to say. In addition to the station location, if you can also plot the location of industry and population density in map, this will be helpful to readers to follow your article.**

**Response:** Thank you for your valuable comments. In the revised manuscript, we have added a figure showing the locations of the measurement stations, including Site 49, Site 48, and the industrial area. However, we apologize that we were unable to find specific information regarding population density. The revised parts are marked in red. The specific content is as follows:

Figure 10. The locations of MT measured stations

**Thank you again for your time and efforts to review our paper.**

**Dear Reviewer #2:**

Thank you very much for your comments. We have made revisions in response to each of your suggestions. We have added links to the data that can be accessed and included more appropriate references. The revised parts are marked in red in the revised manuscript. Below are the main corrections in the manuscript and our responses to your comments. We hope to obtain your approval for publication.

Most sincerely,

Xingtong YUE

The follows are my responses to your suggestions and comments.

**Reviewer # 2**

I have found the work well written and clear.

**Comments:**

**[1] I couldn't find a link to the data set as declared in the submitting form**

Response: Your suggestion is very valid. We have added the data source information at the end of the manuscript. The revised parts in the revised manuscript are marked in red. The specific content is as follows:

**Supporting information**

We provide the measured magnetotelluric data of site48 and site49. The data were collected by Phoenix V5-2000 and processed by SSMT2000.

Site48.tbl and Site49.tbl are the calibration files for the two sites.

Site48.ts3, Site48.ts4 and Site48.ts5 are the high-frequency, medium-frequency and low-frequency magnetotelluric data of site48.

Site49.ts3, Site49.ts4 and Site49.ts5 are the high-frequency, medium-frequency and low-frequency magnetotelluric data of site49.

For easier access and visualization, the original data were converted from their proprietary format into Microsoft Excel.

The high-frequency, medium-frequency and low-frequency magnetotelluric time series data of Site 48 (files Site48.TS3, Site48.TS4, and Site48.TS5) are merged and converted into a single Excel file (Site48.xlsx) , containing the time-domain data of the Site 48. Similarly, the time series data for Site 49 were processed and compiled into a single file (Site49.xlsx).

The data can be downloaded from <https://doi.org/10.6084/m9.figshare.30031372.v3>

**[2] References to other impedance estimation methods
It seems to me that some relevant works on this subject have not been cited/briefly discussed:**

Eisel M, Egbert GD (2001) On the stability of magnetotelluric transfer function estimates and the reliability of their variances. Geophysical Journal International, Volume 144, Issue 1, January 2001, Pages 65–82, https://doi.org/10.1046/j.1365-246x.2001.00292.x

Rizzello, D., Armadillo, E., Smooth magnetotelluric impedance estimation by optimization, Journal of Applied Geophysics, Volume 210, 2023, 104952, ISSN 0926-9851, https://doi.org/10.1016/j.jappgeo.2023.104952.

Jin Li, Shanshan Liu, Yiqun Peng, Jingtian Tang, Xian Zhang , Yong Li. A method for magnetotelluric data processing based on sparsity adaptive stage-wise orthogonal matching pursuit. Journal of Applied Geophysics, Volume 198, March 2022, 104577. https://doi.org/10.1016/j.jappgeo.2022.104577

Qining ZhanQining ZhanCai LiuCai LiuYang LiuYang LiuPengfei Zhao Pengfei Zhao. Data processing method for magnetotelluric sounding based on cepstral analysis. Front. Earth Sci., 18 October 2023, Sec. Geomagnetism and Paleomagnetism, Volume 11 - 2023 | https://doi.org/10.3389/feart.2023.1183188

Gary D. Egbert. Robust multiple-station magnetotelluric data processing. Geophysical Journal International, Volume 130, Issue 2, August 1997, Pages 475–496, https://doi.org/10.1111/j.1365-246X.1997.tb05663.x

**As general reference, you should also consider:**
Egbert, G. (2021). Magnetotelluric Data Processing. In: Gupta, H.K. (eds) Encyclopedia of Solid Earth Geophysics. Encyclopedia of Earth Sciences Series. Springer, Cham. <https://doi.org/10.1007/978-3-030-58631-7_128>

Response: Thank you for your valuable suggestions. We have added some of the references you listed to the manuscript. The revised parts in the revised manuscript are marked in red.

“Eisel M, Egbert GD. On the stability of magnetotelluric transfer function estimates and the reliability of their variances. Geophysical Journal International, 2001, 144(1): 65–82.

Rizzello D, Armadillo E, Smooth magnetotelluric impedance estimation by optimization, Journal of Applied Geophysics, 2023, 210:104952.

Li J, Liu S, Peng Y et al. A method for magnetotelluric data processing based on sparsity adaptive stage-wise orthogonal matching pursuit. Journal of Applied Geophysics, 2022, 198:104577.

Egbert, G. Magnetotelluric Data Processing. In: Gupta, H.K. (eds) Encyclopedia of Solid Earth Geophysics. Encyclopedia of Earth Sciences Series. Springer, Cham, 2021.”

**[3] Impedance estimation by SVD has been discussed extensively by Egbert and collaborators in the following works, which have not been discussed. A reference to these works and a brief description of their approach should be added, also specifying what has been improved**

Egbert, G.D., Booker, J.R. Multivariate analysis of geomagnetic array data: 1. The response space.

https://agupubs.onlinelibrary.wiley.com/doi/10.1029/JB094iB10p14227

Egbert, G.D. Multivariate analysis of geomagnetic array data: 2. Random source models. https://agupubs.onlinelibrary.wiley.com/doi/abs/10.1029/JB094iB10p14249

Egbert, G.D.. Robust multiple-station magnetotelluric data processing. Geophysical Journal International, Volume 130, Issue 2, August 1997, Pages 475–496, https://doi.org/10.1111/j.1365-246X.1997.tb05663.x

Egbert, G.D. Processing And Interpretation Of Electromagnetic Induction Array Data. Surveys in Geophysics 23, 207–249 (2002). https://doi.org/10.1023/A:1015012821040

Response: Thank you for your valuable comments. We have incorporated additional references and provided brief descriptions in our manuscript. The specific content is as follows:

Egbert (1997) proposed a multivariate RMEV estimator framework that optimizes MT impedance estimation through automated incoherent-noise diagnosis and robust multichannel signal coherence extraction.

Egbert (2002) further exploited the utilization of the array data to reduce bias, improve signal-to-noise ratios, and provide better control over coherent noise in estimates of MT impedances.

**Thank you again for your time and efforts to review our paper.**
