# Supplementary material for: Research on magnetotelluric signal noise separation based on improved SVD
Source: PLoS One. 2026 Nov 5;21(11):e0334468. doi: 10.1371/journal.pone.0334468 (PMC13739181; doi:10.1371/journal.pone.0334468)
Supplement: Supplementary file 3 [file pone.0334468.s004.docx]

**Manuscript ID: PONE-D-25-11846**

**Manuscript Title: Research on magnetotelluric signal noise separation based on improved SVD Decomposition**

Dear Dr Brian Patrick Weaver:

We greatly appreciate your letter regarding our manuscript (ID: PONE-D-25-11846) entitled “Research on magnetotelluric signal noise separation based on improved SVD Decomposition”. We are also extremely grateful for the reviewers’ constructive comments. We have carefully considered every comment and made thorough revisions accordingly.

To facilitate the evaluation process for you and the reviewers, all modifications have been highlighted in red in the revised manuscript. Furthermore, based on their valuable suggestions, we have provided detailed, point-by-point responses to each question in the accompanying document.

We sincerely hope that our revisions meet your expectations. Should you have any further questions regarding this manuscript, please do not hesitate to contact us at your earliest convenience.

Most sincerely,

Fuming Qu

Jun 13, 2026

**Dear Reviewers #1, #2, and #3:**

We would like to express our sincere gratitude to you for your time and effort in evaluating our manuscript. We are deeply honored by your positive assessments and for recommending our work for publication. Your recognition of the significance and value of our research is highly encouraging to our entire team. Thank you for your support.

Most sincerely,

Fuming Qu

**Reviewer # 4**

**The manuscript proposes an improved SVD-based noise separation method to address the problem of residual anthropogenic noise in magnetotelluric (MT) signal denoising using traditional singular value decomposition (SVD). The topic is meaningful, and the manuscript has been well revised according to previous review comments. However, several details still need further improvement.**

**Dear Reviewer #4:**

Thank you very much for your insightful comments and suggestions regarding our manuscript. We are deeply grateful for your recognition of our work. We found your feedback to be highly constructive, and we have done our best to fully address your remaining concerns. Please find the revised parts throughout the manuscript highlighted in red. We hope these revisions meet with your approval for publication.

Most sincerely,

Fuming Qu

The following are our point-by-point responses to your comments:

**Comments:**

**[1] The revised introduction is generally acceptable. However, since this paper mainly proposes an improved SVD method that combines SVD with CEEMD, the authors should further clarify in the introduction the advantages and improvements of the proposed method compared with existing studies (e.g., doi.org/10.1190/geo2024-0066.1, etc.), both in theoretical and application aspects. This would help to better highlight the innovation and necessity of the proposed approach compared with other improved methods, such as K-SVD and ICEEMDAN**

**Response:** Thank you for your valuable and insightful comments. We fully agree that clarifying the advantages of the proposed method compared to existing advanced studies is crucial for highlighting its innovation and necessity.

In the revised manuscript, we have expanded the introduction to explicitly discuss the advantages of our SVD-CEEMD approach over methods like deep learning (e.g., CS-ResNet; doi.org/10.1190/geo2024-0066.1), K-SVD, and ICEEMDAN. Specifically, we summarized its theoretical advantage as a data-driven, unsupervised approach free from massive prior data dependencies, and its practical advantage as a two-stage mechanism that effectively resolves boundary artifacts and edge suppression issues in severe interference environments.

The revised parts in the manuscript are marked in red, and the references have been updated accordingly. The specific added content is as follows:

Despite powerful noise suppression capabilities, emerging deep learning frameworks (e.g., CS-ResNet [32]) demand massive, uncontaminated training datasets and high computational loads, severely limiting their field applicability in high-interference mining zones. Meanwhile, dictionary learning methods like K-SVD [33] rely heavily on pre-trained dictionaries, struggling to efficiently adapt to unpredictable, non-stationary impulsive noises. Additionally, while advanced mode decomposition algorithms like ICEEMDAN [4,34] effectively mitigate mode mixing, they fail to independently resolve the high-energy boundary mutations characteristic of large-scale noise in severe interference environments.

To overcome these limitations, this study proposes a data-driven, unsupervised hybrid method combining SVD with complementary ensemble empirical mode decomposition (CEEMD). Theoretically, it circumvents the high computational costs and prior data dependencies inherent in dictionary and deep learning methods. Practically, it implements an efficient two-stage mechanism: SVD primarily isolates high-energy noise based on amplitude discrepancies, while CEEMD secondarily eliminates the residual impulsive noise. This joint strategy resolves both the boundary artifacts of traditional SVD and the edge suppression issues of standalone CEEMD, yielding superior signal-to-noise separation and computational efficiency.

[2] **The formatting of some equations is inconsistent. For instance, ellipses are missing in some expressions, and equation numbers should be uniformly right-aligned. It is recommended to thoroughly check and unify the equation formatting throughout the manuscript.**

**Response:** We sincerely apologize for the formatting inconsistencies in our initial submission, and we greatly appreciate your review. Your attention to detail has significantly improved the professional presentation and readability of our manuscript.

Per your recommendation, we have conducted a thorough and comprehensive review of all mathematical equations, inline variables, and symbols throughout the text. Specifically, we have implemented the following formatting unifications:

(1) We have reformatted all standalone equations using the standard equation editor to ensure that every equation number is uniformly right-aligned.

(2) We have added standard mathematical ellipses where they were missing in sequence definitions (e.g., Equation 2) to strictly adhere to mathematical typesetting standards.

We have unified the equation formatting throughout the entire manuscript. All modified equations and mathematical expressions have been highlighted in red in the revised manuscript.

$$\begin{aligned} \text{Y}\text{=}\text{X}\text{+}\text{N}\text{=}\left[ \text{U}_{\text{s}}\text{…}\text{U}_{\text{n}} \right]\left[ \begin{matrix} \text{S}_{\text{s}} & \text{⋯} & \text{0} \\ \text{⋮} & \text{⋱} & \text{⋮} \\ \text{0} & \text{⋯} & \text{S}_{\text{n}} \end{matrix} \right]\left[ \begin{matrix} \text{V}_{\text{s}}^{\text{T}} \\ \text{⋮} \\ \text{V}_{\text{n}}^{\text{T}} \end{matrix} \right]\#\text{(2)} \end{aligned}$$

[3] **Some symbols are not rigorously defined. For example, it is unclear whether − and + in Equations (4) and (5) refer to the same parameter. Please clearly define all symbols and ensure the correct use of italic and upright fonts for variables and constants to meet academic standards.**

**Response:** Thank you very much for this careful and valuable suggestion. We completely agree that rigorous symbol definitions and correct mathematical formatting are crucial for academic clarity.

To address the ambiguity regarding the plus/minus signs in the former Equations (4) and (5), we have consolidated them into a single, more concise equation, which inherently avoids the previous sign confusion while fully preserving the underlying logic. A detailed explanation has been supplemented right below the new equation.

Furthermore, we have conducted a thorough review of the entire manuscript to ensure that all symbols are rigorously and clearly defined. We have also strictly unified the use of italic fonts for mathematical variables and upright fonts for constants, operators, and descriptive subscripts in accordance with academic publishing standards. All the modified equations and text have been highlighted in red in the revised manuscript.

The revised parts in the manuscript are marked in red, and the specific added content is as follows:

$$\begin{aligned} \text{x}^{\left( \text{i} \right)}\text{=}\text{x}\text{+}\text{ε}_{\text{1}}\text{E}_{\text{1}}\left( \text{w}^{\left( \text{i} \right)} \right)\#\text{(4)} \end{aligned}$$

In which, *w*^(^*^i^*^)^ represents the *i*^th^ group of Gaussian white noise, where *i=1,2,3,…,I,* and *E*_1_(⋅) denotes the first-order component produced by EMD decomposition. *ε*_1_ represents the weight coefficient of the noise component *E*_1_(*w*^(^*^i^*^)^) (its value is defined as the ratio of the signal-to-noise ratio of the added noise to the standard deviation of this noise component). Based on equation (4), the sequence *x*^(^*^i^*^)^ is obtained to derive the first-order IMF component.

**[4] In the simulation experiments, readers expect to see quantitative metrics rather than only graphical comparisons. It is suggested to add corresponding numerical values above the rectangular regions in Figures 7 and 8 to enhance the persuasiveness of the result.**

**Response:** Thank you for your constructive suggestion. We fully agree that providing quantitative metrics is essential for intuitively and objectively demonstrating the effectiveness of the denoising methods.

We have added the corresponding numerical evaluation metrics directly above the rectangular regions in both Figure 7 and Figure 8. Furthermore, to provide a more comprehensive analysis, we have expanded the discussion in the revised manuscript.

The revised parts in the manuscript are marked in red, and the specific added content is as follows:

Figure 8 presents the evaluation of the spectral properties of the denoised signals using different methods, corresponding to Figure 7. To quantitatively evaluate the denoising performance, the numerical metrics of the different methods under various noise conditions have been annotated in Figures 7 and 8. These specific values provide a direct and objective comparison of the improvements achieved by each method.

Figure 7. Evaluation of denoised signals after suppressing different noises.

(a), (b), (c), and (d) represent the signal-to-noise ratio (SNR), data error (E), normalized cross-correlation (NCC), and mean square error (MSE) of the data processed by different methods, respectively. The black solid line represents the traditional SVD method, the white dashed line represents the CEEMD method, and the red solid line represents the proposed method. The numerical values above the lines indicate the specific quantitative metrics obtained by each method.

Figure 8. Evaluation of spectral properties before and after denoising.

(a), (b), (c), and (d) correspond to the evaluation metrics shown in Figure 7, respectively. The black solid line represents the traditional SVD method, the white dashed line represents the CEEMD method, and the red solid line represents the proposed method. The numerical values above the lines indicate the specific quantitative metrics obtained by each method.

**[5] The earlier sections mainly compare the traditional SVD, CEEMD, and the proposed method, but Figures 14 and 15 only present comparisons with the Robust method. It is recommended to include the results of traditional SVD and CEEMD to more comprehensively demonstrate the advantages of the improved method.**

**Response:** We sincerely appreciate your constructive suggestion. We completely agree that including the results of traditional SVD and CEEMD in the field data analysis provides a much more comprehensive and convincing demonstration of the proposed method's advantages.

We have updated Figures 14 and 15 to include the processing results of the traditional SVD method (Subplot b) and the CEEMD method (Subplot c), alongside the raw data (a), the Robust method (d), and our proposed method (e).

Correspondingly, we have expanded the discussion in the revised manuscript to systematically compare the performance of all these methods. Specifically, we detailed how traditional SVD and CEEMD provide limited improvements and can even introduce severe fluctuations in the phase curves for highly contaminated data (Site 49), whereas the proposed method effectively smooths the curves and enhances interpretability. For the high-quality dataset (Site 48), we also added discussions on the marginal improvements yielded by the baseline methods compared to the smoother results of the proposed method.

The updated figures, captions, and corresponding descriptive text have been highlighted in red in the revised manuscript, and the specific added content is as follows:

Figure 14 shows the apparent resistivity phase curves for Site 49 before and after processing with different methods. Due to the high noise level at this station, many data points are affected by noise, resulting in significant and scattered frequency point jumps in the apparent resistivity-phase curves within the 1-0.001 Hz frequency band before denoising. As shown in Figures 14b and 14c, traditional SVD and CEEMD provide limited improvements to the apparent resistivity curves; in fact, they even introduce more severe fluctuations in the phase curves. The Robust method (Figure 14d) only partially improves the fluctuations of some jump points, but the frequency points remain discrete and fail to form a continuous, smooth curve, indicating relatively weak improvement in phase curve jump points. In contrast, the proposed method (Figure 14e) provides a more robust interpretation of the apparent resistivity-phase curves, making them smoother. However, there are still a few jump points in the phase curve (*p_yx_*) between 0.01 and 0.001 Hz. Overall, the proposed method achieves better performance in smoothing the apparent resistivity-phase curves, enhancing the interpretability and robustness.

The apparent resistivity phase curves for Site 48 before and after processing with the different methods are shown in Figure 15. Since the data quality at Site 48 is relatively high and less affected by noise, the analysis of Figure 15 reveals that the trends of the apparent resistivity-phase curves after processing with the Robust method and the proposed method are highly consistent. Regarding the baseline methods, SVD yields marginal improvements to the jump points of the phase curves around 0.1 Hz, while CEEMD only corrects a few jump points in the apparent resistivity curves. Comparing the *p_yx_* curve processed by the Robust method with that of the proposed method, the latter contains fewer discrete points and exhibits reduced jump amplitudes. In summary, while the Robust and proposed methods yield similar overall trends for this high-quality dataset, the proposed method provides a smoother phase curve with fewer discrete points and lower amplitude jumps.

Figure 14. Comparison of apparent resistivity-phase curves for Site 49 before and after denoising using different methods.

(a) The apparent resistivity-phase curves of the raw data; (b) Results processed by the traditional SVD method; (c) Results processed by the CEEMD method; (d) Results processed by the Robust method; (e) Results processed by the proposed method.

Figure 15. Comparison of apparent resistivity-phase curves for Site 48 before and after denoising using different methods.

(a) The apparent resistivity-phase curves of the raw data; (b) Results processed by the traditional SVD method; (c) Results processed by the CEEMD method; (d) Results processed by the Robust method; (e) Results processed by the proposed method.

**[6] The conclusion section is currently too brief. It is suggested to summarize the algorithm’s performance from both quantitative and qualitative perspectives using simulation and field data, highlighting the improvements of the proposed method in terms of noise suppression effectiveness and signal fidelity.**

**Response:** Thank you for your insightful and constructive suggestion. We fully agree that a comprehensive summary structured from both quantitative and qualitative perspectives is essential to properly reflect the algorithm's performance.

Per your recommendation, we have significantly expanded and completely rewritten the Conclusion section. Specifically, we have comprehensively summarized the method’s performance to highlight the improvements in noise suppression effectiveness and signal fidelity:

(1) Using Simulation Data: From a quantitative perspective, we summarized the algorithm's superiority across key evaluation metrics (SNR, E, NCC, and MSE), noting that reduced time-series errors translated into frequency spectrum improvements of up to two orders of magnitude. From a qualitative perspective, we emphasized the high signal fidelity, demonstrating that the method successfully preserves the original signal's morphology while achieving thorough noise separation.
(2) Using Field Data: Quantitatively, we highlighted the algorithm’s effectiveness in suppressing high-energy noise, correcting artificially increased apparent resistivity amplitudes by approximately one order of magnitude. Qualitatively, we emphasized the restoration of continuous and smooth Cagniard apparent resistivity-phase curves, specifically noting the correction of severe phase distortions (e.g., jump points in the *p*_yx_ curve) in the low-frequency bands (1 to 0.001 Hz).

We believe the revised conclusion now effectively and systematically demonstrates the necessity and superiority of the proposed SVD-CEEMD method. The revised parts in the manuscript are marked in red, and the specific added content is as follows:

**4 Conclusion**

This study proposes an improved magnetotelluric (MT) signal-noise separation method that integrates Singular Value Decomposition (SVD) with Complementary Ensemble Empirical Mode Decomposition (CEEMD) to address the limitations of traditional SVD in completely extracting noise components. The performance of the proposed method was comprehensively evaluated through both simulation experiments and MT field data processing, yielding the following conclusions:

In simulation experiments, the proposed method outperformed both the traditional SVD and CEEMD methods across key evaluation metrics, including signal-to-noise ratio (SNR), data error (E), normalized cross-correlation (NCC), and mean square error (MSE). Notably, the reduction of time-series error values translated into frequency spectrum improvements of up to two orders of magnitude. Furthermore, the method achieved more thorough signal-noise separation for both large-scale and impulsive noises, successfully preserving the original signal's morphology and ensuring high spectral consistency with the effective signal.

In field data processing, when applied to heavily contaminated stations (e.g., in the Qilian region), the method demonstrated robust practical applicability. It effectively suppressed high-energy noise that had artificially increased the apparent resistivity amplitudes by approximately one order of magnitude. Consequently, the method successfully restored continuous and smooth Cagniard apparent resistivity-phase curves. It demonstrated a particular strength in correcting severe phase curve distortions (such as jump points in the *p*_yx_ curve) in the low-frequency bands, specifically between 1 and 0.001 Hz. Compared to traditional robust methods that left residual discrete outliers, the proposed approach provided a significantly more reliable interpretation.

As to limitations and future work, similar to other current denoising methods, the proposed method may still result in some loss of effective signals. Furthermore, since large-scale noise and abrupt noise do not always co-occur in real data, applying this dual-stage method to suppress both types simultaneously can occasionally lead to over-processing issues. In future research, we plan to combine neural networks with the proposed framework to intelligently address these problems and optimize the separation process.

**Thank you again for your time and efforts to review our paper.**
