# Supplementary material for: Research on magnetotelluric signal noise separation based on improved SVD
Source: PLoS One. 2026 Nov 5;21(11):e0334468. doi: 10.1371/journal.pone.0334468 (PMC13739181; doi:10.1371/journal.pone.0334468)
Supplement: Supplementary file 4 [file pone.0334468.s005.docx]

**Manuscript ID: PONE-D-25-11846R3**

**Manuscript Title: Research on magnetotelluric signal noise separation based on improved SVD**

**Dear Dr Feier Chen:**

We greatly appreciate your letter regarding our manuscript (**ID: PONE-D-25-11846R2**) entitled “Research on magnetotelluric signal noise separation based on improved SVD”. We are also extremely grateful for the constructive comments provided by you and the reviewers. We have carefully considered every comment and made thorough revisions accordingly.

To facilitate the evaluation process for you and the reviewers, all modifications have been highlighted in red in the revised manuscript. Furthermore, based on their valuable suggestions, we have provided detailed, point-by-point responses to each question in the accompanying document.

We sincerely hope that our revisions meet your expectations and align with the rigorous academic standards of PLOS ONE. Should you have any further questions regarding this manuscript, please do not hesitate to contact us at your earliest convenience.

Most sincerely,

Fuming Qu

July 3, 2026

**Dear Reviewers #1, #2, #3 and #5:**

We would like to express our sincere gratitude to you for your time, effort, and rigorous evaluation of our manuscript throughout the review process.

We are deeply honored by your positive assessments and are thrilled that you found the integration of SVD and CEEMD to be a valuable contribution to enhancing the reliability of magnetotelluric (MT) interpretations in noisy environments. Your recognition of the significance of our research—particularly its demonstrated effectiveness on both synthetic and real datasets—is highly encouraging to our entire team.

Thank you again for your invaluable support, your previous constructive feedback that helped shape this manuscript, and for recommending our work for publication.

Most sincerely,

Fuming Qu

**Dear Reviewer #4:**

We sincerely thank you for your rigorous evaluation and highly constructive comments regarding our manuscript. We also deeply appreciate your acknowledgement of the potential research significance of our proposed SVD-CEEMD method.

You rightly pointed out that our previous version contained detailed errors, lacked sufficient justification for the experimental design, and required a more rigorous logical presentation and stronger evidentiary support. We took your criticisms very seriously. Guided by your insightful suggestions, we have conducted a comprehensive review and a substantial revision of the entire manuscript.

Specifically, we have meticulously corrected the detailed formatting and language errors, significantly strengthened the justification and theoretical analysis of our experimental design, and rigorously refined our logical presentation to ensure that all conclusions are firmly supported by robust evidence. We believe that your strict and professional feedback has profoundly improved the credibility, persuasiveness, and overall quality of our paper.

To facilitate your review, all modifications have been clearly highlighted in red in the revised manuscript. We genuinely hope that these comprehensive revisions fully address your concerns and meet the high publication standards of the journal.

Most sincerely,

Fuming Qu

The following are our detailed, point-by-point responses to your comments:

**Comments:**

[1] **Abnormal characters, such as square symbols (□), appear in Equations (2) and (13), suggesting possible formula rendering errors, encoding issues, or missing mathematical symbols. The authors are advised to thoroughly check the formatting and encoding of all equations throughout the manuscript to ensure that mathematical expressions are displayed accurately and completely, thereby avoiding potential confusion for readers.**

**Response:** We sincerely thank you for bringing this formatting issue to our attention, and we apologize for any confusion it may have caused during the reading process.

The issue was caused by a system conversion error. Originally, we used the built-in Equation Editor in Microsoft Word to insert these formulas. While all mathematical symbols and equations displayed perfectly in our local Word document, an encoding mismatch occurred when the submission system automatically converted the Word file into a PDF. This conversion failure caused certain mathematical symbols to render as abnormal square characters (□).

To resolve this issue completely, we have meticulously re-entered and reformatted Equations, along with thoroughly checking all other mathematical expressions and variables throughout the manuscript. After uploading the revised manuscript, we rebuilt the PDF in the submission system and repeatedly verified the final generated file. We can now confirm that all formulas, mathematical symbols, and encodings are displayed accurately and completely in the system-generated PDF.

We greatly appreciate your rigorous check and patience regarding this technical issue.

[2] **The manuscript contains numerous spelling and language errors. For example, "Impluse noise" should be corrected to "Impulse noise", and "niose" should be corrected to "noise". These issues are not isolated cases but appear throughout the manuscript, indicating that the language proofreading remains insufficient. The authors are strongly encouraged to conduct a thorough language review and professional editing of the entire manuscript to improve its overall quality, readability, and adherence to academic writing standards.**

**Response:** We sincerely thank you for carefully reading our manuscript and pointing out these spelling and language errors. We apologize for the insufficient proofreading in our initial submission.

Following your suggestion, we have corrected all instances of “Impluse noise” to “Impulse noise” and “niose” to “noise” throughout the text. Beyond these specific corrections, we have taken your advice very seriously and conducted a comprehensive, word-by-word language review of the entire manuscript. Every single sentence has been meticulously checked for grammatical correctness, and the overall language has been extensively polished to improve readability, clarity, and adherence to academic writing standards.

Because these linguistic refinements and spelling corrections are numerous and distributed throughout the entire text, we have not listed every single minor change in this response letter. Instead, all language, spelling, and grammatical modifications have been clearly highlighted in red in the revised manuscript for your convenience.

We sincerely appreciate your rigorous evaluation, and we hope that our extensive proofreading and language improvements now fully meet your expectations and the high standards of the journal.

[3] **Key evaluation metrics such as SNR and MSE are standard abbreviations and, according to common academic writing conventions, should be presented in upright type rather than italics. The authors are also encouraged to ensure consistent formatting throughout the manuscript for variables, functions, and evaluation metrics, thereby maintaining uniformity and clarity in the presentation of mathematical symbols and notations.**

**Response:** We sincerely thank you for this highly professional and meticulous observation.

Following your valuable suggestion, we have corrected the formatting of the evaluation metrics (including SNR, MSE, NCC, and E), changing them all to upright type throughout the manuscript and equations.

Furthermore, we have conducted a comprehensive and systematic review of all mathematical formulas, symbols, and notations across the entire manuscript to ensure strict adherence to standard academic formatting conventions. Specifically, we have standardized the formatting according to the following rules:

(1) Upright Type: We have ensured that all standard abbreviations (e.g., SNR, MSE, SVD, CEEMD, IMF), established evaluation metrics, standard mathematical operators/functions (e.g., max, min, log, and the mean($\cdot$)operator), and descriptive subscripts/superscripts (e.g., the subscript 's' for signal and 'n' for noise in $\text{U}_{\text{s}}$ and $\text{U}_{n}$, or T for transpose) are consistently presented in upright type.
(2) Italics: We have ensured that all physical quantities, continuous and discrete scalar variables (e.g., *x*, *y*, *N*, *K*), and iteration indices (e.g., *i*, *k*, *n*) are strictly presented in italics.

All these formatting corrections and standardizations have been clearly highlighted in red in the revised manuscript. We believe that these extensive revisions have significantly enhanced the uniformity, readability, and overall professional presentation of our mathematical notations. We hope these changes fully meet your expectations.

[4] **In the manuscript, the CEEMD method adopts an ensemble number of (I = 100). However, the authors do not provide any justification for this parameter choice or discuss its influence on the MT data processing results. Since both the decomposition performance and computational cost of CEEMD are affected by the ensemble number, the authors are encouraged to conduct a parameter sensitivity analysis. Specifically, it would be beneficial to evaluate the performance of the proposed method under different noise levels and ensemble numbers, thereby demonstrating the rationality of the selected parameter setting and assessing the robustness of the method with respect to parameter variations**

**Response:** Thank you very much for this valuable and constructive comment. We agree that the original manuscript did not provide sufficient justification for selecting the CEEMD ensemble number (*I* = 100), nor did it adequately discuss the influence of this parameter on denoising performance and computational cost.

In response, we conducted an additional parameter sensitivity analysis using the same synthetic mixed-noise signal and signal-reconstruction criterion as those employed in the simulation experiment. In accordance with the definition used in the manuscript, *I* denotes the number of Gaussian white-noise realizations used in the CEEMD ensemble. Four representative ensemble numbers (*I* = 20, 100, 200, and 500) were evaluated under two input noise levels corresponding to SNRs of 0 dB and −3 dB. During the analysis, the standard deviation of the Gaussian white noise added by CEEMD was fixed at 0.2 times that of the noisy input signal, while all other parameters were held constant.

For each combination of input SNR and ensemble number, the CEEMD decomposition was independently repeated three times using independently generated Gaussian white-noise ensembles while retaining the same input noisy signal. Output SNR, mean squared error (MSE), normalized cross-correlation (NCC), and computational runtime were evaluated. The reported markers represent the mean values of the three runs, and the error bars indicate ±1 standard deviation.

The results show that increasing *I* from 20 to 100 generally improves the denoising performance under both tested noise levels. When *I* is further increased to 200 or 500, only relatively small variations are observed, and no consistent additional improvement is obtained across all evaluation metrics and noise levels. By contrast, the computational runtime continues to increase substantially with *I*, particularly at *I* = 500. These results indicate that the performance of the proposed method tends to stabilize at approximately *I* = 100 under the tested conditions. Therefore, *I* = 100 was selected as a reasonable compromise between denoising performance and computational efficiency.

The complete parameter sensitivity analysis, including the experimental settings, quantitative results, explanatory discussion, and figure caption, has been added as S1 Appendix. The corresponding description in the main manuscript has also been revised to state that *I* = 100 was selected based on the parameter sensitivity analysis presented in S1 Appendix.

**The revised portions of the manuscript are marked in red.**

**Section 1.2:**

First, a single-channel MT signal to be analyzed is denoted by *x*. A total of *I* Gaussian white-noise realizations are added to the signal, where *I* is set to 100 based on the parameter sensitivity analysis presented in S1 Appendix. The resulting noise-assisted sequences are constructed as follows:

**S1 Appendix. Sensitivity analysis of the CEEMD ensemble number**

To evaluate the sensitivity of the proposed method to the CEEMD ensemble number *I*, the same synthetic mixed-noise signal and signal-reconstruction criterion as those used in the simulation experiment were adopted. Four representative ensemble numbers (*I* = 20, 100, 200, and 500) were evaluated under input SNRs of 0 dB and −3 dB. During the sensitivity analysis, the standard deviation of the noise added by CEEMD was fixed at 0.2 times that of the noisy input signal, and all parameters other than *I* were held constant. Here, *I* denotes the number of Gaussian white-noise realizations used in the CEEMD ensemble. For each parameter combination, the CEEMD decomposition was repeated three times using independent realizations of the added noise while retaining the same input noisy signal. Output SNR, mean squared error (MSE), normalized cross-correlation (NCC), and computational runtime were used as evaluation metrics. The markers in Fig. A1 indicate the mean values of the three runs, and the error bars indicate ±1 standard deviation (SD).

As shown in Fig. A1, increasing *I* from 20 to 100 generally improves the denoising performance under both tested input SNR levels, as indicated by the increase in output SNR, decrease in MSE, and improvement in NCC. When *I* is further increased to 200 or 500, only relatively small variations are observed, and no consistent additional improvement is obtained across all metrics and noise levels. This indicates that the denoising performance tends to stabilize at approximately *I* = 100. In contrast, the computational runtime increases continuously with *I*, with a particularly substantial increase at *I*= 500.

Therefore, considering both denoising performance and computational efficiency, *I* = 100 was considered a reasonable performance–cost compromise among the tested ensemble numbers and was adopted in the proposed method.

Fig. A1. Sensitivity analysis of the CEEMD ensemble number under input SNRs of 0 dB and −3 dB. (a) Output SNR, (b) mean squared error (MSE), (c) normalized cross-correlation (NCC), and (d) computational runtime. Each parameter combination was evaluated using three independent CEEMD runs. Markers indicate the mean values, and error bars indicate ±1 standard deviation (SD).

[5] **In the definitions of key evaluation metrics such as SNR and MSE, the physical meanings of (x(n)) and (y(n)) are not described with sufficient clarity. In particular, for denoising applications, it should be explicitly stated whether these variables represent the noisy signal, the reference clean signal, or the denoised signal. The current presentation may lead to ambiguity and makes it difficult for readers to clearly understand how these evaluation metrics are computed. The authors are therefore encouraged to provide the necessary explanations near the corresponding equations and to ensure consistent definitions of all related symbols throughout the manuscript.**

**Response:** Thank you for this highly constructive and rigorous comment. We completely agree that the previous descriptions of *x(n)* and *y(n)* were not sufficiently explicit and could cause ambiguity in the context of denoising evaluation.

First, we have revised the explanation near Equations (12)-(15) in Section 2 to explicitly clarify that, in the synthetic experiments, *x(n)* denotes the reference clean MT signal before adding artificial noise, whereas *y(n)* denotes the denoised signal output by a given processing method. We also clarified that the artificially noisy signal is used solely as the input to the denoising methods, but not as the reference signal for calculating SNR, NCC, E, and MSE. The discrete sample index $\text{n}$ and the total number of samples *N* have also been explicitly defined.

Furthermore, taking your advice very seriously, we have conducted a comprehensive review and meticulous revision of the entire manuscript. We thoroughly checked every single formula to ensure that the physical meaning of every variable is clearly explained immediately following its corresponding equation. We have standardized all mathematical notations to guarantee absolute consistency and to eliminate any notational conflicts throughout the text.

All these modifications are clearly highlighted in red in the revised manuscript. While numerous minor notational corrections are not listed here to avoid redundancy, two major revisions regarding variable definitions are detailed below:

**Line 182-195:**

Large-scale noise and complex interferences (such as square waves and impulse noise) are added to the low-noise MT signals. The results of different denoising methods are then compared to verify the effectiveness and robustness of the proposed approach. For the synthetic tests, since the clean reference signal is known prior to the addition of artificial noise, the denoising performance can be quantitatively evaluated by comparing the denoised signal against this reference. The signal-to-noise ratio (SNR), normalized cross-correlation (NCC), fitting error E, and mean squared error (MSE) are used to quantitatively analyze the improvement effects of the processing methods. These parameters are defined as follows [41,42]:

$$\begin{aligned} \text{SNR}\text{=20}\lg\frac{\left\| \text{y}\text{(}\text{n}\text{)} \right\|_{\text{2}}}{\left\| \text{y}\text{(}\text{n}\text{)−}\text{x}\text{(}\text{n}\text{)} \right\|_{\text{2}}}\#\text{(12)} \end{aligned}$$

$$\begin{aligned} \text{NCC}\text{=}\frac{\sum_{\text{n}\text{=1}}^{\text{N}} \text{y}\text{(}\text{n}\text{)}\text{⋅}\text{x}\text{(}\text{n}\text{)}}{\sqrt{\left( \sum_{\text{n}\text{=1}}^{\text{N}} \text{y}^{\text{2}}\text{(}\text{n}\text{)} \right)}\text{⋅}\sqrt{\left( \sum_{\text{n}\text{=1}}^{\text{N}} \text{x}^{\text{2}}\text{(}\text{n}\text{)} \right)}}\#\text{(13)} \end{aligned}$$

$$\begin{aligned} \text{E}\text{=}\frac{\left\| \text{y}\text{(}\text{n}\text{)−}\text{x}\text{(}\text{n}\text{)} \right\|_{\text{2}}}{\left\| \text{y}\text{(}\text{n}\text{)} \right\|_{\text{2}}}\#\text{(14)} \end{aligned}$$

$$\begin{aligned} \text{MSE}\text{=}\frac{{\sum_{\text{n}\text{=1}}^{\text{N}} \left( \text{y}\text{(}\text{n}\text{)−}\text{x}\text{(}\text{n}\text{)} \right)}^{\text{2}}}{\text{N}}\#\text{(15)} \end{aligned}$$

where *x(n)* represents the reference clean MT signal (i.e., the baseline low-noise signal prior to adding synthetic noise), *y(n)* denotes the denoised signal output by the processing method, and $\text{n}$ is the discrete sample index, *N* is the total number of samples, and $\left\| \cdot\right\|_{\text{2}}$ denotes the L2 norm of the vector. The artificial noisy signal is used solely as the input for the denoising procedures and is not used as a reference for metric calculation. Therefore, a better denoising effect is characterized by higher SNR and NCC values, corresponding to lower E and MSE values.

**Line 123-136:**

The time series vectors $\text{x}_{\text{i}}$ are used to construct a real matrix $\text{X}\text{=[}\text{x}_{\text{1}}^{\text{T}}\text{x}_{\text{2}}^{\text{T}}\text{…}\text{x}_{\text{n}}^{\text{T}}\text{]}$，where ${\text{X}\text{∈}\text{ℝ}}^{\text{m}\text{×}\text{n}}$，SVD is then applied to the matrix *X* [37,38]*:*

$$\begin{aligned} \text{X}\text{=}\text{USV}^{\text{T}}\#\text{(1)} \end{aligned}$$

where $\text{X}$ represents the multidimensional input matrix constructed from the original noisy MT signal, and the main diagonal elements of ${\text{S}\text{∈}\text{ℝ}}^{\text{m}\text{×}\text{n}}$ are the singular values of the matrix $\text{X}$. The superscript T denotes the transpose operation,$\text{p}\text{=}\text{min(}\text{m}\text{,}\text{n}\text{)}$, and $\text{σ}_{\text{1}}\text{≥}\text{σ}_{\text{2}}\text{≥}\text{⋯}\text{≥}\text{σ}_{\text{p}}\text{≥}\text{0}\text{ }\text{for}\text{ }\text{σ}_{\text{i}}\text{(}\text{i}\text{=1,2,}\text{⋯}\text{,}\text{ }\text{p}\text{)}$. *U* is an $\text{m}\text{×}\text{m}\text{ }$orthogonal matrix of left singular vectors, and *V* is an $\text{n}\text{×}\text{n}$ orthogonal matrix of right singular vectors.

In SVD, the input data must be a multidimensional matrix. Therefore, it is necessary to transform the one-dimensional MT time series into a multidimensional matrix using Hankel matrices. Additionally, the components obtained from SVD can be divided into effective signals and noise:

$$\begin{aligned} \text{X}\text{=}\text{X}_{\text{s}}\text{+}\text{N}\text{=[}\text{U}_{\text{s}}\cdots\text{U}_{\text{n}}\text{]}\left[ \begin{matrix} \text{S}_{\text{s}} & \cdots& 0 \\ \vdots& \ddots& \vdots\\ 0 & \cdots& \text{S}_{\text{n}} \end{matrix} \right]\left[ \begin{matrix} \text{V}_{\text{s}}^{\text{T}} \\ \vdots\\ \text{V}_{\text{n}}^{\text{T}} \end{matrix} \right]\#\text{(2)} \end{aligned}$$

where${\text{ }\text{X}}_{\text{s}}$ represents the effective signal matrix, and $\text{N}$ represents the noise matrix. The subscript $\text{s}$ denotes the effective signal components, and the subscript n denotes the noise components in the decomposed singular vectors and values.

[6] **Figures 12 and 13 present the time-domain results of Sites 48 and 49 processed using conventional SVD, CEEMD, and the proposed method. However, the original signal is only shown in Fig. 11. As a result, readers need to switch between multiple figures to perform comparative analysis, which negatively affects the readability of the manuscript. In addition, the current figures do not display the original signal together with the results of different methods, making it difficult to visually assess the level of noise reduction and signal fidelity. Moreover, the presented time windows are relatively short, the number of sampling points is limited, and only a single channel is provided. Considering that MT data typically include multiple channels such as EX, EY, HX, and HY, and that effective electromagnetic responses exhibit strong inter-channel coherence while random noise generally does not, presenting only single-channel waveforms is insufficient to fully demonstrate the effectiveness of the proposed method in signal recovery. The authors are encouraged to include comparative results for multiple channels (EX, EY, HX, and HY) and to further compute quantitative metrics such as coherence coefficients between channels before and after denoising, in order to evaluate the ability of the proposed method to suppress noise while preserving inter-channel signal consistency. Furthermore, it is recommended to present the original signal, conventional methods, and the proposed method within the same figure for direct comparison, and to include zoomed-in views, error analyses, or frequency-domain comparisons where appropriate, so as to more intuitively and comprehensively demonstrate the advantages of the proposed method in terms of noise suppression and signal fidelity.**

**Response:** Thank you very much for this valuable and constructive comment. We fully agree that presenting the raw signal together with the results of different denoising methods can facilitate direct comparison, and that multichannel analysis is important for evaluating the applicability of an MT denoising method. Following the your suggestions, we have carefully revised the relevant figures, descriptions, and discussion in the manuscript.

First, Figures 12 and 13 have been revised to display the raw signal together with the corresponding results obtained using conventional SVD, CEEMD, and the proposed method. In each pair of panels, the raw signal and its spectrum are shown in red, while the corresponding denoised results are shown in black. Both time-domain waveforms and frequency-domain spectra are presented in the same figures. The figure captions and the related discussion in Section 3 have also been revised accordingly. These modifications allow readers to directly evaluate the differences in noise suppression and signal preservation among the three methods without switching between Figures 11-13.

Second, to extend the field-data evaluation beyond a single channel, we have added the processing results of the Ex, Ey, Hx, and Hy components at Site 48 obtained using the proposed method. These multichannel results, together with the corresponding explanatory paragraph and figure caption, are now presented as Figure 14 in the revised manuscript. The added results demonstrate the applicability of the proposed method to both electric and magnetic field components and extend the field-data evaluation from a single component to the four routinely recorded MT channels.

We also appreciate the your suggestions concerning the displayed time-window length and the quantitative evaluation of inter-channel coherence. The present figures retain the representative data interval used in the original comparison because this interval contains the characteristic noise waveforms and allows the local differences among the denoising methods to remain clearly visible. We acknowledge, however, that this relatively short interval does not constitute a long-duration multichannel validation.

Furthermore, the newly added four-channel results provide a qualitative demonstration of channel-wise applicability, but they do not constitute a full multichannel comparison among conventional SVD, CEEMD, and the proposed method, nor do they quantitatively establish the preservation of inter-channel coherence. Rather than reporting coherence coefficients that may be sensitive to the selected data interval and spectral parameters, we have identified this issue as an important direction for further investigation.

Accordingly, the Conclusion has been revised to state that future research will analyze longer continuous and synchronized multichannel MT records to evaluate the preservation of electric-magnetic relationships after denoising. Frequency-dependent inter-channel coherence has also been incorporated into the proposed broader evaluation framework.

We sincerely appreciate your comment, which has helped us improve the direct comparability of the figures, broaden the field-data presentation to multiple MT components, and clarify the limitations and future validation requirements of the present study.

**All corresponding revisions in the manuscript are marked in red:**

Figure 12. Direct comparison of the raw and denoised signals at Site 49 in the time and frequency domains. The raw signal and its spectrum are shown in red, whereas the denoised results are shown in black. Panels (a) and (b) present the results obtained using conventional SVD; panels (c) and (d) present the results obtained using CEEMD; and panels (e) and (f) present the results obtained using the proposed method. Panels (a), (c), and (e) show the time-domain signals, while panels (b), (d), and (f) show the corresponding amplitude spectra.

Figure 13. Direct comparison of the raw and denoised signals at Site 48 in the time and frequency domains. The raw signal and its spectrum are shown in red, whereas the denoised results are shown in black. Panels (a) and (b) present the results obtained using conventional SVD; panels (c) and (d) present the results obtained using CEEMD; and panels (e) and (f) present the results obtained using the proposed method. Panels (a), (c), and (e) show the time-domain signals, while panels (b), (d), and (f) show the corresponding amplitude spectra.

To further examine the applicability of the proposed method to different MT components, the Ex, Ey, Hx, and Hy channels at Site 48 were processed using the proposed method, as shown in Figure 14. The results demonstrate that the proposed processing workflow is applicable to both electric and magnetic field components, thereby extending the field-data evaluation from a single component to the four routinely recorded MT channels.

Figure 14. Multichannel MT time series after denoising using the proposed method at Site 48. (a) Ex, (b) Ey, (c) Hx, and (d) Hy. The four components are displayed using channel-specific vertical scales because of their different physical units and amplitude ranges.

**Conclusion:**

Furthermore, a broader evaluation framework would help to better contextualize the performance and applicability of the proposed method. The present study focused on comparisons with traditional SVD, standalone CEEMD, and the Robust method, which are directly related to the motivation and workflow of the proposed approach. Future research will extend the benchmark evaluation to include other representative denoising methods, such as wavelet transform, Hilbert–Huang transform, and variational mode decomposition, under different noise types and field conditions. **Longer continuous and synchronized multichannel MT records will also be analyzed to evaluate the preservation of electric–magnetic relationships after denoising.** Accordingly, evaluation criteria such as SNR improvement, MSE, runtime, parameter sensitivity, practical applicability, and **frequency-dependent inter-channel coherence** will be considered where appropriate to provide a more systematic assessment of MT denoising strategies.

[7] **The image resolution of Figures 14 and 15 is relatively low, and some details are difficult to discern, which affects the analysis of the results. The authors are advised to improve the resolution of the figures and optimize the font size, axis labels, and legend presentation to ensure that all key information is clearly and effectively displayed.**

**Response**: Thank you for this constructive comment. We agree that the resolution and visual clarity of Figures 14 and 15 in the previous version were insufficient, which may have affected the readability of the apparent resistivity and phase curves and the comparison among different denoising methods.

In the revised manuscript, we have replaced Figures 14 and 15 with higher-resolution versions, and the corresponding figure captions have been retained and checked for consistency.

The revised parts in the manuscript have been marked in red.

Figure 14. Comparison of apparent resistivity and phase curves for Site 49 before and after denoising using different methods.

(a) The apparent resistivity and phase curves of the raw data; (b) results processed by the traditional SVD method; (c) results processed by the CEEMD method; (d) results processed by the Robust method; and (e) results processed by the proposed method.

Figure 15. Comparison of apparent resistivity and phase curves for Site 48 before and after denoising using different methods.

(a) The apparent resistivity and phase curves of the raw data; (b) results processed by the traditional SVD method; (c) results processed by the CEEMD method; (d) results processed by the Robust method; and (e) results processed by the proposed method.
[8] **In the introduction, the proposed SVD-CEEMD method is explicitly positioned as a data-driven and unsupervised signal processing framework, with the claim that it can avoid the reliance on large-scale prior data and the high computational cost associated with dictionary learning and deep learning methods, which is presented as one of its key advantages. However, in the conclusion section, the authors further state that neural networks may be incorporated in future work to address over-processing issues and improve signal separation performance. This reveals a certain inconsistency in terms of research positioning and methodological direction. If the authors consider that traditional signal processing methods are already sufficient to effectively overcome the limitations of deep learning approaches in MT signal denoising, further clarification is needed regarding the necessity and specific role of introducing neural networks in future work. Conversely, if neural networks still play an indispensable role in improving denoising performance or addressing key challenges, the relationship between the proposed method and deep learning approaches should be described more objectively, rather than emphasizing the "avoidance of deep learning limitations "as a primary advantage in the introduction. The authors are encouraged to carefully reconsider and clarify the positioning of the proposed method, explicitly defining whether its relationship with deep learning methods is one of replacement, complementarity, or staged improvement, so as to enhance the logical consistency and academic rigor of the manuscript.**

**Response:** We sincerely thank the reviewer for this insightful and constructive comment. We completely agree that our previous wording in the Introduction and Conclusion created a logical inconsistency regarding the research positioning of the proposed method.

Following your valuable suggestion, we have carefully reconsidered and explicitly defined the relationship between our SVD-CEEMD method and deep learning approaches as **complementary**.

Specifically, the proposed SVD-CEEMD method serves as a lightweight, unsupervised, and physically interpretable signal-processing framework that is highly effective under limited prior-data conditions. We do not consider heavy neural networks to be indispensable components of the core signal separation task. Instead, we clarify that future lightweight statistical or feature-based classification approaches could serve a complementary role by providing auxiliary decision information (e.g., noise-type identification) for adaptive workflow control, preventing over-processing without sacrificing computational efficiency.

To reflect this clarified positioning and enhance the academic rigor of the manuscript, we have made the following revisions:

**1. Revisions to the Introduction**

We revised the text to avoid overemphasizing the “avoidance of deep learning limitations” as an absolute advantage. The revised text now reads:

“The proposed method does not require a pre-trained dictionary or large-scale labeled training datasets, and therefore provides a lightweight and unsupervised alternative for MT denoising under limited prior-data conditions.”

**2. Revisions to the Conclusion**

We revised the conclusion to remove the broad mention of “neural networks” and to clarify the specific, complementary role of future auxiliary methods. The revised text now reads:

“Regarding the limitations and future development of the proposed method, similar to other current denoising approaches, slight attenuation of the underlying signals may still occur after processing. In addition, large-scale noise and abrupt noise do not always co-occur in real MT data; therefore, applying the complete two-stage processing procedure to all data segments may occasionally lead to over-processing. Future work should focus on adaptive workflow control, including noise-type identification, parameter selection, and the choice between single-stage and two-stage processing. Lightweight statistical or feature-based classification approaches will be explored to provide auxiliary decision information prior to signal reconstruction. This strategy may reduce unnecessary computational overhead while maintaining the interpretability and low-prior-data requirements of the proposed SVD-CEEMD framework.”

We hope that these revisions meet your expectations by explicitly defining the relationship between the proposed method and learning-based approaches, thereby resolving the logical inconsistency in the manuscript.

[9] **The manuscript repeatedly emphasizes that the proposed method has advantages such as low computational cost, no requirement for prior training data, and a simple processing workflow. However, a systematic comparison with representative traditional MT signal denoising methods is currently missing. In particular, widely used approaches in electromagnetic signal denoising, such as wavelet transform and the Hilbert–Huang transform, have not been included for comparison. As a result, no quantitative evidence is provided to demonstrate how the proposed method performs relative to these classical techniques in terms of denoising effectiveness, computational efficiency, parameter dependency, and applicability. Therefore, the claims regarding the advantages of the proposed method are not yet sufficiently supported by experimental evidence. The authors are encouraged to include comparative experiments with typical traditional denoising methods and to conduct a comprehensive evaluation from multiple perspectives, including SNR improvement, MSE, runtime, algorithmic complexity, and parameter sensitivity. Such an expanded analysis would provide a more objective and comprehensive validation of the proposed method's performance advantages and strengthen the credibility and persuasiveness of the conclusions.**

**Response:** Thank you very much for this valuable and insightful comment. We fully agree that a broader and more systematic comparison with representative traditional MT denoising methods, such as wavelet transform and Hilbert-Huang transform, would further strengthen the quantitative validation and contextualization of the proposed method.

In response to this comment, we first carefully reviewed the manuscript and revised several expressions that may have sounded overly general or insufficiently constrained. In particular, we made the claims regarding computational efficiency, workflow simplicity, and performance advantages more cautious and more clearly limited to the methods compared in this study. For example, in the Introduction, the original sentence:

“This joint strategy resolves both the boundary artifacts of traditional SVD and the edge suppression issues of standalone CEEMD, yielding superior signal-to-noise separation and computational efficiency.”

has been revised as:

“This joint strategy improves both the boundary artifacts of traditional SVD and the edge suppression issues of standalone CEEMD, yielding better signal-to-noise separation in the tested cases.”

This revision makes the scope of the statement more precise and avoids implying that the proposed method has been proven superior to all existing denoising methods. It also removes the overly broad claim regarding computational efficiency because a full runtime comparison with a broader set of representative traditional methods was not conducted in the present study. Similar wording adjustments have been made elsewhere in the manuscript, and the revised parts have been marked in red.

Regarding the comparative experiments, the baseline methods in this study were selected according to the methodological motivation and application context of the proposed approach. The proposed method is developed by improving traditional SVD with CEEMD; therefore, comparisons with traditional SVD and standalone CEEMD are essential for directly demonstrating the contribution of each component in the proposed workflow. In addition, the Robust method was included in the field-data comparison because it is a commonly used MT processing strategy.

We fully agree that wavelet transform, HHT, and other representative traditional denoising methods are also important references for a broader evaluation of MT denoising performance. In particular, HHT is closely related to EMD-based methods, while CEEMD can be regarded as an improved noise-assisted extension of EMD. Thus, including HHT and wavelet-based methods in a future benchmark comparison would provide a more comprehensive understanding of the advantages and applicability of the proposed method. In the revised manuscript, we have added this issue as an important future research direction in the Conclusion. Future work will extend the comparative framework to include wavelet transform, HHT, VMD, and other representative methods, and will evaluate them under different noise types and field conditions using comprehensive indicators such as SNR improvement, MSE, runtime, parameter sensitivity, and applicability.

The revised parts in the manuscript have been marked in red.

The following statement has been added to the Conclusion:

**Furthermore, a broader evaluation framework would help to better contextualize the performance and applicability of the proposed method. The present study focused on comparisons with traditional SVD, standalone CEEMD, and the Robust method, which are directly related to the motivation and workflow of the proposed approach. Future research will extend the benchmark evaluation to include other representative denoising methods, such as wavelet transform, Hilbert–Huang transform, and variational mode decomposition, under different noise types and field conditions.** Longer continuous and synchronized multichannel MT records will also be analyzed to evaluate the preservation of electric–magnetic relationships after denoising. **Accordingly, evaluation criteria such as SNR improvement, MSE, runtime, parameter sensitivity, practical applicability, and** frequency-dependent inter-channel coherence **will be considered where appropriate to provide a more systematic assessment of MT denoising strategies.**

**Thank you again for your time and efforts to review our paper.**
