# Supplementary material for: Research on magnetotelluric signal noise separation based on improved SVD
Source: PLoS One. 2026 Nov 5;21(11):e0334468. doi: 10.1371/journal.pone.0334468 (PMC13739181; doi:10.1371/journal.pone.0334468)
Supplement: Supplementary file 5 [file pone.0334468.s006.docx]

**Manuscript ID: PONE-D-25-11846R4**

**Manuscript Title: Research on magnetotelluric signal noise separation based on improved SVD**

**Dear Dr Feier Chen:**

We greatly appreciate your letter regarding our manuscript (**ID: PONE-D-25-11846R3**) entitled “Research on magnetotelluric signal noise separation based on improved SVD”. We are also extremely grateful for the constructive comments provided by you and the reviewers. We have carefully considered every comment and made thorough revisions accordingly.

To facilitate the evaluation process for you and the reviewers, all modifications have been highlighted in red in the revised manuscript. Furthermore, based on their valuable suggestions, we have provided detailed, point-by-point responses to each question in the accompanying document.

We sincerely hope that our revisions meet your expectations and align with the rigorous academic standards of PLOS ONE. Should you have any further questions regarding this manuscript, please do not hesitate to contact us at your earliest convenience.

Most sincerely,

Fuming Qu

Sept 3, 2026

**Dear Reviewer #4:**

We sincerely thank you for your careful evaluation of our revised manuscript and for your positive assessment of the revisions made in response to the previous round of review. We greatly appreciate your recognition of the improvements in the overall quality of the manuscript.

We have carefully considered your further comments and suggestions and have revised the manuscript accordingly. Your thoughtful feedback has helped us further improve the accuracy, clarity, and completeness of the manuscript. To facilitate your review, all modifications have been clearly highlighted in red in the revised manuscript.

We sincerely hope that these revisions have adequately addressed your remaining concerns and that the revised manuscript now meets the publication standards of PLOS ONE.

Most sincerely,

Fuming Qu

The following are our detailed, point-by-point responses to your comments:

**Comments:**

[1] **Thank you to the authors for their careful revisions in response to the previous round of review. After carefully examining the revised manuscript and the point-by-point response letter, I can see that the authors have adequately addressed and revised most of the comments I raised previously. The overall quality of the manuscript has improved significantly compared with the R2 version. However, upon a quick check, I still noticed a minor spelling error. For example, on page 7, line 186, “signa” should be corrected to “signal.” I recommend that the authors carefully proofread the entire manuscript before final submission to avoid similar spelling errors.**

**Response**: We sincerely thank you for bringing this spelling error to our attention, and we apologize for the oversight. We have corrected “signa” to “signal” on page 7, line 186. Following your recommendation, we also carefully proofread the entire manuscript and corrected other spelling and typographical errors identified during this process. All corresponding changes have been highlighted in red in the revised manuscript.

[2] **In addition, the authors indicate that future work will focus on adaptive workflow control, including noise-type identification, parameter selection, and the selection between single-stage and two-stage processing strategies. They also plan to explore lightweight statistical or feature-based classification methods to provide auxiliary decision-making information prior to signal reconstruction. In this regard, I suggest that the authors further consider existing studies using deep learning models, such as CNN and CNN-LSTM, for noise-type identification, signal-quality assessment, and adaptive processing strategies. For example, the relevant studies by Wang et al. (2024, JGR: Solid Earth), Li et al. (2023, Geophysics), and Zhang et al. (2024, IEEE TGRS) may provide useful references. These studies could provide valuable insights for the future development of the proposed framework, particularly in terms of noise identification, processing-strategy selection, and adaptive workflow control.**

**Response:** We sincerely thank you for this valuable and insightful suggestion. We agree that deep learning models, such as CNN and CNN-LSTM, offer promising possibilities for noise-type identification, signal-quality assessment, and adaptive processing-strategy selection in MT data processing.

Following your suggestion, we carefully studied and cited the three recommended articles by Wang et al. (2024), Li et al. (2023), and Zhang et al. (2024) [43-45]. We have also revised the discussion of future work in the Conclusion section. In addition to the originally proposed lightweight statistical or feature-based classification methods, the revised manuscript now identifies deep learning models as a complementary approach for the future development of adaptive workflow control. We further clarify that these approaches will be evaluated in terms of identification accuracy, signal-quality assessment, computational cost, interpretability, and training-data requirements to determine their suitability for noise identification and adaptive processing-strategy selection within the proposed SVD-CEEMD framework.

This suggestion has enabled us to broaden the scope of our future research and present a more comprehensive discussion of the potential development of the proposed framework.

The revised portions of the manuscript are marked in red:

Regarding the limitations and future development of the proposed method, similar to other current denoising approaches, slight attenuation of the underlying signals may still occur after processing. In addition, large-scale noise and impulsive noise do not always co-occur in real MT data; therefore, applying the complete two-stage processing procedure to all data segments may occasionally lead to over-processing. Future work will focus on adaptive workflow control, including noise-type identification, parameter selection, and the choice between single-stage and two-stage processing. To provide auxiliary decision information prior to signal reconstruction, two complementary approaches will be investigated. Lightweight statistical or feature-based classification methods will be considered because of their computational efficiency, interpretability, and limited reliance on prior training data. Meanwhile, recent studies have demonstrated the potential of deep learning models, including CNN and CNN-LSTM architectures, for MT noise identification and suppression **[43-45]**. These approaches will be evaluated in terms of identification accuracy, signal-quality assessment, computational cost, interpretability, and training-data requirements to determine their suitability for adaptive processing-strategy selection within the proposed SVD-CEEMD framework.

**Thank you again for your time and efforts to review our paper.**
