# Supplementary material for: Research on magnetotelluric signal noise separation based on improved SVD
Source: PLoS One. 2026 Nov 5;21(11):e0334468. doi: 10.1371/journal.pone.0334468 (PMC13739181; doi:10.1371/journal.pone.0334468)
Supplement: Peer review history

# Peer review: Decision Letter 0

**DOI:** 10.1371/journal.pone.0334468.r001

**Authors:** Jason Morgan

2 Jul 2025

Dear Dr. YUE,

Thank you for submitting your manuscript to PLOS ONE. After careful consideration, we feel that it has merit but does not fully meet PLOS ONE’s publication criteria as it currently stands. Therefore, we invite you to submit a revised version of the manuscript that addresses the points raised during the review process.

Please submit your revised manuscript by Aug 15 2025 11:59PM. If you will need more time than this to complete your revisions, please reply to this message or contact the journal office at plosone@plos.org. When you're ready to submit your revision, log on to https://www.editorialmanager.com/pone/ and select the 'Submissions Needing Revision' folder to locate your manuscript file.

- A rebuttal letter that responds to each point raised by the academic editor and reviewer(s). You should upload this letter as a separate file labeled 'Response to Reviewers'.
- A marked-up copy of your manuscript that highlights changes made to the original version. You should upload this as a separate file labeled 'Revised Manuscript with Track Changes'.
- An unmarked version of your revised paper without tracked changes. You should upload this as a separate file labeled 'Manuscript'.

If you would like to make changes to your financial disclosure, please include your updated statement in your cover letter. Guidelines for resubmitting your figure files are available below the reviewer comments at the end of this letter.

If applicable, we recommend that you deposit your laboratory protocols in protocols.io to enhance the reproducibility of your results. Protocols.io assigns your protocol its own identifier (DOI) so that it can be cited independently in the future. For instructions see: https://journals.plos.org/plosone/s/submission-guidelines#loc-laboratory-protocols. Additionally, PLOS ONE offers an option for publishing peer-reviewed Lab Protocol articles, which describe protocols hosted on protocols.io. Read more information on sharing protocols at https://plos.org/protocols?utm_medium=editorial-email&utm_source=authorletters&utm_campaign=protocols.

We look forward to receiving your revised manuscript.

Kind regards,

Dr Jason Morgan

Staff Editor

PLOS ONE

Journal requirements:

When submitting your revision, we need you to address these additional requirements.

1. Please ensure that your manuscript meets PLOS ONE's style requirements, including those for file naming. The PLOS ONE style templates can be found at

https://journals.plos.org/plosone/s/file?id=wjVg/PLOSOne_formatting_sample_main_body.pdf and

https://journals.plos.org/plosone/s/file?id=ba62/PLOSOne_formatting_sample_title_authors_affiliations.pdf

2. We note that your Data Availability Statement is currently as follows: [All relevant data are within the manuscript and its Supporting Information files.]

Please confirm at this time whether or not your submission contains all raw data required to replicate the results of your study. Authors must share the “minimal data set” for their submission. PLOS defines the minimal data set to consist of the data required to replicate all study findings reported in the article, as well as related metadata and methods (https://journals.plos.org/plosone/s/data-availability#loc-minimal-data-set-definition).

For example, authors should submit the following data:

- The values behind the means, standard deviations and other measures reported;

- The values used to build graphs;

- The points extracted from images for analysis.

Authors do not need to submit their entire data set if only a portion of the data was used in the reported study.

If your submission does not contain these data, please either upload them as Supporting Information files or deposit them to a stable, public repository and provide us with the relevant URLs, DOIs, or accession numbers. For a list of recommended repositories, please see https://journals.plos.org/plosone/s/recommended-repositories.

If there are ethical or legal restrictions on sharing a de-identified data set, please explain them in detail (e.g., data contain potentially sensitive information, data are owned by a third-party organization, etc.) and who has imposed them (e.g., an ethics committee). Please also provide contact information for a data access committee, ethics committee, or other institutional body to which data requests may be sent. If data are owned by a third party, please indicate how others may request data access.

[Note: HTML markup is below. Please do not edit.]

Reviewers' comments:

Reviewer's Responses to Questions

**Comments to the Author**

1. Is the manuscript technically sound, and do the data support the conclusions?

Reviewer #1: Yes

Reviewer #2: Yes

**********

2. Has the statistical analysis been performed appropriately and rigorously?

Reviewer #1: Yes

Reviewer #2: Yes

**********

3. Have the authors made all data underlying the findings in their manuscript fully available?

The PLOS Data policy

Reviewer #1: No

Reviewer #2: No

**********

4. Is the manuscript presented in an intelligible fashion and written in standard English?

Reviewer #1: Yes

Reviewer #2: Yes

**********

Reviewer #1: The paper by Yue et al. addresses a critical challenge in magnetotelluric (MT) data processing effective suppression of human-induced electromagnetic noise, which increasingly hampers accurate impedance estimation. The authors propose an enhanced signal-to-noise separation method by integrating Singular Value Decomposition (SVD) with Complementary Ensemble Empirical Mode Decomposition (CEEMD). This hybrid approach leverages the amplitude contrast between weak natural MT signals and dominant anthropogenic noise, aiming to overcome the limitations of traditional SVD methods that often leave residual noise artifacts. By demonstrating improved performance on both synthetic and real datasets, the study contributes to a potentially valuable tool for enhancing the reliability of MT interpretations in noisy environments. I appreciate the great piece of work by the authors in this study. I am happy to accept this manuscript for publication after addressing some of my concern on this article.

The following are my comments:

1. Line 11, SVD is first time appeared here, it is recommended to write full form of it and put short form in brackets.

2. Line 16, same as previous comment for CEEMD.

3. This study is focused in Gansu region, Qilian region, but I did not see anything about this region in the introduction part. It is recommended to put at least a paragraph about the study region and show the study area in map view.

4. Line 183-185, you mentioned the signal shown in Figure 2 is synthetic noisy signal and later in the sentence, you mentioned the signal is obtained from Gansu province. Please make it consistent.

5. Line 186-195, Most of the contents in this section is the same as caption provided in Figure. It is better to explain results rather than just repeating captions. Please follow this recommendation throughout the writeup.

6. Line 206, 15Hz correct this as 15 Hz

7. Figures 8b and 8c, why are some points out of the plot?

8. Line 303, it is highly recommended to put station map and show the details around the stations in map. This helps the reader to follow what you want to say. In addition to the station location, if you can also plot the location of industry and population density in map, this will be helpful to readers to follow your article.

Reviewer #2: I have found the work well written and clear.

I couldn't find a link to the data set as declared in the submitting form

In my opinion the parts that can be improved are:

1) References to other impedance estimation methods

It seems to me that some relevant works on this subject have not been cited/briefly discussed:

Eisel M, Egbert GD (2001) On the stability of magnetotelluric transfer function estimates and the reliability of their variances. Geophysical Journal International, Volume 144, Issue 1, January 2001, Pages 65–82, https://doi.org/10.1046/j.1365-246x.2001.00292.x

Rizzello, D., Armadillo, E., Smooth magnetotelluric impedance estimation by optimization, Journal of Applied Geophysics, Volume 210, 2023, 104952, ISSN 0926-9851, https://doi.org/10.1016/j.jappgeo.2023.104952.

Jin Li, Shanshan Liu, Yiqun Peng, Jingtian Tang, Xian Zhang , Yong Li. A method for magnetotelluric data processing based on sparsity adaptive stage-wise orthogonal matching pursuit. Journal of Applied Geophysics, Volume 198, March 2022, 104577. https://doi.org/10.1016/j.jappgeo.2022.104577

Qining ZhanQining ZhanCai LiuCai LiuYang LiuYang LiuPengfei Zhao Pengfei Zhao. Data processing method for magnetotelluric sounding based on cepstral analysis. Front. Earth Sci., 18 October 2023, Sec. Geomagnetism and Paleomagnetism, Volume 11 - 2023 | https://doi.org/10.3389/feart.2023.1183188

Gary D. Egbert. Robust multiple-station magnetotelluric data processing. Geophysical Journal International, Volume 130, Issue 2, August 1997, Pages 475–496, https://doi.org/10.1111/j.1365-246X.1997.tb05663.x

As general reference, you should also consider:

Egbert, G. (2021). Magnetotelluric Data Processing. In: Gupta, H.K. (eds) Encyclopedia of Solid Earth Geophysics. Encyclopedia of Earth Sciences Series. Springer, Cham. https://doi.org/10.1007/978-3-030-58631-7_128

2) Impedance estimation by SVD has been discussed extensively by Egbert and collaborators in the following works, which have not been discussed. A reference to these works and a brief description of their approach should be added, also specifying what has been improved

Egbert, G.D., Booker, J.R. Multivariate analysis of geomagnetic array data: 1. The response space. https://agupubs.onlinelibrary.wiley.com/doi/10.1029/JB094iB10p14227

Egbert, G.D. Multivariate analysis of geomagnetic array data: 2. Random source models. https://agupubs.onlinelibrary.wiley.com/doi/abs/10.1029/JB094iB10p14249

Egbert, G.D.. Robust multiple-station magnetotelluric data processing. Geophysical Journal International, Volume 130, Issue 2, August 1997, Pages 475–496, https://doi.org/10.1111/j.1365-246X.1997.tb05663.x

Egbert, G.D. Processing And Interpretation Of Electromagnetic Induction Array Data. Surveys in Geophysics 23, 207–249 (2002). https://doi.org/10.1023/A:1015012821040

**********

what does this mean?). If published, this will include your full peer review and any attached files.

If you choose “no”, your identity will remain anonymous but your review may still be made public.

**Do you want your identity to be public for this peer review?**  For information about this choice, including consent withdrawal, please see our Privacy Policy

Reviewer #1: No

Reviewer #2: No

**********

[NOTE: If reviewer comments were submitted as an attachment file, they will be attached to this email and accessible via the submission site. Please log into your account, locate the manuscript record, and check for the action link "View Attachments". If this link does not appear, there are no attachment files.]

While revising your submission, please upload your figure files to the Preflight Analysis and Conversion Engine (PACE) digital diagnostic tool, https://pacev2.apexcovantage.com/. PACE helps ensure that figures meet PLOS requirements. To use PACE, you must first register as a user. Registration is free. Then, login and navigate to the UPLOAD tab, where you will find detailed instructions on how to use the tool. If you encounter any issues or have any questions when using PACE, please email PLOS at figures@plos.org. Please note that Supporting Information files do not need this step.

# Author comment: Author response to Decision Letter 1

**DOI:** 10.1371/journal.pone.0334468.r002

15 Sep 2025

Manuscript ID: PONE-D-25-11846

Manuscript Title: Research on magnetotelluric signal noise separation based on improved SVD Decomposition

Dear Dr Jason Morgan:

We are so appreciated for your letter on our manuscript (ID: PONE-D-25-11846) entitled “Research on magnetotelluric signal noise separation based on improved SVD Decomposition”. We are also extremely grateful to the reviewer’s comments on our manuscript and carefully considered every comment, and made cautious revision accordingly. For your and the reviewer’s better check of the revised manuscript, you can find every change in red in our revised manuscript. Based on their suggestions, we have answered the questions in detail one by one.

We sincerely hope our revisions rise to your expectation. If you have any other questions about this manuscript, I would quite appreciate it if you could let me know them in the earliest possible time.

Most sincerely,

Xingtong Yue

Reviewer # 1

The paper by Yue et al. addresses a critical challenge in magnetotelluric (MT) data processing effective suppression of human-induced electromagnetic noise, which increasingly hampers accurate impedance estimation. The authors propose an enhanced signal-to-noise separation method by integrating Singular Value Decomposition (SVD) with Complementary Ensemble Empirical Mode Decomposition (CEEMD). This hybrid approach leverages the amplitude contrast between weak natural MT signals and dominant anthropogenic noise, aiming to overcome the limitations of traditional SVD methods that often leave residual noise artifacts. By demonstrating improved performance on both synthetic and real datasets, the study contributes to a potentially valuable tool for enhancing the reliability of MT interpretations in noisy environments. I appreciate the great piece of work by the authors in this study. I am happy to accept this manuscript for publication after addressing some of my concern on this article.

Dear Reviewer #1:

Thank you very much for all of your insightful comments related to the paper. We are grateful to your recognition of our work. We found your feedback very constructive and tried to be responsive to your concerns. Please fine the revised parts through the manuscript in red highlight. We hope to obtain your approval for publication.

Most sincerely,

Xingtong YUE

The follows are my responses to your suggestions and comments.

Comments:

[1] Line 11, SVD is first time appeared here, it is recommended to write full form of it and put short form in brackets.

Response: Your suggestion is very valid. The full form of SVD should indeed be explained when it first appears in the manuscript to make it easier for readers to understand. The revised parts in the manuscript are marked in red, and the specific content is as follows:

“we propose a magnetotelluric signal-to-noise separation method based on improved Singular Value Decomposition (SVD).”

[2]Line 16, same as previous comment for CEEMD.

Response: Thank you for your valuable comments. We have added the full explanations of the method names in the manuscript. The revised parts in the manuscript are marked in red, and the specific content is as follows:

“Therefore, by combining the Complementary Ensemble Empirical Mode Decomposition (CEEMD) to improve SVD decomposition.”

[3]This study is focused in Gansu region, Qilian region, but I did not see anything about this region in the introduction part. It is recommended to put at least a paragraph about the study region and show the study area in map view.

Response: Thank you for your valuable comments. In the previous manuscript, the focus was on proposing a data processing method for magnetotelluric (MT) sounding rather than interpreting the subsurface structure of the study area. As a result, we only briefly mentioned the location of the study area without elaborating on it. In the revised manuscript, we have displayed the MT field measurement data on a map and added more information about the study area to enhance the logic of the manuscript. The revised parts in the revised manuscript are marked in red. The adjustment of figure numbers in the subsequent content due to the addition of this image has been marked in red. The specific content is as follows:

“Validation through synthetic experiments (MT data in Gansu area) and field datasets (measured data in Qilian area) confirms that this approach optimally mitigates noise morphology, producing continuous and smooth Cagniard apparent resistivity-phase curves. Gansu area is vast and sparsely populated, and the quality of MT data is high. Thus, adding random noise to MT data in Gansu area can confirm the reliability of the proposed method. Certain measured stations in the Qilian area have destroyed by serious industrialization, with poor data quality. Thus, the effectiveness of the proposed method has been confirmed through processing the above measured data. The location of survey areas is shown in Figure 1.”

(Since I am unable to upload the revised images in this text box, please refer to the image in the "Response to Reviewers" attachment.)

Fig 1 The location of survey areas

[4] Line 183-185, you mentioned the signal shown in Figure 2 is synthetic noisy signal and later in the sentence, you mentioned the signal is obtained from Gansu province. Please make it consistent.

Response: I apologize for the confusion and misunderstanding caused in the previous manuscript. The data shown in Figure 2 are indeed synthetic data, but the basis for these synthetic data is derived from real magnetotelluric (MT) measurement data obtained in Gansu Province. Given that MT signals are natural and have a broadband characteristic, it is very difficult to simulate them accurately. Therefore, we used the “clean” MT measurement data from Gansu Province as the basis for our synthetic data. We have rephrased and unified these statements in the revised manuscript to avoid any further misunderstandings. The revised parts in the manuscript are marked in red. The specific content is as follows (due to the increase in the number of figures, Figure 2 in the original text has become Figure 3 in the revised manuscript):

“Figure 3 illustrates the process of generating synthetic noisy signals. Clean MT measured data (collected in Gansu Area, with sampling rate at 15 Hz) serves as the basis for the synthesis process, and different noises are added to generate noisy signals. The sparse population in Gansu Area results in minimal anthropogenic noise, ensuring high-quality MT data acquisition. As observed in Figure 3a, the original signal is stable without any noise waveform.”

[5] Line 186-195, Most of the contents in this section is the same as caption provided in Figure. It is better to explain results rather than just repeating captions. Please follow this recommendation throughout the writeup

Response: Thank you for your valuable comments. In the revised manuscript, we have added content that explains the results, instead of merely repeating the figure captions. The revised parts are marked in red, and the corresponding content is as follows:

“Figures 3d to 3f depict the noisy signals with different types of noise added, such as square niose、impluse noise and mixed noise. It can be seen from Figures 3d to 3f that the morphology of the original MT signal is almost completely obscured, making the time series no longer continuous and smooth. Meanwhile, the amplitude of the original signal was changed (the effective signal energy being much lower than the noise energy), making it difficult to obtain accurate impedance results.”

“Figure 4 shows the corresponding spectra of the original signal and noisy signals in Figure 3. By comparing Figure 4a with Figures 4b and 4d, it is evident that the addition of large-scale noise not only increases the amplitude of the main frequency peak of the effective signal but also introduces multiple side peaks and bandwidths around the main peak, with the amplitude of these side peaks decreasing progressively. This leads to a bias in impedance estimation. From Figures 4a and 4c, it can be seen that impulse noise does not cause a sudden increase in the spectral amplitude but continuously interferes with the entire spectrum.”

[6] Line 206, 15Hz correct this as 15 Hz

Response: We apologize for this error. We have checked the entire text for this mistake and made the necessary corrections. The revised parts are marked in red.

[7] Figures 8b and 8c, why are some points out of the plot?

Response: In the previous manuscript, in an effort to make the differences in the processing results of different methods clearer to the readers, we overlooked the fact that some points exceeded the coordinate range. In the revised manuscript, we have modified the relevant figures. The specific content is as follows (due to the increase in the number of figures, Fig. 8 in the original text has become Fig. 9 in the revised manuscript):

(Since I am unable to upload the revised images in this text box, please refer to the image in the "Response to Reviewers" attachment.)

Fig.9 The apparent resistivity phase curves calculated by the original signal and denoised signal

(a) The apparent resistivity phase curves of the original data; (b) The apparent resistivity phase curves of the noisy signal; (c) The apparent resistivity phase curves of the denoised signal after traditional SVD decomposition; (d) The apparent resistivity phase curves of the denoised signal obtained by CEEMD; (e) The apparent resistivity phase curves of the denoised signal processed by the proposed method.

[8] Line 303, it is highly recommended to put station map and show the details around the stations in map. This helps the reader to follow what you want to say. In addition to the station location, if you can also plot the location of industry and population density in map, this will be helpful to readers to follow your article.

Response: Thank you for your valuable comments. In the revised manuscript, we have added a figure showing the locations of the measurement stations, including Site 49, Site 48, and the industrial area. However, we apologize that we were unable to find specific information regarding population density. The revised parts are marked in red. The specific content is as follows:

(Since I am unable to upload the revised images in this text box, please refer to the image in the "Response to Reviewers" attachment.)

Figure 10. The locations of MT measured stations

Thank you again for your time and efforts to review our paper.

Dear Reviewer #2:

Thank you very much for your comments. We have made revisions in response to each of your suggestions. We have added links to the data that can be accessed and included more appropriate references. The revised parts are marked in red in the revised manuscript. Below are the main corrections in the manuscript and our responses to your comments. We hope to obtain your approval for publication.

Most sincerely,

Xingtong YUE

The follows are my responses to your suggestions and comments.

Reviewer # 2

I have found the work well written and clear.

Comments:

[1] I couldn't find a link to the data set as declared in the submitting form

Response: Your suggestion is very valid. We have added the data source information at the end of the manuscript. The revised parts in the revised manuscript are marked in red. The specific content is as follows:

Supporting information

We provide the measured magnetotelluric data of site48 and site49. The data were collected by Phoenix V5-2000 and processed by SSMT2000.

Site48.tbl and Site49.tbl are the calibration files for the two sites.

Site48.ts3, Site48.ts4 and Site48.ts5 are the high-frequency, medium-frequency and low-frequency magnetotelluric data of site48.

Site49.ts3, Site49.ts4 and Site49.ts5 are the high-frequency, medium-frequency and low-frequency magnetotelluric data of site49.

For easier access and visualization, the original data were converted from their proprietary format into Microsoft Excel.

The high-frequency, medium-frequency and low-frequency magnetotelluric time series data of Site 48 (files Site48.TS3, Site48.TS4, and Site48.TS5) are merged and converted into a single Excel file (Site48.xlsx) , containing the time-domain data of the Site 48. Similarly, the time series data for Site 49 were processed and compiled into a single file (Site49.xlsx).

The data can be downloaded from https://doi.org/10.6084/m9.figshare.30031372.v3

[2] References to other impedance estimation methods

It seems to me that some relevant works on this subject have not been cited/briefly discussed:

Eisel M, Egbert GD (2001) On the stability of magnetotelluric transfer function estimates and the reliability of their variances. Geophysical Journal International, Volume 144, Issue 1, January 2001, Pages 65–82, https://doi.org/10.1046/j.1365-246x.2001.00292.x

Rizzello, D., Armadillo, E., Smooth magnetotelluric impedance estimation by optimization, Journal of Applied Geophysics, Volume 210, 2023, 104952, ISSN 0926-9851, https://doi.org/10.1016/j.jappgeo.2023.104952.

Jin Li, Shanshan Liu, Yiqun Peng, Jingtian Tang, Xian Zhang , Yong Li. A method for magnetotelluric data processing based on sparsity adaptive stage-wise orthogonal matching pursuit. Journal of Applied Geophysics, Volume 198, March 2022, 104577. https://doi.org/10.1016/j.jappgeo.2022.104577

Qining ZhanQining ZhanCai LiuCai LiuYang LiuYang LiuPengfei Zhao Pengfei Zhao. Data processing method for magnetotelluric sounding based on cepstral analysis. Front. Earth Sci., 18 October 2023, Sec. Geomagnetism and Paleomagnetism, Volume 11 - 2023 | https://doi.org/10.3389/feart.2023.1183188

Gary D. Egbert. Robust multiple-station magnetotelluric data processing. Geophysical Journal International, Volume 130, Issue 2, August 1997, Pages 475–496, https://doi.org/10.1111/j.1365-246X.1997.tb05663.x

As general reference, you should also consider:

Egbert, G. (2021). Magnetotelluric Data Processing. In: Gupta, H.K. (eds) Encyclopedia of Solid Earth Geophysics. Encyclopedia of Earth Sciences Series. Springer, Cham. https://doi.org/10.1007/978-3-030-58631-7_128

Response: Thank you for your valuable suggestions. We have added some of the references you listed to the manuscript. The revised parts in the revised manuscript are marked in red.

“Eisel M, Egbert GD. On the stability of magnetotelluric transfer function estimates and the reliability of their variances. Geophysical Journal International, 2001, 144(1): 65–82.

Rizzello D, Armadillo E, Smooth magnetotelluric impedance estimation by optimization, Journal of Applied Geophysics, 2023, 210:104952.

Li J, Liu S, Peng Y et al. A method for magnetotelluric data processing based on sparsity adaptive stage-wise orthogonal matching pursuit. Journal of Applied Geophysics, 2022, 198:104577.

Egbert, G. Magnetotelluric Data Processing. In: Gupta, H.K. (eds) Encyclopedia of Solid Earth Geophysics. Encyclopedia of Earth Sciences Series. Springer, Cham, 2021.”

[3] Impedance estimation by SVD has been discussed extensively by Egbert and collaborators in the following works, which have not been discussed. A reference to these works and a brief description of their approach should be added, also specifying what has been improved

Egbert, G.D., Booker, J.R. Multivariate analysis of geomagnetic array data: 1. The response space.

https://agupubs.onlinelibrary.wiley.com/doi/10.1029/JB094iB10p14227

Egbert, G.D. Multivariate analysis of geomagnetic array data: 2. Random source models. https://agupubs.onlinelibrary.wiley.com/doi/abs/10.1029/JB094iB10p14249

Egbert, G.D.. Robust multiple-station magnetotelluric data processing. Geophysical Journal International, Volume 130, Issue 2, August 1997, Pages 475–496, https://doi.org/10.1111/j.1365-246X.1997.tb05663.x

Egbert, G.D. Processing And Interpretation Of Electromagnetic Induction Array Data. Surveys in Geophysics 23, 207–249 (2002). https://doi.org/10.1023/A:1015012821040

Response: Thank you for your valuable comments. We have incorporated additional references and provided brief descriptions in our manuscript. The specific content is as follows:

Egbert (1997) proposed a multivariate RMEV estimator framework that optimizes MT impedance estimation through automated incoherent-noise diagnosis and robust multichannel signal coherence extraction.

Egbert (2002) further exploited the utilization of the array data to reduce bias, improve signal-to-noise ratios, and provide better control over coherent noise in estimates of MT impedances.

Thank you again for your time and efforts to review our paper.

Attachment Submitted filename: Response to Reviewers.docx

# Peer review: Decision Letter 1

**DOI:** 10.1371/journal.pone.0334468.r003

**Authors:** Brian Weaver

30 Apr 2026

Dear Dr. YUE,

The manuscript has been evaluated by four reviewers, and their comments are available below. One of the reviewers (Reviewer #4) has raised a number of concerns with your manuscript that need attention. Could you please revise the manuscript to carefully address the concerns raised?

Please submit your revised manuscript by Jun 13 2026 11:59PM. If you will need more time than this to complete your revisions, please reply to this message or contact the journal office at ?>plosone@plos.org. When you're ready to submit your revision, log on to https://www.editorialmanager.com/pone/ and select the 'Submissions Needing Revision' folder to locate your manuscript file.

- A letter that responds to each point raised by the academic editor and reviewer(s). You should upload this letter as a separate file labeled 'Response to Reviewers'.
- A marked-up copy of your manuscript that highlights changes made to the original version. You should upload this as a separate file labeled 'Revised Manuscript with Track Changes'.
- An unmarked version of your revised paper without tracked changes. You should upload this as a separate file labeled 'Manuscript'.

If you would like to make changes to your financial disclosure, please include your updated statement in your cover letter. Guidelines for resubmitting your figure files are available below the reviewer comments at the end of this letter.

If applicable, we recommend that you deposit your laboratory protocols in protocols.io to enhance the reproducibility of your results. Protocols.io assigns your protocol its own identifier (DOI) so that it can be cited independently in the future. For instructions see: https://journals.plos.org/plosone/s/submission-guidelines#loc-laboratory-protocols. Additionally, PLOS ONE offers an option for publishing peer-reviewed Lab Protocol articles, which describe protocols hosted on protocols.io. Read more information on sharing protocols at https://plos.org/protocols?utm_medium=editorial-email&utm_source=authorletters&utm_campaign=protocols.

As the corresponding author, your ORCID iD is verified in the submission system and will appear in the published article. PLOS supports the use of ORCID, and we encourage all coauthors to register for an ORCID iD and use it as well. Please encourage your coauthors to verify their ORCID iD within the submission system before final acceptance, as unverified ORCID iDs will not appear in the published article. *Only*  the individual author can complete the verification step; PLOS staff *cannot*  verify ORCID iDs on behalf of authors.

We look forward to receiving your revised manuscript.

Kind regards,

Brian Patrick Weaver, Ph.D.

Staff Editor

PLOS One

Journal Requirements:

If the reviewer comments include a recommendation to cite specific previously published works, please review and evaluate these publications to determine whether they are relevant and should be cited. There is no requirement to cite these works unless the editor has indicated otherwise.

[Note: HTML markup is below. Please do not edit.]

Reviewers' comments:

Reviewer's Responses to Questions

**Comments to the Author**

Reviewer #1: All comments have been addressed

Reviewer #2: All comments have been addressed

Reviewer #3: All comments have been addressed

Reviewer #4: (No Response)

**********

2. Is the manuscript technically sound, and do the data support the conclusions?

Reviewer #1: Yes

Reviewer #2: Yes

Reviewer #3: Yes

Reviewer #4: Yes

**********

3. Has the statistical analysis been performed appropriately and rigorously?

Reviewer #1: Yes

Reviewer #2: Yes

Reviewer #3: Yes

Reviewer #4: Yes

**********

4. Have the authors made all data underlying the findings in their manuscript fully available?

The PLOS Data policy

Reviewer #1: Yes

Reviewer #2: Yes

Reviewer #3: Yes

Reviewer #4: Yes

**********

5. Is the manuscript presented in an intelligible fashion and written in standard English?

Reviewer #1: Yes

Reviewer #2: Yes

Reviewer #3: Yes

Reviewer #4: Yes

**********

Reviewer #1: (No Response)

Reviewer #2: I would like to thank the Authors for having considered my suggestions

From my point of view, the manuscript deserves publication.

Reviewer #3: (No Response)

Reviewer #4: The manuscript proposes an improved SVD-based noise separation method to address the problem of residual anthropogenic noise in magnetotelluric (MT) signal denoising using traditional singular value decomposition (SVD). The topic is meaningful, and the manuscript has been well revised according to previous review comments. However, several details still need further improvement. Specific suggestions are as follows:

1. The revised introduction is generally acceptable. However, since this paper mainly proposes an improved SVD method that combines SVD with CEEMD, the authors should further clarify in the introduction the advantages and improvements of the proposed method compared with existing studies (e.g., doi.org/10.1190/geo2024-0066.1, etc.), both in theoretical and application aspects. This would help to better highlight the innovation and necessity of the proposed approach compared with other improved methods, such as K-SVD and ICEEMDAN.

2. The formatting of some equations is inconsistent. For instance, ellipses are missing in some expressions, and equation numbers should be uniformly right-aligned. It is recommended to thoroughly check and unify the equation formatting throughout the manuscript.

3. Some symbols are not rigorously defined. For example, it is unclear whether − and + in Equations (4) and (5) refer to the same parameter. Please clearly define all symbols and ensure the correct use of italic and upright fonts for variables and constants to meet academic standards.

4. In the simulation experiments, readers expect to see quantitative metrics rather than only graphical comparisons. It is suggested to add corresponding numerical values above the rectangular regions in Figures 7 and 8 to enhance the persuasiveness of the results.

5. The earlier sections mainly compare the traditional SVD, CEEMD, and the proposed method, but Figures 14 and 15 only present comparisons with the Robust method. It is recommended to include the results of traditional SVD and CEEMD to more comprehensively demonstrate the advantages of the improved method.

6. The conclusion section is currently too brief. It is suggested to summarize the algorithm’s performance from both quantitative and qualitative perspectives using simulation and field data, highlighting the improvements of the proposed method in terms of noise suppression effectiveness and signal fidelity.

**********

what does this mean?). If published, this will include your full peer review and any attached files.

If you choose “no”, your identity will remain anonymous but your review may still be made public.

**Do you want your identity to be public for this peer review?**  For information about this choice, including consent withdrawal, please see our Privacy Policy

Reviewer #1: No

Reviewer #2: No

Reviewer #3: No

Reviewer #4: No

**********

[NOTE: If reviewer comments were submitted as an attachment file, they will be attached to this email and accessible via the submission site. Please log into your account, locate the manuscript record, and check for the action link "View Attachments". If this link does not appear, there are no attachment files.]

To ensure your figures meet our technical requirements, please review our figure guidelines: https://journals.plos.org/plosone/s/figures

You may also use PLOS’s free figure tool, NAAS, to help you prepare publication quality figures: https://journals.plos.org/plosone/s/figures#loc-tools-for-figure-preparation.

NAAS will assess whether your figures meet our technical requirements by comparing each figure against our figure specifications.

# Author comment: Author response to Decision Letter 2

**DOI:** 10.1371/journal.pone.0334468.r004

12 Jun 2026

Manuscript ID: PONE-D-25-11846

Manuscript Title: Research on magnetotelluric signal noise separation based on improved SVD Decomposition

Dear Dr Brian Patrick Weaver:

We greatly appreciate your letter regarding our manuscript (ID: PONE-D-25-11846) entitled “Research on magnetotelluric signal noise separation based on improved SVD Decomposition”. We are also extremely grateful for the reviewers’ constructive comments. We have carefully considered every comment and made thorough revisions accordingly.

To facilitate the evaluation process for you and the reviewers, all modifications have been highlighted in red in the revised manuscript. Furthermore, based on their valuable suggestions, we have provided detailed, point-by-point responses to each question in the accompanying document.

We sincerely hope that our revisions meet your expectations. Should you have any further questions regarding this manuscript, please do not hesitate to contact us at your earliest convenience.

Most sincerely,

Fuming Qu

Jun 13, 2026

Dear Reviewers #1, #2, and #3:

We would like to express our sincere gratitude to you for your time and effort in evaluating our manuscript. We are deeply honored by your positive assessments and for recommending our work for publication. Your recognition of the significance and value of our research is highly encouraging to our entire team. Thank you for your support.

Most sincerely,

Fuming Qu

Reviewer # 4

The manuscript proposes an improved SVD-based noise separation method to address the problem of residual anthropogenic noise in magnetotelluric (MT) signal denoising using traditional singular value decomposition (SVD). The topic is meaningful, and the manuscript has been well revised according to previous review comments. However, several details still need further improvement.

Dear Reviewer #4:

Thank you very much for your insightful comments and suggestions regarding our manuscript. We are deeply grateful for your recognition of our work. We found your feedback to be highly constructive, and we have done our best to fully address your remaining concerns. Please find the revised parts throughout the manuscript highlighted in red. We hope these revisions meet with your approval for publication.

Most sincerely,

Fuming Qu

The following are our point-by-point responses to your comments:

Comments:

Comment[1] The revised introduction is generally acceptable. However, since this paper mainly proposes an improved SVD method that combines SVD with CEEMD, the authors should further clarify in the introduction the advantages and improvements of the proposed method compared with existing studies (e.g., doi.org/10.1190/geo2024-0066.1, etc.), both in theoretical and application aspects. This would help to better highlight the innovation and necessity of the proposed approach compared with other improved methods, such as K-SVD and ICEEMDAN

Response: Thank you for your valuable and insightful comments. We fully agree that clarifying the advantages of the proposed method compared to existing advanced studies is crucial for highlighting its innovation and necessity.

In the revised manuscript, we have expanded the introduction to explicitly discuss the advantages of our SVD-CEEMD approach over methods like deep learning (e.g., CS-ResNet; doi.org/10.1190/geo2024-0066.1), K-SVD, and ICEEMDAN. Specifically, we summarized its theoretical advantage as a data-driven, unsupervised approach free from massive prior data dependencies, and its practical advantage as a two-stage mechanism that effectively resolves boundary artifacts and edge suppression issues in severe interference environments.

The revised parts in the manuscript are marked in red, and the references have been updated accordingly. The specific added content is as follows:

Despite powerful noise suppression capabilities, emerging deep learning frameworks (e.g., CS-ResNet [32]) demand massive, uncontaminated training datasets and high computational loads, severely limiting their field applicability in high-interference mining zones. Meanwhile, dictionary learning methods like K-SVD [33] rely heavily on pre-trained dictionaries, struggling to efficiently adapt to unpredictable, non-stationary impulsive noises. Additionally, while advanced mode decomposition algorithms like ICEEMDAN [4,34] effectively mitigate mode mixing, they fail to independently resolve the high-energy boundary mutations characteristic of large-scale noise in severe interference environments.

To overcome these limitations, this study proposes a data-driven, unsupervised hybrid method combining SVD with complementary ensemble empirical mode decomposition (CEEMD). Theoretically, it circumvents the high computational costs and prior data dependencies inherent in dictionary and deep learning methods. Practically, it implements an efficient two-stage mechanism: SVD primarily isolates high-energy noise based on amplitude discrepancies, while CEEMD secondarily eliminates the residual impulsive noise. This joint strategy resolves both the boundary artifacts of traditional SVD and the edge suppression issues of standalone CEEMD, yielding superior signal-to-noise separation and computational efficiency.

Comment[2]The formatting of some equations is inconsistent. For instance, ellipses are missing in some expressions, and equation numbers should be uniformly right-aligned. It is recommended to thoroughly check and unify the equation formatting throughout the manuscript.

Response: We sincerely apologize for the formatting inconsistencies in our initial submission, and we greatly appreciate your review. Your attention to detail has significantly improved the professional presentation and readability of our manuscript.

Per your recommendation, we have conducted a thorough and comprehensive review of all mathematical equations, inline variables, and symbols throughout the text. Specifically, we have implemented the following formatting unifications:

(1)We have reformatted all standalone equations using the standard equation editor to ensure that every equation number is uniformly right-aligned.

(2)We have added standard mathematical ellipses where they were missing in sequence definitions (e.g., Equation 2) to strictly adhere to mathematical typesetting standards.

We have unified the equation formatting throughout the entire manuscript. All modified equations and mathematical expressions have been highlighted in red in the revised manuscript.

Y=X+N=Us…UnSs⋯0⋮⋱⋮0⋯SnVsT⋮VnT#(2)

Comment[3]Some symbols are not rigorously defined. For example, it is unclear whether − and + in Equations (4) and (5) refer to the same parameter. Please clearly define all symbols and ensure the correct use of italic and upright fonts for variables and constants to meet academic standards.

Response: Thank you very much for this careful and valuable suggestion. We completely agree that rigorous symbol definitions and correct mathematical formatting are crucial for academic clarity.

To address the ambiguity regarding the plus/minus signs in the former Equations (4) and (5), we have consolidated them into a single, more concise equation, which inherently avoids the previous sign confusion while fully preserving the underlying logic. A detailed explanation has been supplemented right below the new equation.

Furthermore, we have conducted a thorough review of the entire manuscript to ensure that all symbols are rigorously and clearly defined. We have also strictly unified the use of italic fonts for mathematical variables and upright fonts for constants, operators, and descriptive subscripts in accordance with academic publishing standards. All the modified equations and text have been highlighted in red in the revised manuscript.

The revised parts in the manuscript are marked in red, and the specific added content is as follows:

xi=x+ε1E1wi#(4)

In which, w(i) represents the ith group of Gaussian white noise, where i=1,2,3,…,I, and E1() denotes the first-order component produced by EMD decomposition. ε1 represents the weight coefficient of the noise component E1(w(i)) (its value is defined as the ratio of the signal-to-noise ratio of the added noise to the standard deviation of this noise component). Based on equation (4), the sequence x(i) is obtained to derive the first-order IMF component.

Comment[4] In the simulation experiments, readers expect to see quantitative metrics rather than only graphical comparisons. It is suggested to add corresponding numerical values above the rectangular regions in Figures 7 and 8 to enhance the persuasiveness of the result.

Response: Thank you for your constructive suggestion. We fully agree that providing quantitative metrics is essential for intuitively and objectively demonstrating the effectiveness of the denoising methods.

We have added the corresponding numerical evaluation metrics directly above the rectangular regions in both Figure 7 and Figure 8. Furthermore, to provide a more comprehensive analysis, we have expanded the discussion in the revised manuscript.

The revised parts in the manuscript are marked in red, and the specific added content is as follows:

Figure 8 presents the evaluation of the spectral properties of the denoised signals using different methods, corresponding to Figure 7. To quantitatively evaluate the denoising performance, the numerical metrics of the different methods under various noise conditions have been annotated in Figures 7 and 8. These specific values provide a direct and objective comparison of the improvements achieved by each method.

Figure 7. Evaluation of denoised signals after suppressing different noises.

(a), (b), (c), and (d) represent the signal-to-noise ratio (SNR), data error (E), normalized cross-correlation (NCC), and mean square error (MSE) of the data processed by different methods, respectively. The black solid line represents the traditional SVD method, the white dashed line represents the CEEMD method, and the red solid line represents the proposed method. The numerical values above the lines indicate the specific quantitative metrics obtained by each method.

Figure 8. Evaluation of spectral properties before and after denoising.

(a), (b), (c), and (d) correspond to the evaluation metrics shown in Figure 7, respectively. The black solid line represents the traditional SVD method, the white dashed line represents the CEEMD method, and the red solid line represents the proposed method. The numerical values above the lines indicate the specific quantitative metrics obtained by each method.

Comment[5] The earlier sections mainly compare the traditional SVD, CEEMD, and the proposed method, but Figures 14 and 15 only present comparisons with the Robust method. It is recommended to include the results of traditional SVD and CEEMD to more comprehensively demonstrate the advantages of the improved method.

Response: We sincerely appreciate your constructive suggestion. We completely agree that including the results of traditional SVD and CEEMD in the field data analysis provides a much more comprehensive and convincing demonstration of the proposed method's advantages.

We have updated Figures 14 and 15 to include the processing results of the traditional SVD method (Subplot b) and the CEEMD method (Subplot c), alongside the raw data (a), the Robust method (d), and our proposed method (e).

Correspondingly, we have expanded the discussion in the revised manuscript to systematically compare the performance of all these methods. Specifically, we detailed how traditional SVD and CEEMD provide limited improvements and can even introduce severe fluctuations in the phase curves for highly contaminated data (Site 49), whereas the proposed method effectively smooths the curves and enhances interpretability. For the high-quality dataset (Site 48), we also added discussions on the marginal improvements yielded by the baseline methods compared to the smoother results of the proposed method.

The updated figures, captions, and corresponding descriptive text have been highlighted in red in the revised manuscript, and the specific added content is as follows:

Figure 14 shows the apparent resistivity phase curves for Site 49 before and after processing with different methods. Due to the high noise level at this station, many data points are affected by noise, resulting in significant and scattered frequency point jumps in the apparent resistivity-phase curves within the 1-0.001 Hz frequency band before denoising. As shown in Figures 14b and 14c, traditional SVD and CEEMD provide limited improvements to the apparent resistivity curves; in fact, they even introduce more severe fluctuations in the phase curves. The Robust method (Figure 14d) only partially improves the fluctuations of some jump points, but the frequency points remain discrete and fail to form a continuous, smooth curve, indicating relatively weak improvement in phase curve jump points. In contrast, the proposed method (Figure 14e) provides a more robust interpretation of the apparent resistivity-phase curves, making them smoother. However, there are still a few jump points in the phase curve (pyx) between 0.01 and 0.001 Hz. Overall, the proposed method achieves better performance in smoothing the apparent resistivity-phase curves, enhancing the interpretability and robustness.

The apparent resistivity phase curves for Site 48 before and after processing with the different methods are shown in Figure 15. Since the data quality at Site 48 is relatively high and less affected by noise, the analysis of Figure 15 reveals that the trends of the apparent resistivity-phase curves after processing with the Robust method and the proposed method are highly consistent. Regarding the baseline methods, SVD yields marginal improvements to the jump points of the phase curves around 0.1 Hz, while CEEMD only corrects a few jump points in the apparent resistivity curves. Comparing the pyx curve processed by the Robust method with that of the proposed method, the latter contains fewer discrete points and exhibits reduced jump amplitudes. In summary, while the Robust and proposed methods yield similar overall trends for this high-quality dataset, the proposed method provides a smoother phase curve with fewer discrete points and lower amplitude jumps.

Figure 14. Comparison of apparent resistivity-phase curves for Site 49 before and after denoising using different methods.

(a) The apparent resistivity-phase curves of the raw data; (b) Results processed by the traditional SVD method; (c) Results processed by the CEEMD method; (d) Results processed by the Robust method; (e) Results processed by the proposed method.

Figure 15. Comparison of apparent resistivity-phase curves for Site 48 before and after denoising using different methods.

(a) The apparent resistivity-phase curves of the raw data; (b) Results processed by the traditional SVD method; (c) Results processed by the CEEMD method; (d) Results processed by the Robust method; (e) Results processed by the proposed method.

Comment[6] The conclusion section is currently too brief. It is suggested to summarize the algorithm’s performance from both quantitative and qualitative perspectives using simulation and field data, highlighting the improvements of the proposed method in terms of noise suppression effectiveness and signal fidelity.

Response: Thank you for your insightful and constructive suggestion. We fully agree that a comprehensive summary structured from both quantitative and qualitative perspectives is essential to properly reflect the algorithm's performance.

Per your recommendation, we have significantly expanded and completely rewritten the Conclusion section. Specifically, we have comprehensively summarized the method’s performance to highlight the improvements in noise suppression effectiveness and signal fidelity:

(1)Using Simulation Data: From a quantitative perspective, we summarized the algorithm's superiority across key evaluation metrics (SNR, E, NCC, and MSE), noting that reduced time-series errors translated into frequency spectrum improvements of up to two orders of magnitude. From a qualitative perspective, we emphasized the high signal fidelity, demonstrating that the method successfully preserves the original signal's morphology while achieving thorough noise separation.

(2)Using Field Data: Quantitatively, we highlighted the algorithm’s effectiveness in suppressing high-energy noise, correcting artificially increased apparent resistivity amplitudes by approximately one order of magnitude. Qualitatively, we emphasized the restoration of continuous and smooth Cagniard apparent resistivity-phase curves, specifically noting the correction of severe phase distortions (e.g., jump points in the pyx curve) in the low-frequency bands (1 to 0.001 Hz).

We believe the revised conclusion now effectively and systematically demonstrates the necessity and superiority of the proposed SVD-CEEMD method. The revised parts in the manuscript are marked in red, and the specific added content is as follows:

4 Conclusion

This study proposes an improved magnetotelluric (MT) signal-noise separation method that integrates Singular Value Decomposition (SVD) with Complementary Ensemble Empirical Mode Decomposition (CEEMD) to address the limitations of traditional SVD in completely extracting noise components. The performance of the proposed method was comprehensively evaluated through both simulation experiments and MT field data processing, yielding the following conclusions:

In simulation experiments, the proposed method outperformed both the traditional SVD and CEEMD methods across key evaluation metrics, including signal-to-noise ratio (SNR), data error (E), normalized cross-correlation (NCC), and mean square error (MSE). Notably, the reduction of time-series error values translated into frequency spectrum improvements of up to two orders of magnitude. Furthermore, the method achieved more thorough signal-noise separation for both large-scale and impulsive noises, successfully preserving the original signal's morphology and ensuring high spectral consistency with the effective signal.

In field data processing, when applied to heavily contaminated stations (e.g., in the Qilian region), the method demonstrated robust practical applicability. It effectively suppressed high-energy noise that had artificially increased the apparent resistivity amplitudes by approximately one order of magnitude. Consequently, the method successfully restored continuous and smooth Cagniard apparent resistivity-phase curves. It demonstrated a particular strength in correcting severe phase curve distortions (such as jump points in the pyx curve) in the low-frequency bands, specifically between 1 and 0.001 Hz. Compared to traditional robust methods that left residual discrete outliers, the proposed approach provided a significantly more reliable interpretation.

As to limitations and future work, similar to other current denoising methods, the proposed method may still result in some loss of effective signals. Furthermore, since large-scale noise and abrupt noise do not always co-occur in real data, applying this dual-stage method to suppress both types simultaneously can occasionally lead to over-processing issues. In future research, we plan to combine neural networks with the proposed framework to intelligently address these problems and optimize the separation process.

Thank you again for your time and efforts to review our paper.

Attachment Submitted filename: Response to Reviewers-R2.docx

# Peer review: Decision Letter 2

**DOI:** 10.1371/journal.pone.0334468.r005

**Authors:** Feier Chen

30 Jun 2026

Dear Dr. YUE,

Thank you for submitting your manuscript to PLOS ONE. After careful consideration, we feel that it has merit but does not fully meet PLOS ONE’s publication criteria as it currently stands. Therefore, we invite you to submit a revised version of the manuscript that addresses the points raised during the review process.

==============================

insufficient justification of the experimental design

==============================

Please submit your revised manuscript by Aug 14 2026 11:59PM. If you will need more time than this to complete your revisions, please reply to this message or contact the journal office at plosone@plos.org. When you're ready to submit your revision, log on to https://www.editorialmanager.com/pone/ and select the 'Submissions Needing Revision' folder to locate your manuscript file.

- A letter that responds to each point raised by the academic editor and reviewer(s). You should upload this letter as a separate file labeled 'Response to Reviewers'.
- A marked-up copy of your manuscript that highlights changes made to the original version. You should upload this as a separate file labeled 'Revised Manuscript with Track Changes'.
- An unmarked version of your revised paper without tracked changes. You should upload this as a separate file labeled 'Manuscript'.

If you would like to make changes to your financial disclosure, please include your updated statement in your cover letter. Guidelines for resubmitting your figure files are available below the reviewer comments at the end of this letter.

If applicable, we recommend that you deposit your laboratory protocols in protocols.io to enhance the reproducibility of your results. Protocols.io assigns your protocol its own identifier (DOI) so that it can be cited independently in the future. For instructions see: https://journals.plos.org/plosone/s/submission-guidelines#loc-laboratory-protocols. Additionally, PLOS ONE offers an option for publishing peer-reviewed Lab Protocol articles, which describe protocols hosted on protocols.io. Read more information on sharing protocols at https://plos.org/protocols?utm_medium=editorial-email&utm_source=authorletters&utm_campaign=protocols.

As the corresponding author, your ORCID iD is verified in the submission system and will appear in the published article. PLOS supports the use of ORCID, and we encourage all coauthors to register for an ORCID iD and use it as well. Please encourage your coauthors to verify their ORCID iD within the submission system before final acceptance, as unverified ORCID iDs will not appear in the published article. *Only*  the individual author can complete the verification step; PLOS staff *cannot*  verify ORCID iDs on behalf of authors.

We look forward to receiving your revised manuscript.

Kind regards,

Feier Chen, Ph.D

Academic Editor

PLOS One

Journal Requirements:

If the reviewer comments include a recommendation to cite specific previously published works, please review and evaluate these publications to determine whether they are relevant and should be cited. There is no requirement to cite these works unless the editor has indicated otherwise.

[Note: HTML markup is below. Please do not edit.]

Reviewers' comments:

Reviewer's Responses to Questions

**Comments to the Author**

Reviewer #1: All comments have been addressed

Reviewer #2: All comments have been addressed

Reviewer #3: All comments have been addressed

Reviewer #4: (No Response)

Reviewer #5: All comments have been addressed

**********

2. Is the manuscript technically sound, and do the data support the conclusions?

Reviewer #1: Yes

Reviewer #2: Yes

Reviewer #3: Yes

Reviewer #4: (No Response)

Reviewer #5: Yes

**********

3. Has the statistical analysis been performed appropriately and rigorously?

Reviewer #1: Yes

Reviewer #2: Yes

Reviewer #3: Yes

Reviewer #4: (No Response)

Reviewer #5: Yes

**********

4. Have the authors made all data underlying the findings in their manuscript fully available?

The PLOS Data policy

Reviewer #1: Yes

Reviewer #2: Yes

Reviewer #3: Yes

Reviewer #4: (No Response)

Reviewer #5: Yes

**********

5. Is the manuscript presented in an intelligible fashion and written in standard English?

Reviewer #1: Yes

Reviewer #2: Yes

Reviewer #3: Yes

Reviewer #4: (No Response)

Reviewer #5: Yes

**********

Reviewer #1: (No Response)

Reviewer #2: I would like to thank the authors for their careful revision of the manuscript and for the responses provided to my comments.

Best regards

Reviewer #3: The paper by Yue et al. addresses a critical challenge in magnetotelluric (MT) data processing effective suppression of human-induced electromagnetic noise, which increasingly hampers accurate impedance estimation. The authors propose an enhanced signal-to-noise separation method by integrating Singular Value Decomposition (SVD) with Complementary Ensemble Empirical Mode Decomposition (CEEMD). By demonstrating improved performance on both synthetic and real datasets, the study contributes to a potentially valuable tool for enhancing the reliability of MT interpretations in noisy environments. I appreciate the great piece of work by the authors in this study. I am happy to accept this manuscript for publication.

Reviewer #4: The manuscript proposes a magnetotelluric (MT) signal noise separation method based on an improved Singular Value Decomposition (SVD) framework. The proposed approach integrates SVD and CEEMD techniques and is presented as a data-driven, unsupervised hybrid denoising framework. The authors claim that the method can achieve effective noise separation while avoiding the high computational cost and prior-data dependence commonly associated with dictionary learning and deep learning approaches. If the claims made in the manuscript can be sufficiently validated, the proposed method may have certain research significance for MT data processing.

Although the authors have revised and supplemented the manuscript in response to the previous review comments, and some issues have been improved, the current version still contains numerous detailed errors, insufficient justification of the experimental design, and inadequately rigorous logical presentation. In addition, some conclusions are not yet supported by sufficient evidence, which undermines the credibility and persuasiveness of the study. Therefore, the authors are encouraged to conduct a comprehensive review and substantial revision of the manuscript, further strengthening the theoretical analysis, experimental validation, and overall presentation to ensure that the quality of the paper meets the publication standards.

1. Abnormal characters, such as square symbols (□), appear in Equations (2) and (13), suggesting possible formula rendering errors, encoding issues, or missing mathematical symbols. The authors are advised to thoroughly check the formatting and encoding of all equations throughout the manuscript to ensure that mathematical expressions are displayed accurately and completely, thereby avoiding potential confusion for readers.

2. The manuscript contains numerous spelling and language errors. For example, "Impluse noise" should be corrected to "Impulse noise", and "niose" should be corrected to "noise". These issues are not isolated cases but appear throughout the manuscript, indicating that the language proofreading remains insufficient. The authors are strongly encouraged to conduct a thorough language review and professional editing of the entire manuscript to improve its overall quality, readability, and adherence to academic writing standards.

3. Key evaluation metrics such as SNR and MSE are standard abbreviations and, according to common academic writing conventions, should be presented in upright type rather than italics. The authors are also encouraged to ensure consistent formatting throughout the manuscript for variables, functions, and evaluation metrics, thereby maintaining uniformity and clarity in the presentation of mathematical symbols and notations.

4. In the manuscript, the CEEMD method adopts an ensemble number of (I = 100). However, the authors do not provide any justification for this parameter choice or discuss its influence on the MT data processing results. Since both the decomposition performance and computational cost of CEEMD are affected by the ensemble number, the authors are encouraged to conduct a parameter sensitivity analysis. Specifically, it would be beneficial to evaluate the performance of the proposed method under different noise levels and ensemble numbers, thereby demonstrating the rationality of the selected parameter setting and assessing the robustness of the method with respect to parameter variations.

5. In the definitions of key evaluation metrics such as SNR and MSE, the physical meanings of (x(n)) and (y(n)) are not described with sufficient clarity. In particular, for denoising applications, it should be explicitly stated whether these variables represent the noisy signal, the reference clean signal, or the denoised signal. The current presentation may lead to ambiguity and makes it difficult for readers to clearly understand how these evaluation metrics are computed. The authors are therefore encouraged to provide the necessary explanations near the corresponding equations and to ensure consistent definitions of all related symbols throughout the manuscript.

6. Figures 12 and 13 present the time-domain results of Sites 48 and 49 processed using conventional SVD, CEEMD, and the proposed method. However, the original signal is only shown in Fig. 11. As a result, readers need to switch between multiple figures to perform comparative analysis, which negatively affects the readability of the manuscript. In addition, the current figures do not display the original signal together with the results of different methods, making it difficult to visually assess the level of noise reduction and signal fidelity. Moreover, the presented time windows are relatively short, the number of sampling points is limited, and only a single channel is provided. Considering that MT data typically include multiple channels such as EX, EY, HX, and HY, and that effective electromagnetic responses exhibit strong inter-channel coherence while random noise generally does not, presenting only single-channel waveforms is insufficient to fully demonstrate the effectiveness of the proposed method in signal recovery. The authors are encouraged to include comparative results for multiple channels (EX, EY, HX, and HY) and to further compute quantitative metrics such as coherence coefficients between channels before and after denoising, in order to evaluate the ability of the proposed method to suppress noise while preserving inter-channel signal consistency. Furthermore, it is recommended to present the original signal, conventional methods, and the proposed method within the same figure for direct comparison, and to include zoomed-in views, error analyses, or frequency-domain comparisons where appropriate, so as to more intuitively and comprehensively demonstrate the advantages of the proposed method in terms of noise suppression and signal fidelity.

7. The image resolution of Figures 14 and 15 is relatively low, and some details are difficult to discern, which affects the analysis of the results. The authors are advised to improve the resolution of the figures and optimize the font size, axis labels, and legend presentation to ensure that all key information is clearly and effectively displayed.

8. In the introduction, the proposed SVD-CEEMD method is explicitly positioned as a data-driven and unsupervised signal processing framework, with the claim that it can avoid the reliance on large-scale prior data and the high computational cost associated with dictionary learning and deep learning methods, which is presented as one of its key advantages. However, in the conclusion section, the authors further state that neural networks may be incorporated in future work to address over-processing issues and improve signal separation performance. This reveals a certain inconsistency in terms of research positioning and methodological direction. If the authors consider that traditional signal processing methods are already sufficient to effectively overcome the limitations of deep learning approaches in MT signal denoising, further clarification is needed regarding the necessity and specific role of introducing neural networks in future work. Conversely, if neural networks still play an indispensable role in improving denoising performance or addressing key challenges, the relationship between the proposed method and deep learning approaches should be described more objectively, rather than emphasizing the "avoidance of deep learning limitations "as a primary advantage in the introduction. The authors are encouraged to carefully reconsider and clarify the positioning of the proposed method, explicitly defining whether its relationship with deep learning methods is one of replacement, complementarity, or staged improvement, so as to enhance the logical consistency and academic rigor of the manuscript.

9. The manuscript repeatedly emphasizes that the proposed method has advantages such as low computational cost, no requirement for prior training data, and a simple processing workflow. However, a systematic comparison with representative traditional MT signal denoising methods is currently missing. In particular, widely used approaches in electromagnetic signal denoising, such as wavelet transform and the Hilbert–Huang transform, have not been included for comparison. As a result, no quantitative evidence is provided to demonstrate how the proposed method performs relative to these classical techniques in terms of denoising effectiveness, computational efficiency, parameter dependency, and applicability. Therefore, the claims regarding the advantages of the proposed method are not yet sufficiently supported by experimental evidence. The authors are encouraged to include comparative experiments with typical traditional denoising methods and to conduct a comprehensive evaluation from multiple perspectives, including SNR improvement, MSE, runtime, algorithmic complexity, and parameter sensitivity. Such an expanded analysis would provide a more objective and comprehensive validation of the proposed method's performance advantages and strengthen the credibility and persuasiveness of the conclusions.

Reviewer #5: The authors propose an enhanced signal-to-noise separation method by integrating Singular Value Decomposition (SVD) with Complementary Ensemble Empirical Mode Decomposition (CEEMD). This hybrid approach leverages the amplitude contrast between weak natural MT signals and dominant anthropogenic noise, aiming to overcome the limitations of traditional SVD methods that often leave residual noise artifacts. By demonstrating improved performance on both synthetic and real datasets, the study contributes to a potentially valuable tool for enhancing the reliability of MT interpretations in noisy environments. I appreciate the great piece of work by the authors in this study. I am happy to accept this manuscript.

**********

what does this mean?). If published, this will include your full peer review and any attached files.

If you choose “no”, your identity will remain anonymous but your review may still be made public.

**Do you want your identity to be public for this peer review?**  For information about this choice, including consent withdrawal, please see our Privacy Policy

Reviewer #1: No

Reviewer #2: No

Reviewer #3: No

Reviewer #4: No

Reviewer #5: No

**********

[NOTE: If reviewer comments were submitted as an attachment file, they will be attached to this email and accessible via the submission site. Please log into your account, locate the manuscript record, and check for the action link "View Attachments". If this link does not appear, there are no attachment files.]

To ensure your figures meet our technical requirements, please review our figure guidelines: https://journals.plos.org/plosone/s/figures

You may also use PLOS’s free figure tool, NAAS, to help you prepare publication quality figures: https://journals.plos.org/plosone/s/figures#loc-tools-for-figure-preparation.

NAAS will assess whether your figures meet our technical requirements by comparing each figure against our figure specifications.

# Author comment: Author response to Decision Letter 3

**DOI:** 10.1371/journal.pone.0334468.r006

14 Aug 2026

Manuscript ID: PONE-D-25-11846R3

Manuscript Title: Research on magnetotelluric signal noise separation based on improved SVD

Dear Dr Feier Chen:

We greatly appreciate your letter regarding our manuscript (ID: PONE-D-25-11846R2) entitled “Research on magnetotelluric signal noise separation based on improved SVD”. We are also extremely grateful for the constructive comments provided by you and the reviewers. We have carefully considered every comment and made thorough revisions accordingly.

To facilitate the evaluation process for you and the reviewers, all modifications have been highlighted in red in the revised manuscript. Furthermore, based on their valuable suggestions, we have provided detailed, point-by-point responses to each question in the accompanying document.

We sincerely hope that our revisions meet your expectations and align with the rigorous academic standards of PLOS ONE. Should you have any further questions regarding this manuscript, please do not hesitate to contact us at your earliest convenience.

Most sincerely,

Fuming Qu

July 3, 2026

Dear Reviewers #1, #2, #3 and #5:

We would like to express our sincere gratitude to you for your time, effort, and rigorous evaluation of our manuscript throughout the review process.

We are deeply honored by your positive assessments and are thrilled that you found the integration of SVD and CEEMD to be a valuable contribution to enhancing the reliability of magnetotelluric (MT) interpretations in noisy environments. Your recognition of the significance of our research—particularly its demonstrated effectiveness on both synthetic and real datasets—is highly encouraging to our entire team.

Thank you again for your invaluable support, your previous constructive feedback that helped shape this manuscript, and for recommending our work for publication.

Most sincerely,

Fuming Qu

Dear Reviewer #4:

We sincerely thank you for your rigorous evaluation and highly constructive comments regarding our manuscript. We also deeply appreciate your acknowledgement of the potential research significance of our proposed SVD-CEEMD method.

You rightly pointed out that our previous version contained detailed errors, lacked sufficient justification for the experimental design, and required a more rigorous logical presentation and stronger evidentiary support. We took your criticisms very seriously. Guided by your insightful suggestions, we have conducted a comprehensive review and a substantial revision of the entire manuscript.

Specifically, we have meticulously corrected the detailed formatting and language errors, significantly strengthened the justification and theoretical analysis of our experimental design, and rigorously refined our logical presentation to ensure that all conclusions are firmly supported by robust evidence. We believe that your strict and professional feedback has profoundly improved the credibility, persuasiveness, and overall quality of our paper.

To facilitate your review, all modifications have been clearly highlighted in red in the revised manuscript. We genuinely hope that these comprehensive revisions fully address your concerns and meet the high publication standards of the journal.

Most sincerely,

Fuming Qu

The following are our detailed, point-by-point responses to your comments:

Comments:

[1]Abnormal characters, such as square symbols (□), appear in Equations (2) and (13), suggesting possible formula rendering errors, encoding issues, or missing mathematical symbols. The authors are advised to thoroughly check the formatting and encoding of all equations throughout the manuscript to ensure that mathematical expressions are displayed accurately and completely, thereby avoiding potential confusion for readers.

Response: We sincerely thank you for bringing this formatting issue to our attention, and we apologize for any confusion it may have caused during the reading process.

The issue was caused by a system conversion error. Originally, we used the built-in Equation Editor in Microsoft Word to insert these formulas. While all mathematical symbols and equations displayed perfectly in our local Word document, an encoding mismatch occurred when the submission system automatically converted the Word file into a PDF. This conversion failure caused certain mathematical symbols to render as abnormal square characters (□).

To resolve this issue completely, we have meticulously re-entered and reformatted Equations, along with thoroughly checking all other mathematical expressions and variables throughout the manuscript. After uploading the revised manuscript, we rebuilt the PDF in the submission system and repeatedly verified the final generated file. We can now confirm that all formulas, mathematical symbols, and encodings are displayed accurately and completely in the system-generated PDF.

We greatly appreciate your rigorous check and patience regarding this technical issue.

[2]The manuscript contains numerous spelling and language errors. For example, "Impluse noise" should be corrected to "Impulse noise", and "niose" should be corrected to "noise". These issues are not isolated cases but appear throughout the manuscript, indicating that the language proofreading remains insufficient. The authors are strongly encouraged to conduct a thorough language review and professional editing of the entire manuscript to improve its overall quality, readability, and adherence to academic writing standards.

Response: We sincerely thank you for carefully reading our manuscript and pointing out these spelling and language errors. We apologize for the insufficient proofreading in our initial submission.

Following your suggestion, we have corrected all instances of “Impluse noise” to “Impulse noise” and “niose” to “noise” throughout the text. Beyond these specific corrections, we have taken your advice very seriously and conducted a comprehensive, word-by-word language review of the entire manuscript. Every single sentence has been meticulously checked for grammatical correctness, and the overall language has been extensively polished to improve readability, clarity, and adherence to academic writing standards.

Because these linguistic refinements and spelling corrections are numerous and distributed throughout the entire text, we have not listed every single minor change in this response letter. Instead, all language, spelling, and grammatical modifications have been clearly highlighted in red in the revised manuscript for your convenience.

We sincerely appreciate your rigorous evaluation, and we hope that our extensive proofreading and language improvements now fully meet your expectations and the high standards of the journal.

[3]Key evaluation metrics such as SNR and MSE are standard abbreviations and, according to common academic writing conventions, should be presented in upright type rather than italics. The authors are also encouraged to ensure consistent formatting throughout the manuscript for variables, functions, and evaluation metrics, thereby maintaining uniformity and clarity in the presentation of mathematical symbols and notations.

Response: We sincerely thank you for this highly professional and meticulous observation.

Following your valuable suggestion, we have corrected the formatting of the evaluation metrics (including SNR, MSE, NCC, and E), changing them all to upright type throughout the manuscript and equations.

Furthermore, we have conducted a comprehensive and systematic review of all mathematical formulas, symbols, and notations across the entire manuscript to ensure strict adherence to standard academic formatting conventions. Specifically, we have standardized the formatting according to the following rules:

(1)Upright Type: We have ensured that all standard abbreviations (e.g., SNR, MSE, SVD, CEEMD, IMF), established evaluation metrics, standard mathematical operators/functions (e.g., max, min, log, and the mean(·)operator), and descriptive subscripts/superscripts (e.g., the subscript 's' for signal and 'n' for noise in Us and Un, or T for transpose) are consistently presented in upright type.

(2)Italics: We have ensured that all physical quantities, continuous and discrete scalar variables (e.g., x, y, N, K), and iteration indices (e.g., i, k, n) are strictly presented in italics.

All these formatting corrections and standardizations have been clearly highlighted in red in the revised manuscript. We believe that these extensive revisions have significantly enhanced the uniformity, readability, and overall professional presentation of our mathematical notations. We hope these changes fully meet your expectations.

[4]In the manuscript, the CEEMD method adopts an ensemble number of (I = 100). However, the authors do not provide any justification for this parameter choice or discuss its influence on the MT data processing results. Since both the decomposition performance and computational cost of CEEMD are affected by the ensemble number, the authors are encouraged to conduct a parameter sensitivity analysis. Specifically, it would be beneficial to evaluate the performance of the proposed method under different noise levels and ensemble numbers, thereby demonstrating the rationality of the selected parameter setting and assessing the robustness of the method with respect to parameter variations

Response: Thank you very much for this valuable and constructive comment. We agree that the original manuscript did not provide sufficient justification for selecting the CEEMD ensemble number (I = 100), nor did it adequately discuss the influence of this parameter on denoising performance and computational cost.

In response, we conducted an additional parameter sensitivity analysis using the same synthetic mixed-noise signal and signal-reconstruction criterion as those employed in the simulation experiment. In accordance with the definition used in the manuscript, I denotes the number of Gaussian white-noise realizations used in the CEEMD ensemble. Four representative ensemble numbers (I = 20, 100, 200, and 500) were evaluated under two input noise levels corresponding to SNRs of 0 dB and −3 dB. During the analysis, the standard deviation of the Gaussian white noise added by CEEMD was fixed at 0.2 times that of the noisy input signal, while all other parameters were held constant.

For each combination of input SNR and ensemble number, the CEEMD decomposition was independently repeated three times using independently generated Gaussian white-noise ensembles while retaining the same input noisy signal. Output SNR, mean squared error (MSE), normalized cross-correlation (NCC), and computational runtime were evaluated. The reported markers represent the mean values of the three runs, and the error bars indicate ±1 standard deviation.

The results show that increasing I from 20 to 100 generally improves the denoising performance under both tested noise levels. When I is further increased to 200 or 500, only relatively small variations are observed, and no consistent additional improvement is obtained across all evaluation metrics and noise levels. By contrast, the computational runtime continues to increase substantially with I, particularly at I = 500. These results indicate that the performance of the proposed method tends to stabilize at approximately I = 100 under the tested conditions. Therefore, I = 100 was selected as a reasonable compromise between denoising performance and computational efficiency.

The complete parameter sensitivity analysis, including the experimental settings, quantitative results, explanatory discussion, and figure caption, has been added as S1 Appendix. The corresponding description in the main manuscript has also been revised to state that I = 100 was selected based on the parameter sensitivity analysis presented in S1 Appendix.

The revised portions of the manuscript are marked in red.

Section 1.2:

First, a single-channel MT signal to be analyzed is denoted by x. A total of I Gaussian white-noise realizations are added to the signal, where I is set to 100 based on the parameter sensitivity analysis presented in S1 Appendix. The resulting noise-assisted sequences are constructed as follows:

S1 Appendix. Sensitivity analysis of the CEEMD ensemble number

To evaluate the sensitivity of the proposed method to the CEEMD ensemble number I, the same synthetic mixed-noise signal and signal-reconstruction criterion as those used in the simulation experiment were adopted. Four representative ensemble numbers (I = 20, 100, 200, and 500) were evaluated under input SNRs of 0 dB and −3 dB. During the sensitivity analysis, the standard deviation of the noise added by CEEMD was fixed at 0.2 times that of the noisy input signal, and all parameters other than I were held constant. Here, I denotes the number of Gaussian white-noise realizations used in the CEEMD ensemble. For each parameter combination, the CEEMD decomposition was repeated three times using independent realizations of the added noise while retaining the same input noisy signal. Output SNR, mean squared error (MSE), normalized cross-correlation (NCC), and computational runtime were used as evaluation metrics. The markers in Fig. A1 indicate the mean values of the three runs, and the error bars indicate ±1 standard deviation (SD).

As shown in Fig. A1, increasing I from 20 to 100 generally improves the denoising performance under both tested input SNR levels, as indicated by the increase in output SNR, decrease in MSE, and improvement in NCC. When I is further increased to 200 or 500, only relatively small variations are observed, and no consistent additional improvement is obtained across all metrics and noise levels. This indicates that the denoising performance tends to stabilize at approximately I = 100. In contrast, the computational runtime increases continuously with I, with a particularly substantial increase at I= 500.

Therefore, considering both denoising performance and computational efficiency, I = 100 was considered a reasonable performance–cost compromise among the tested ensemble numbers and was adopted in the proposed method.

Fig. A1. Sensitivity analysis of the CEEMD ensemble number under input SNRs of 0 dB and −3 dB. (a) Output SNR, (b) mean squared error (MSE), (c) normalized cross-correlation (NCC), and (d) computational runtime. Each parameter combination was evaluated using three independent CEEMD runs. Markers indicate the mean values, and error bars indicate ±1 standard deviation (SD).

[5]In the definitions of key evaluation metrics such as SNR and MSE, the physical meanings of (x(n)) and (y(n)) are not described with sufficient clarity. In particular, for denoising applications, it should be explicitly stated whether these variables represent the noisy signal, the reference clean signal, or the denoised signal. The current presentation may lead to ambiguity and makes it difficult for readers to clearly understand how these evaluation metrics are computed. The authors are therefore encouraged to provide the necessary explanations near the corresponding equations and to ensure consistent definitions of all related symbols throughout the manuscript.

Response: Thank you for this highly constructive and rigorous comment. We completely agree that the previous descriptions of x(n) and y(n) were not sufficiently explicit and could cause ambiguity in the context of denoising evaluation.

First, we have revised the explanation near Equations (12)-(15) in Section 2 to explicitly clarify that, in the synthetic experiments, x(n) denotes the reference clean MT signal before adding artificial noise, whereas y(n) denotes the denoised signal output by a given processing method. We also clarified that the artificially noisy signal is used solely as the input to the denoising methods, but not as the reference signal for calculating SNR, NCC, E, and MSE. The discrete sample index n and the total number of samples N have also been explicitly defined.

Furthermore, taking your advice very seriously, we have conducted a comprehensive review and meticulous revision of the entire manuscript. We thoroughly checked every single formula to ensure that the physical meaning of every variable is clearly explained immediately following its corresponding equation. We have standardized all mathematical notations to guarantee absolute consistency and to eliminate any notational conflicts throughout the text.

All these modifications are clearly highlighted in red in the revised manuscript. While numerous minor notational corrections are not listed here to avoid redundancy, two major revisions regarding variable definitions are detailed below:

Line 182-195:

Large-scale noise and complex interferences (such as square waves and impulse noise) are added to the low-noise MT signals. The results of different denoising methods are then compared to verify the effectiveness and robustness of the proposed approach. For the synthetic tests, since the clean reference signal is known prior to the addition of artificial noise, the denoising performance can be quantitatively evaluated by comparing the denoised signal against this reference. The signal-to-noise ratio (SNR), normalized cross-correlation (NCC), fitting error E, and mean squared error (MSE) are used to quantitatively analyze the improvement effects of the processing methods. These parameters are defined as follows [41,42]:

SNR=20lgy(n)2y(n)−x(n)2#(12)

NCC=n=1Ny(n)⋅x(n)n=1Ny2(n)⋅n=1Nx2(n)#(13)

E=y(n)−x(n)2y(n)2#(14)

MSE=n=1Ny(n)−x(n)2N#(15)

where x(n) represents the reference clean MT signal (i.e., the baseline low-noise signal prior to adding synthetic noise), y(n) denotes the denoised signal output by the processing method, and n is the discrete sample index, N is the total number of samples, and ⋅2 denotes the L2 norm of the vector. The artificial noisy signal is used solely as the input for the denoising procedures and is not used as a reference for metric calculation. Therefore, a better denoising effect is characterized by higher SNR and NCC values, corresponding to lower E and MSE values.

Line 123-136:

The time series vectors xi are used to construct a real matrix X=[x1Tx2T…xnT]，where X∈ℝm×n，SVD is then applied to the matrix X [37,38]:

X=USVT#(1)

where X represents the multidimensional input matrix constructed from the original noisy MT signal, and the main diagonal elements of S∈ℝm×n are the singular values of the matrix X. The superscript T denotes the transpose operation,p=min(m,n), and σ1≥σ2≥⋯≥σp≥0 for σi(i=1,2,⋯, p). U is an m×m orthogonal matrix of left singular vectors, and V is an n×n orthogonal matrix of right singular vectors.

In SVD, the input data must be a multidimensional matrix. Therefore, it is necessary to transform the one-dimensional MT time series into a multidimensional matrix using Hankel matrices. Additionally, the components obtained from SVD can be divided into effective signals and noise:

X=Xs+N=[Us⋯Un]Ss⋯0⋮⋱⋮0⋯SnVsT⋮VnT#(2)

where Xs represents the effective signal matrix, and N represents the noise matrix. The subscript s denotes the effective signal components, and the subscript n denotes the noise components in the decomposed singular vectors and values.

[6]Figures 12 and 13 present the time-domain results of Sites 48 and 49 processed using conventional SVD, CEEMD, and the proposed method. However, the original signal is only shown in Fig. 11. As a result, readers need to switch between multiple figures to perform comparative analysis, which negatively affects the readability of the manuscript. In addition, the current figures do not display the original signal together with the results of different methods, making it difficult to visually assess the level of noise reduction and signal fidelity. Moreover, the presented time windows are relatively short, the number of sampling points is limited, and only a single channel is provided. Considering that MT data typically include multiple channels such as EX, EY, HX, and HY, and that effective electromagnetic responses exhibit strong inter-channel coherence while random noise generally does not, presenting only single-channel waveforms is insufficient to fully demonstrate the effectiveness of the proposed method in signal recovery. The authors are encouraged to include comparative results for multiple channels (EX, EY, HX, and HY) and to further compute quantitative metrics such as coherence coefficients between channels before and after denoising, in order to evaluate the ability of the proposed method to suppress noise while preserving inter-channel signal consistency. Furthermore, it is recommended to present the original signal, conventional methods, and the proposed method within the same figure for direct comparison, and to include zoomed-in views, error analyses, or frequency-domain comparisons where appropriate, so as to more intuitively and comprehensively demonstrate the advantages of the proposed method in terms of noise suppression and signal fidelity.

Response: Thank you very much for this valuable and constructive comment. We fully agree that presenting the raw signal together with the results of different denoising methods can facilitate direct comparison, and that multichannel analysis is important for evaluating the applicability of an MT denoising method. Following the your suggestions, we have carefully revised the relevant figures, descriptions, and discussion in the manuscript.

First, Figures 12 and 13 have been revised to display the raw signal together with the corresponding results obtained using conventional SVD, CEEMD, and the proposed method. In each pair of panels, the raw signal and its spectrum are shown in red, while the corresponding denoised results are shown in black. Both time-domain waveforms and frequency-domain spectra are presented in the same figures. The figure captions and the related discussion in Section 3 have also been revised accordingly. These modifications allow readers to directly evaluate the differences in noise suppression and signal preservation among the three methods without switching between Figures 11-13.

Second, to extend the field-data evaluation beyond a single channel, we have added the processing results of the Ex, Ey, Hx, and Hy components at Site 48 obtained using the proposed method. These multichannel results, together with the corresponding explanatory paragraph and figure caption, are now presented as Figure 14 in the revised manuscript. The added results demonstrate the applicability of the proposed method to both electric and magnetic field components and extend the field-data evaluation from a single component to the four routinely recorded MT channels.

We also appreciate the your suggestions concerning the displayed time-window length and the quantitative evaluation of inter-channel coherence. The present figures retain the representative data interval used in the original comparison because this interval contains the characteristic noise waveforms and allows the local differences among the denoising methods to remain clearly visible. We acknowledge, however, that this relatively short interval does not constitute a long-duration multichannel validation.

Furthermore, the newly added four-channel results provide a qualitative demonstration of channel-wise applicability, but they do not constitute a full multichannel comparison among conventional SVD, CEEMD, and the proposed method, nor do they quantitatively establish the preservation of inter-channel coherence. Rather than reporting coherence coefficients that may be sensitive to the selected data interval and spectral parameters, we have identified this issue as an important direction for further investigation.

Accordingly, the Conclusion has been revised to state that future research will analyze longer continuous and synchronized multichannel MT records to evaluate the preservation of electric-magnetic relationships after denoising. Frequency-dependent inter-channel coherence has also been incorporated into the proposed broader evaluation framework.

We sincerely appreciate your comment, which has helped us improve the direct comparability of the figures, broaden the field-data presentation to multiple MT components, and clarify the limitations and future validation requirements of the present study.

All corresponding revisions in the manuscript are marked in red:

Figure 12. Direct comparison of the raw and denoised signals at Site 49 in the time and frequency domains. The raw signal and its spectrum are shown in red, whereas the denoised results are shown in black. Panels (a) and (b) present the results obtained using conventional SVD; panels (c) and (d) present the results obtained using CEEMD; and panels (e) and (f) present the results obtained using the proposed method. Panels (a), (c), and (e) show the time-domain signals, while panels (b), (d), and (f) show the corresponding amplitude spectra.

Figure 13. Direct comparison of the raw and denoised signals at Site 48 in the time and frequency domains. The raw signal and its spectrum are shown in red, whereas the denoised results are shown in black. Panels (a) and (b) present the results obtained using conventional SVD; panels (c) and (d) present the results obtained using CEEMD; and panels (e) and (f) present the results obtained using the proposed method. Panels (a), (c), and (e) show the time-domain signals, while panels (b), (d), and (f) show the corresponding amplitude spectra.

To further examine the applicability of the proposed method to different MT components, the Ex, Ey, Hx, and Hy channels at Site 48 were processed using the proposed method, as shown in Figure 14. The results demonstrate that the proposed processing workflow is applicable to both electric and magnetic field components, thereby extending the field-data evaluation from a single component to the four routinely recorded MT channels.

Figure 14. Multichannel MT time series after denoising using the proposed method at Site 48. (a) Ex, (b) Ey, (c) Hx, and (d) Hy. The four components are displayed using channel-specific vertical scales because of their different physical units and amplitude ranges.

Conclusion:

Furthermore, a broader evaluation framework would help to better contextualize the performance and applicability of the proposed method. The present study focused on comparisons with traditional SVD, standalone CEEMD, and the Robust method, which are directly related to the motivation and workflow of the proposed approach. Future research will extend the benchmark evaluation to include other representative denoising methods, such as wavelet transform, Hilbert–Huang transform, and variational mode decomposition, under different noise types and field conditions. Longer continuous and synchronized multichannel MT records will also be analyzed to evaluate the preservation of electric–magnetic relationships after denoising. Accordingly, evaluation criteria such as SNR improvement, MSE, runtime, parameter sensitivity, practical applicability, and frequency-dependent inter-channel coherence will be considered where appropriate to provide a more systematic assessment of MT denoising strategies.

[7]The image resolution of Figures 14 and 15 is relatively low, and some details are difficult to discern, which affects the analysis of the results. The authors are advised to improve the resolution of the figures and optimize the font size, axis labels, and legend presentation to ensure that all key information is clearly and effectively displayed.

Response: Thank you for this constructive comment. We agree that the resolution and visual clarity of Figures 14 and 15 in the previous version were insufficient, which may have affected the readability of the apparent resistivity and phase curves and the comparison among different denoising methods.

In the revised manuscript, we have replaced Figures 14 and 15 with higher-resolution versions, and the corresponding figure captions have been retained and checked for consistency.

The revised parts in the manuscript have been marked in red.

Figure 14. Comparison of apparent resistivity and phase curves for Site 49 before and after denoising using different methods.

(a)The apparent resistivity and phase curves of the raw data; (b) results processed by the traditional SVD method; (c) results processed by the CEEMD method; (d) results processed by the Robust method; and (e) results processed by the proposed method.

Figure 15. Comparison of apparent resistivity and phase curves for Site 48 before and after denoising using different methods.

(a)The apparent resistivity and phase curves of the raw data; (b) results processed by the traditional SVD method; (c) results processed by the CEEMD method; (d) results processed by the Robust method; and (e) results processed by the proposed method.

[8]In the introduction, the proposed SVD-CEEMD method is explicitly positioned as a data-driven and unsupervised signal processing framework, with the claim that it can avoid the reliance on large-scale prior data and the high computational cost associated with dictionary learning and deep learning methods, which is presented as one of its key advantages. However, in the conclusion section, the authors further state that neural networks may be incorporated in future work to address over-processing issues and improve signal separation performance. This reveals a certain inconsistency in terms of research positioning and methodological direction. If the authors consider that traditional signal processing methods are already sufficient to effectively overcome the limitations of deep learning approaches in MT signal denoising, further clarification is needed regarding the necessity and specific role of introducing neural networks in future work. Conversely, if neural networks still play an indispensable role in improving denoising performance or addressing key challenges, the relationship between the proposed method and deep learning approaches should be described more objectively, rather than emphasizing the "avoidance of deep learning limitations "as a primary advantage in the introduction. The authors are encouraged to carefully reconsider and clarify the positioning of the proposed method, explicitly defining whether its relationship with deep learning methods is one of replacement, complementarity, or staged improvement, so as to enhance the logical consistency and academic rigor of the manuscript.

Response: We sincerely thank the reviewer for this insightful and constructive comment. We completely agree that our previous wording in the Introduction and Conclusion created a logical inconsistency regarding the research positioning of the proposed method.

Following your valuable suggestion, we have carefully reconsidered and explicitly defined the relationship between our SVD-CEEMD method and deep learning approaches as complementary.

Specifically, the proposed SVD-CEEMD method serves as a lightweight, unsupervised, and physically interpretable signal-processing framework that is highly effective under limited prior-data conditions. We do not consider heavy neural networks to be indispensable components of the core signal separation task. Instead, we clarify that future lightweight statistical or feature-based classification approaches could serve a complementary role by providing auxiliary decision information (e.g., noise-type identification) for adaptive workflow control, preventing over-processing without sacrificing computational efficiency.

To reflect this clarified positioning and enhance the academic rigor of the manuscript, we have made the following revisions:

1. Revisions to the Introduction

We revised the text to avoid overemphasizing the “avoidance of deep learning limitations” as an absolute advantage. The revised text now reads:

“The proposed method does not require a pre-trained dictionary or large-scale labeled training datasets, and therefore provides a lightweight and unsupervised alternative for MT denoising under limited prior-data conditions.”

2. Revisions to the Conclusion

We revised the conclusion to remove the broad mention of “neural networks” and to clarify the specific, complementary role of future auxiliary methods. The revised text now reads:

“Regarding the limitations and future development of the proposed method, similar to other current denoising approaches, slight attenuation of the underlying signals may still occur after processing. In addition, large-scale noise and abrupt noise do not always co-occur in real MT data; therefore, applying the complete two-stage processing procedure to all data segments may occasionally lead to over-processing. Future work should focus on adaptive workflow control, including noise-type identification, parameter selection, and the choice between single-stage and two-stage processing. Lightweight statistical or feature-based classification approaches will be explored to provide auxiliary decision information prior to signal reconstruction. This strategy may reduce unnecessary computational overhead while maintaining the interpretability and low-prior-data requirements of the proposed SVD-CEEMD framework.”

We hope that these revisions meet your expectations by explicitly defining the relationship between the proposed method and learning-based approaches, thereby resolving the logical inconsistency in the manuscript.

[9]The manuscript repeatedly emphasizes that the proposed method has advantages such as low computational cost, no requirement for prior training data, and a simple processing workflow. However, a systematic comparison with representative traditional MT signal denoising methods is currently missing. In particular, widely used approaches in electromagnetic signal denoising, such as wavelet transform and the Hilbert–Huang transform, have not been included for comparison. As a result, no quantitative evidence is provided to demonstrate how the proposed method performs relative to these classical techniques in terms of denoising effectiveness, computational efficiency, parameter dependency, and applicability. Therefore, the claims regarding the advantages of the proposed method are not yet sufficiently supported by experimental evidence. The authors are encouraged to include comparative experiments with typical traditional denoising methods and to conduct a comprehensive evaluation from multiple perspectives, including SNR improvement, MSE, runtime, algorithmic complexity, and parameter sensitivity. Such an expanded analysis would provide a more objective and comprehensive validation of the proposed method's performance advantages and strengthen the credibility and persuasiveness of the conclusions.

Response: Thank you very much for this valuable and insightful comment. We fully agree that a broader and more systematic comparison with representative traditional MT denoising methods, such as wavelet transform and Hilbert-Huang transform, would further strengthen the quantitative validation and contextualization of the proposed method.

In response to this comment, we first carefully reviewed the manuscript and revised several expressions that may have sounded overly general or insufficiently constrained. In particular, we made the claims regarding computational efficiency, workflow simplicity, and performance advantages more cautious and more clearly limited to the methods compared in this study. For example, in the Introduction, the original sentence:

“This joint strategy resolves both the boundary artifacts of traditional SVD and the edge suppression issues of standalone CEEMD, yielding superior signal-to-noise separation and computational efficiency.”

has been revised as:

“This joint strategy improves both the boundary artifacts of traditional SVD and the edge suppression issues of standalone CEEMD, yielding better signal-to-noise separation in the tested cases.”

This revision makes the scope of the statement more precise and avoids implying that the proposed method has been proven superior to all existing denoising methods. It also removes the overly broad claim regarding computational efficiency because a full runtime comparison with a broader set of representative traditional methods was not conducted in the present study. Similar wording adjustments have been made elsewhere in the manuscript, and the revised parts have been marked in red.

Regarding the comparative experiments, the baseline methods in this study were selected according to the methodological motivation and application context of the proposed approach. The proposed method is developed by improving traditional SVD with CEEMD; therefore, comparisons with traditional SVD and standalone CEEMD are essential for directly demonstrating the contribution of each component in the proposed workflow. In addition, the Robust method was included in the field-data comparison because it is a commonly used MT processing strategy.

We fully agree that wavelet transform, HHT, and other representative traditional denoising methods are also important references for a broader evaluation of MT denoising performance. In particular, HHT is closely related to EMD-based methods, while CEEMD can be regarded as an improved noise-assisted extension of EMD. Thus, including HHT and wavelet-based methods in a future benchmark comparison would provide a more comprehensive understanding of the advantages and applicability of the proposed method. In the revised manuscript, we have added this issue as an important future research direction in the Conclusion. Future work will extend the comparative framework to include wavelet transform, HHT, VMD, and other representative methods, and will evaluate them under different noise types and field conditions using comprehensive indicators such as SNR improvement, MSE, runtime, parameter sensitivity, and applicability.

The revised parts in the manuscript have been marked in red.

The following statement has been added to the Conclusion:

Furthermore, a broader evaluation framework would help to better contextualize the performance and applicability of the proposed method. The present study focused on comparisons with traditional SVD, standalone CEEMD, and the Robust method, which are directly related to the motivation and workflow of the proposed approach. Future research will extend the benchmark evaluation to include other representative denoising methods, such as wavelet transform, Hilbert–Huang transform, and variational mode decomposition, under different noise types and field conditions. Longer continuous and synchronized multichannel MT records will also be analyzed to evaluate the preservation of electric–magnetic relationships after denoising. Accordingly, evaluation criteria such as SNR improvement, MSE, runtime, parameter sensitivity, practical applicability, and frequency-dependent inter-channel coherence will be considered where appropriate to provide a more systematic assessment of MT denoising strategies.

Thank you again for your time and efforts to review our paper.

Attachment Submitted filename: Response to Reviewers-R3.docx

# Peer review: Decision Letter 3

**DOI:** 10.1371/journal.pone.0334468.r007

**Authors:** Feier Chen

3 Sep 2026

Dear Dr. YUE,

Thank you for submitting your manuscript to PLOS One. After careful consideration, we feel that it has merit but does not fully meet PLOS One’s publication criteria as it currently stands. Therefore, we invite you to submit a revised version of the manuscript that addresses the points raised during the review process.

proof reading

Please submit your revised manuscript by Oct 18 2026 11:59PM. If you will need more time than this to complete your revisions, please reply to this message or contact the journal office at plosone@plos.org. When you're ready to submit your revision, log on to https://www.editorialmanager.com/pone/ and select the 'Submissions Needing Revision' folder to locate your manuscript file.

- A letter that responds to each point raised by the academic editor and reviewer(s). You should upload this letter as a separate file labeled 'Response to Reviewers'.
- A marked-up copy of your manuscript that highlights changes made to the original version. You should upload this as a separate file labeled 'Revised Manuscript with Track Changes'.
- An unmarked version of your revised paper without tracked changes. You should upload this as a separate file labeled 'Manuscript'.

If you would like to make changes to your financial disclosure, please include your updated statement in your cover letter. Guidelines for resubmitting your figure files are available below the reviewer comments at the end of this letter.

If applicable, we recommend that you deposit your laboratory protocols in protocols.io to enhance the reproducibility of your results. Protocols.io assigns your protocol its own identifier (DOI) so that it can be cited independently in the future. For instructions see: https://journals.plos.org/plosone/s/submission-guidelines#loc-laboratory-protocols. Additionally, PLOS One offers an option for publishing peer-reviewed Lab Protocol articles, which describe protocols hosted on protocols.io. Read more information on sharing protocols at https://plos.org/protocols?utm_medium=editorial-email&utm_source=authorletters&utm_campaign=protocols.

As the corresponding author, your ORCID iD is verified in the submission system and will appear in the published article. PLOS supports the use of ORCID, and we encourage all coauthors to register for an ORCID iD and use it as well. Please encourage your coauthors to verify their ORCID iD within the submission system before final acceptance, as unverified ORCID iDs will not appear in the published article. *Only*  the individual author can complete the verification step; PLOS staff *cannot*  verify ORCID iDs on behalf of authors.

We look forward to receiving your revised manuscript.

Kind regards,

Feier Chen, Ph.D

Academic Editor

PLOS One

Journal Requirements:

If the reviewer comments include a recommendation to cite specific previously published works, please review and evaluate these publications to determine whether they are relevant and should be cited. There is no requirement to cite these works unless the editor has indicated otherwise.

Please review your reference list to ensure that it is complete and correct. If you have cited papers that have been retracted, please include the rationale for doing so in the manuscript text, or remove these references and replace them with relevant current references. Any changes to the reference list should be mentioned in the rebuttal letter that accompanies your revised manuscript. If you need to cite a retracted article, indicate the article’s retracted status in the References list and also include a citation and full reference for the retraction notice.

[Note: HTML markup is below. Please do not edit.]

Reviewers' comments:

Reviewer's Responses to Questions

**Comments to the Author**

Reviewer #4: All comments have been addressed

**********

2. Is the manuscript technically sound, and do the data support the conclusions?

Reviewer #4: Yes

**********

3. Has the statistical analysis been performed appropriately and rigorously?

Reviewer #4: Yes

**********

4. Have the authors made all data underlying the findings in their manuscript fully available?

The PLOS Data policy

Reviewer #4: Yes

**********

5. Is the manuscript presented in an intelligible fashion and written in standard English?

Reviewer #4: Yes

**********

Reviewer #4: Thank you to the authors for their careful revisions in response to the previous round of review. After carefully examining the revised manuscript and the point-by-point response letter, I can see that the authors have adequately addressed and revised most of the comments I raised previously. The overall quality of the manuscript has improved significantly compared with the R2 version. However, upon a quick check, I still noticed a minor spelling error. For example, on page 7, line 186, “signa” should be corrected to “signal.” I recommend that the authors carefully proofread the entire manuscript before final submission to avoid similar spelling errors.

In addition, the authors indicate that future work will focus on adaptive workflow control, including noise-type identification, parameter selection, and the selection between single-stage and two-stage processing strategies. They also plan to explore lightweight statistical or feature-based classification methods to provide auxiliary decision-making information prior to signal reconstruction. In this regard, I suggest that the authors further consider existing studies using deep learning models, such as CNN and CNN-LSTM, for noise-type identification, signal-quality assessment, and adaptive processing strategies. For example, the relevant studies by Wang et al. (2024, JGR: Solid Earth), Li et al. (2023, Geophysics), and Zhang et al. (2024, IEEE TGRS) may provide useful references. These studies could provide valuable insights for the future development of the proposed framework, particularly in terms of noise identification, processing-strategy selection, and adaptive workflow control.

Overall, the authors have essentially addressed the major concerns I raised previously. After further careful proofreading and correction of the remaining minor errors, the revised manuscript should be able to meet the publication standards of PLOS ONE.

**********

what does this mean?). If published, this will include your full peer review and any attached files.

If you choose “no”, your identity will remain anonymous but your review may still be made public.

**Do you want your identity to be public for this peer review?**  For information about this choice, including consent withdrawal, please see our Privacy Policy

Reviewer #4: No

**********

[NOTE: If reviewer comments were submitted as an attachment file, they will be attached to this email and accessible via the submission site. Please log into your account, locate the manuscript record, and check for the action link "View Attachments". If this link does not appear, there are no attachment files.]

To ensure your figures meet our technical requirements, please review our figure guidelines: https://journals.plos.org/plosone/s/figures

You may also use PLOS’s free figure tool, NAAS, to help you prepare publication quality figures: https://journals.plos.org/plosone/s/figures#loc-tools-for-figure-preparation.

NAAS will assess whether your figures meet our technical requirements by comparing each figure against our figure specifications.

# Author comment: Author response to Decision Letter 4

**DOI:** 10.1371/journal.pone.0334468.r008

3 Sep 2026

Dear Reviewer #4:

We sincerely thank you for your careful evaluation of our revised manuscript and for your positive assessment of the revisions made in response to the previous round of review. We greatly appreciate your recognition of the improvements in the overall quality of the manuscript.

We have carefully considered your further comments and suggestions and have revised the manuscript accordingly. Your thoughtful feedback has helped us further improve the accuracy, clarity, and completeness of the manuscript. To facilitate your review, all modifications have been clearly highlighted in red in the revised manuscript.

We sincerely hope that these revisions have adequately addressed your remaining concerns and that the revised manuscript now meets the publication standards of PLOS ONE.

Most sincerely,

Fuming Qu

The following are our detailed, point-by-point responses to your comments:

Comments:

[1]Thank you to the authors for their careful revisions in response to the previous round of review. After carefully examining the revised manuscript and the point-by-point response letter, I can see that the authors have adequately addressed and revised most of the comments I raised previously. The overall quality of the manuscript has improved significantly compared with the R2 version. However, upon a quick check, I still noticed a minor spelling error. For example, on page 7, line 186, “signa” should be corrected to “signal.” I recommend that the authors carefully proofread the entire manuscript before final submission to avoid similar spelling errors.

Response: We sincerely thank you for bringing this spelling error to our attention, and we apologize for the oversight. We have corrected “signa” to “signal” on page 7, line 186. Following your recommendation, we also carefully proofread the entire manuscript and corrected other spelling and typographical errors identified during this process. All corresponding changes have been highlighted in red in the revised manuscript.

[2]In addition, the authors indicate that future work will focus on adaptive workflow control, including noise-type identification, parameter selection, and the selection between single-stage and two-stage processing strategies. They also plan to explore lightweight statistical or feature-based classification methods to provide auxiliary decision-making information prior to signal reconstruction. In this regard, I suggest that the authors further consider existing studies using deep learning models, such as CNN and CNN-LSTM, for noise-type identification, signal-quality assessment, and adaptive processing strategies. For example, the relevant studies by Wang et al. (2024, JGR: Solid Earth), Li et al. (2023, Geophysics), and Zhang et al. (2024, IEEE TGRS) may provide useful references. These studies could provide valuable insights for the future development of the proposed framework, particularly in terms of noise identification, processing-strategy selection, and adaptive workflow control.

Response: We sincerely thank you for this valuable and insightful suggestion. We agree that deep learning models, such as CNN and CNN-LSTM, offer promising possibilities for noise-type identification, signal-quality assessment, and adaptive processing-strategy selection in MT data processing.

Following your suggestion, we carefully studied and cited the three recommended articles by Wang et al. (2024), Li et al. (2023), and Zhang et al. (2024) [43-45]. We have also revised the discussion of future work in the Conclusion section. In addition to the originally proposed lightweight statistical or feature-based classification methods, the revised manuscript now identifies deep learning models as a complementary approach for the future development of adaptive workflow control. We further clarify that these approaches will be evaluated in terms of identification accuracy, signal-quality assessment, computational cost, interpretability, and training-data requirements to determine their suitability for noise identification and adaptive processing-strategy selection within the proposed SVD-CEEMD framework.

This suggestion has enabled us to broaden the scope of our future research and present a more comprehensive discussion of the potential development of the proposed framework.

The revised portions of the manuscript are marked in red:

Regarding the limitations and future development of the proposed method, similar to other current denoising approaches, slight attenuation of the underlying signals may still occur after processing. In addition, large-scale noise and impulsive noise do not always co-occur in real MT data; therefore, applying the complete two-stage processing procedure to all data segments may occasionally lead to over-processing. Future work will focus on adaptive workflow control, including noise-type identification, parameter selection, and the choice between single-stage and two-stage processing. To provide auxiliary decision information prior to signal reconstruction, two complementary approaches will be investigated. Lightweight statistical or feature-based classification methods will be considered because of their computational efficiency, interpretability, and limited reliance on prior training data. Meanwhile, recent studies have demonstrated the potential of deep learning models, including CNN and CNN-LSTM architectures, for MT noise identification and suppression [43-45]. These approaches will be evaluated in terms of identification accuracy, signal-quality assessment, computational cost, interpretability, and training-data requirements to determine their suitability for adaptive processing-strategy selection within the proposed SVD-CEEMD framework.

Thank you again for your time and efforts to review our paper.

Attachment Submitted filename: Response to Reviewers-R4.docx

# Peer review: Decision Letter 4

**DOI:** 10.1371/journal.pone.0334468.r009

**Authors:** Feier Chen

14 Sep 2026

Research on magnetotelluric signal noise separation based on improved SVD

PONE-D-25-11846R4

Dear Dr. YUE,

We’re pleased to inform you that your manuscript has been judged scientifically suitable for publication and will be formally accepted for publication once it meets all outstanding technical requirements.

Within one week, you’ll receive an e-mail detailing the required amendments. When these have been addressed, you’ll receive a formal acceptance letter and your manuscript will be scheduled for publication.

An invoice will be generated when your article is formally accepted. Please note, if your institution has a publishing partnership with PLOS and your article meets the relevant criteria, all or part of your publication costs will be covered. Please make sure your user information is up-to-date by logging into Editorial Manager at Editorial Manager® and clicking the ‘Update My Information' link at the top of the page. For questions related to billing, please contact billing support.

If your institution or institutions have a press office, please notify them about your upcoming paper to help maximize its impact. If they’ll be preparing press materials, please inform our press team as soon as possible -- no later than 48 hours after receiving the formal acceptance. Your manuscript will remain under strict press embargo until 2 pm Eastern Time on the date of publication. For more information, please contact onepress@plos.org.

Kind regards,

Feier Chen, Ph.D

Academic Editor

PLOS One

Additional Editor Comments (optional):

Reviewers' comments:

Reviewer's Responses to Questions

**Comments to the Author**

Reviewer #4: (No Response)

**********

2. Is the manuscript technically sound, and do the data support the conclusions?

Reviewer #4: (No Response)

**********

3. Has the statistical analysis been performed appropriately and rigorously?

Reviewer #4: (No Response)

**********

4. Have the authors made all data underlying the findings in their manuscript fully available?

The PLOS Data policy

Reviewer #4: (No Response)

**********

5. Is the manuscript presented in an intelligible fashion and written in standard English?

Reviewer #4: (No Response)

**********

Reviewer #4: Thanks very much for the responses and revisions. The authors have comprehensively responded to my review comments and updated the manuscript properly. Accordingly, I recommend the paper for acceptance.

**********

what does this mean?). If published, this will include your full peer review and any attached files.

If you choose “no”, your identity will remain anonymous but your review may still be made public.

**Do you want your identity to be public for this peer review?**  For information about this choice, including consent withdrawal, please see our Privacy Policy

Reviewer #4: No

**********

# Editor report: Acceptance letter

**DOI:** 10.1371/journal.pone.0334468.r010

**Authors:** Feier Chen

PONE-D-25-11846R4

PLOS One

Dear Dr. YUE,

I'm pleased to inform you that your manuscript has been deemed suitable for publication in PLOS One. Congratulations! Your manuscript is now being handed over to our production team.

At this stage, our production department will prepare your paper for publication. This includes ensuring the following:

* All references, tables, and figures are properly cited

* All relevant supporting information is included in the manuscript submission,

* There are no issues that prevent the paper from being properly typeset

You will receive further instructions from the production team, including instructions on how to review your proof when it is ready. Please keep in mind that we are working through a large volume of accepted articles, so please give us a few days to review your paper and let you know the next and final steps.

Lastly, if your institution or institutions have a press office, please let them know about your upcoming paper now to help maximize its impact. If they'll be preparing press materials, please inform our press team within the next 48 hours. Your manuscript will remain under strict press embargo until 2 pm Eastern Time on the date of publication. For more information, please contact onepress@plos.org.

You will receive an invoice from PLOS for your publication fee after your manuscript has reached the completed accept phase. If you receive an email requesting payment before acceptance or for any other service, this may be a phishing scheme. Learn how to identify phishing emails and protect your accounts at https://explore.plos.org/phishing.

If we can help with anything else, please email us at customercare@plos.org.

Thank you for submitting your work to PLOS One and supporting open access.

Kind regards,

PLOS One Editorial Office Staff

on behalf of

Dr. Feier Chen

Academic Editor

PLOS One
